# Structural diversity using amino acid “Customizable Units”: conversion of hydroxyproline (Hyp) into nitrogen heterocycles

**DOI:** 10.1007/s00726-022-03159-z

**Published:** 2022-04-12

**Authors:** Dácil Hernández, Marina Porras, Alicia Boto

**Affiliations:** Instituto de Productos Naturales y Agrobiología del CSIC, Avda. Astrofísico Francisco Sánchez, 3, 38206 La Laguna, Tenerife Spain

**Keywords:** Customizable units, Structural diversity, *N*-Acyliminium ion, Radical fragmentation, Hydroxyproline, Nitrogen heterocycles, Amino-δ-lactams, Alkaloids, Iminosugars

## Abstract

The ability of amino acid “*customizable units*” to generate structural diversity is illustrated by the conversion of 4-hydroxyproline (Hyp) units into a variety of nitrogen heterocycles. After a first common step, where the unit underwent a one-pot decarboxylation–alkylation reaction to afford 2-alkylpyrrolidines with high stereoselectivity, a divergent step was carried out. Thus, the deprotected 4-hydroxy group was used either to initiate a radical scission that afforded aliphatic β-amino aldehydes, or to carry out an elimination reaction, to give 2-alkyl-2,5-dihydro-1H-pyrroles. In the first case, the amines underwent a tandem reductive amination–cyclization to afford β-amino-δ-lactams, an efficient rigidifying unit in peptides. Different lactam N-substituents, such as alkylamines, peptides, and alkenyl chains suitable for olefin metathesis were introduced this way. In the second case, the pyrrole derivatives were efficiently converted into alkaloid and iminosugar derivatives in good global yields and with excellent stereoselectivity.

**Supplementary Information:**

The online version contains supplementary material available at 10.1007/s00726-022-03159-z.

## Introduction

**Scheme 1:**
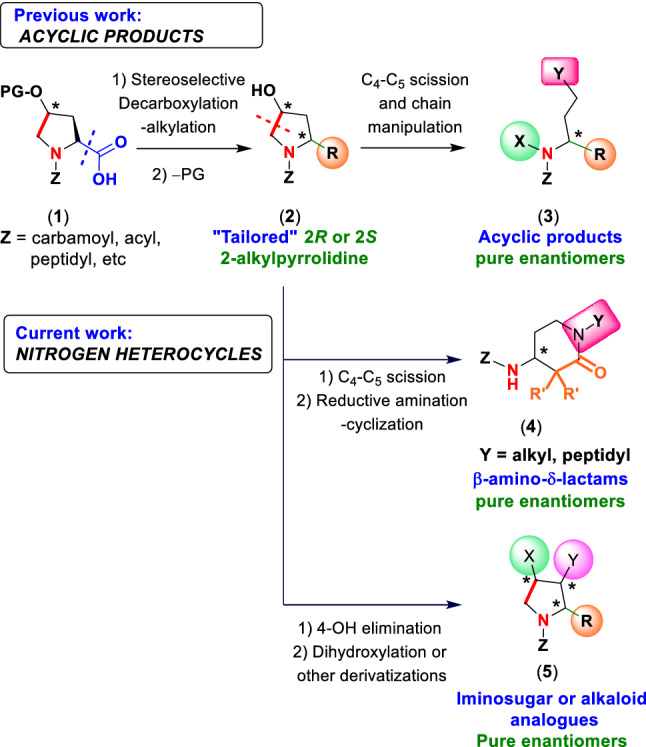
Conversion of 4-hydroxyproline (Hyp) customizable unit into structurally diverse nitrogen heterocycles

The amino acid “*customizable units*” have proven very useful to create structural diversity, particularly in the site-selective modification of peptides, where these units are converted into new residues with tailor-made functions. Different *customizable units* such as glycine, serine, threonine, glutamic acid, proline or hydroxyproline have been described (Boto et al. 2021). We have introduced 4-hydroxyproline as a versatile building block for the production of unusual amino acids (Romero-Estudillo et al. 2015; Cuevas et al. 2021), including *N*-alkyl derivatives (Saavedra et al. 2019, 2020), and reported its use as a “doubly customizable unit” (Hernández et al. 2021), as shown in Scheme 1. In a first step, the substrates **1** underwent a decarboxylation-alkylation reaction to afford 2-alkyl pyrrolidines **2** with high stereoselectivity. In a second step, the 4-hydroxy group was deprotected and a radical scission-oxidation took place to give β-amino aldehydes. These intermediates were manipulated to yield acyclic compounds **3**, which possessed amino groups or longer carbon chains.

Herein we report other applications of this derivatization strategy, to obtain nitrogen heterocycles present in bioactive compounds (Scheme 1), such as β-amino-δ-lactams **4** and alkaloid and iminosugar analogues **5**.

Thus, after formation of different 2-alkyl pyrrolidines **2**, an oxidative radical scission followed by a tandem reductive amination-cyclization was explored, to obtain β-amino-δ-lactams **4**, which are useful rigidifying motifs in peptide chemistry (Weber et al. 2000) and precursors or components of several drugs (Lepovitz et al. 2020; Ungashe et al. 2020; Davies et al. 2012; Chakravarty et al. 2007; Chan-Chun-Kong et al. 2004).

Moreover, the structural diversity would be expanded by elimination of the 4-hydroxy group, to give 2-alkyl-2,5-dihydro-1H-pyrroles, which can be readily converted into iminosugar and alkaloid analogues **5** (Jin et al. 2009; Blunt et al. 2009; Butler et al. 2008; Boto et al. 2009). As reported before, structural diversity could be translated into biological diversity (Cuevas et al. 2021; Pavlinov et al. 2019; Galloway et al. 2014).

## Results and discussion

**Scheme 2:**
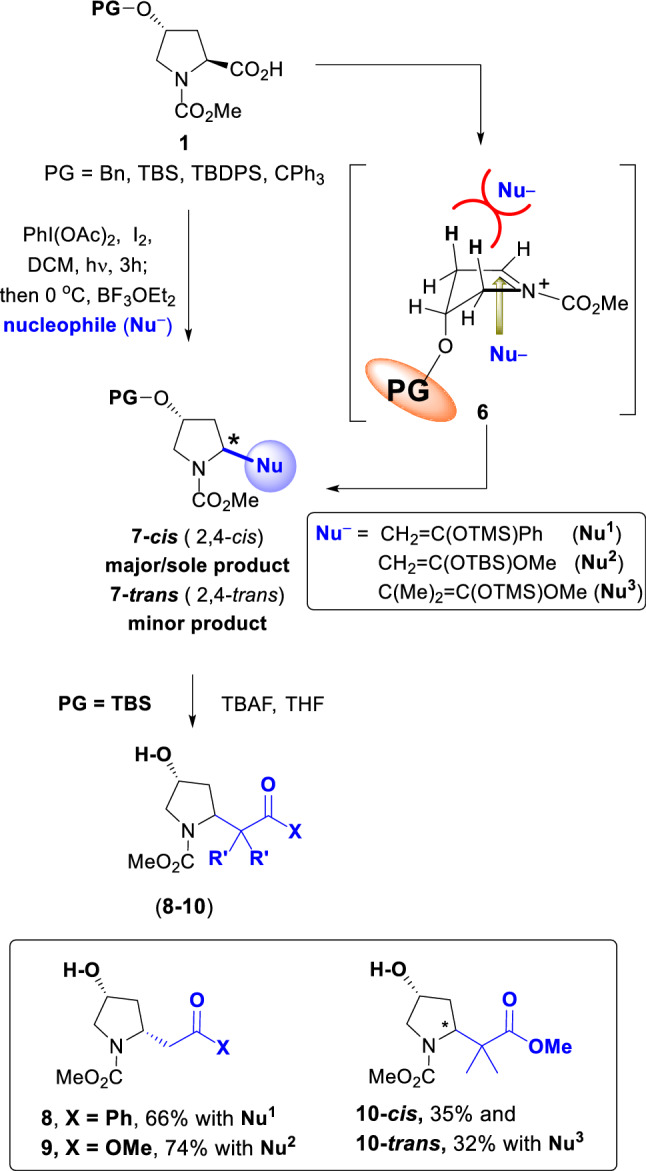
Simplified procedure for the scission-alkylation and 4-hydroxy group deprotection

In our previous work, the protection of the 4-hydroxyl moiety in substrate **1** with benzyl, TBS and TBDPS groups was studied (Hernández et al. 2021), as well as its influence in the stereochemical outcome of the decarboxylation–alkylation reaction (conversion **1** → **7, **Scheme 2). In this conversion, a stereoelectronic effect described by Woerpel generated mainly the 2,4-*cis* product **7-cis** (Smith and Woerpel 2006; Bonger et al. 2008). Thus, substrate **1** underwent an oxidative radical decarboxylation when treated with (diacetoxyiodo)benzene and iodine and then irradiated with visible light. An intermediate 2-acetoxypyrrolidine was formed, which generated the iminium ion **6** on addition of a Lewis acid. This ionic intermediate presented an envelope conformation, where the approach of the nucleophile was hindered by the axial hydrogens opposite to the OP group. When small nucleophiles were used, the 2,4-*cis* product **7-cis** was obtained exclusively, independently of the size of the protecting group. However, when bulky nucleophiles were chosen, and the PG was also bulky, a mixture of the **7-cis** and **7-trans** isomers was obtained. In spite of that, the *cis*-isomer was still the major one (Hernández et al. 2021).

We adapted this strategy to obtain the new products **8–10**. In this paper, we report a novel simplified procedure where the decarboxylation-alkylation and the hydroxyl group deprotection were carried out without isolation of the intermediates. In the example shown in Scheme 2, the TBDMS-protected substrate **1** (PG = TBDMS) underwent the scission-alkylation reaction, using the silyl enol ether **Nu**^**1**^ or the silyl ketenes **Nu**^**2**^ and **Nu**^**3**^. The resulting pyrrolidines were not purified, but treated with TBAF in THF to afford the alcohols **8**, **9** and **10-cis/10-trans**. To our satisfaction, the reaction with **Nu**^**1**^ gave only the 2,4-*cis* isomer **8**. Interestingly, the smaller ketene **Nu**^**2**^ gave exclusively the 2,4-cis isomer **9**, but the larger dimethyl ketene **Nu**^**3**^ afforded a 1:1 ratio of the *cis*:*trans* products **10** (67% global yield).

**Scheme 3:**
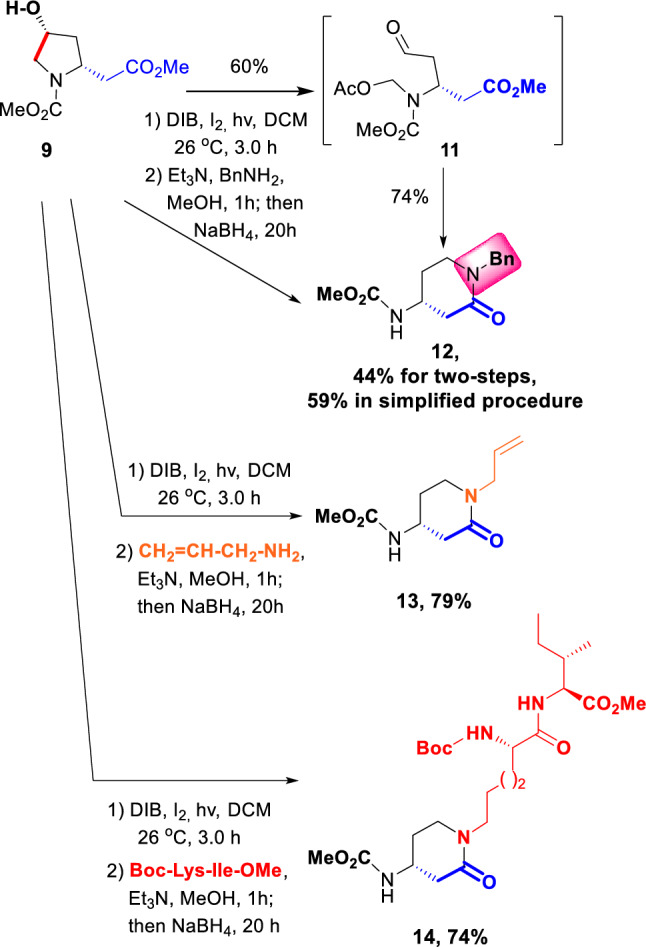
Simplified procedure to obtain β-amino-δ-lactams

The conversion of the β-amino esters **9**, **10-cis** and **10-trans** into a variety of β-amino-δ-lactams took place in two steps. Thus, substrate **9** underwent an oxidative radical scission to give the aldehyde **11** in good yield (Scheme 3). Then a tandem reductive amination-cyclization reaction afforded the lactam **12** as a single enantiomer. Since aldehyde **9** underwent oxidation or other side-reactions over time, we developed a simplified procedure, where the crude scission product was immediately treated under reductive amination conditions. Thus, an increased global yield for lactam **12** (59%) was achieved.

The new amide can also be used to extend the peptide chain. In the conversion **9** → **13** (79%) an allylamine was used as reagent. The resulting olefinic chain can be used in olefin metathesis, to greatly increase the variety of the library. Finally, when the reacting amine belongs to a peptide, a ligation reaction takes place, as illustrated in conversion **9** → **14**. Only one stereoisomer was obtained in the process, indicating that racemization of the lactam stereogenic center (through a retro-Michael reaction prior to scission and readdition of the *N*-carbamate) had not taken place. This results match those reported previously for a related reaction affording α-amino-γ-lactams with retention of their configuration (Romero-Estudillo and Boto 2013). This ligation reaction coupled to the formation of a rigid lactam can be quite useful in the design of bioactive peptide libraries.

**Scheme 4:**
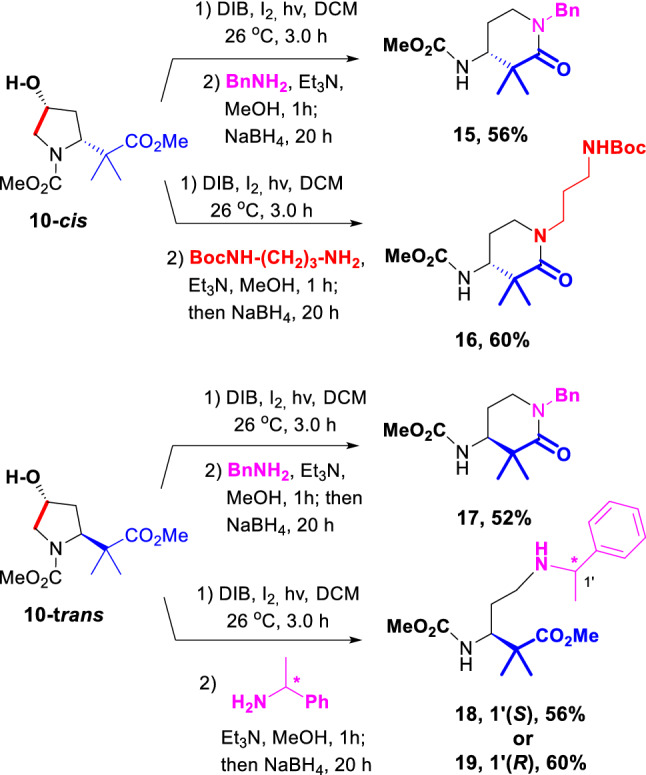
Formation of α,α-disubstituted β-amino-δ-lactams

The process was repeated with substrates **10-cis** and **10-trans** (Scheme 4), providing the α,α-dimethyl β-amino-δ-lactams **15–17** in satisfactory yields (52–60%). In the case of lactams **15** and **17**, the benzyl group can be easily cleaved to afford the deprotected amide, which can be used as a rigidifying moiety in peptide chemistry (Weber et al. 2000). In the conversion **10-cis** → **16** (Scheme 4), a monoprotected diamine was used as reagent, to generate a linker with a terminal *N*-carbamate. The Boc group can be easily removed in acid media, and the resulting amine can be coupled to amino acid or peptide chains, or to other functionalities.

The preparation of α,α-disubstituted β-amino-δ-lactams such as **15–17** is not trivial, due to the steric hindrance posed by the quaternary center to the approach of the amine nucleophile to the carbonyl group. This procedure affords an easy way to obtain these hindered lactams as pure enantiomers. In these cases, the retro-Michael-readdition reaction is not possible, but to rule out other side reactions causing epimerization (such as radical H-abstraction from C-α to give an imine, followed by its isomerization to an encarbamate and reprotonation), the reductive amination was carried out with a chiral amine (1*S*- or 1*R*-methylbenzylamine). Interestingly, the secondary amine was not able to cyclize to a lactam, and thus esters **18** or **19** were obtained. The stereochemistry of the amine had little influence in the reaction outcome, and the yields were similar for both products. In both cases, a single diastereomer was obtained, which confirms that the stereochemical integrity of the product was preserved. The ability to control whether an acyclic or cyclic product is obtained, depending on the amine reagent, could be quite interesting for synthetic purposes.

**Scheme 5:**
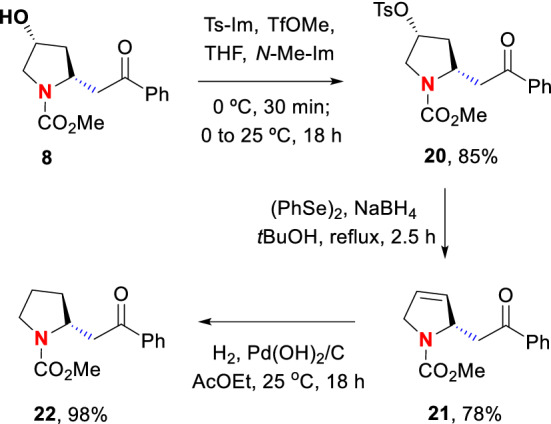
Formation of ( +)-sedamine analogues **21** and **22**

The application of the hydroxyproline unit to the synthesis of alkaloids would require a variation of the previous strategy. Instead of using the 4-hydroxyl group for an oxidative radical scission, it would serve as a leaving group. In the example shown in Scheme 5, the preparation of the methyl carbamate of ( +)-norsedamine from substrate **8** is illustrated**.**

Norsedamine is a five-membered analogue of sedamine, a belladone alkaloid which is clinically used to reduce stomach and intestinal cramping (Tirel et al. 1989; Bates et al. 2002). The generation of alkaloid analogues facilitates the study of structure–activity relationships.

The synthesis used a tosylation in the first step. The tosylate **20** was converted into an intermediate selenide which underwent in situ elimination to afford the dihydropyrrole **21**. The latter was reduced to the pure ( +)−**22** enantiomer in excellent yield.

**Scheme 6:**
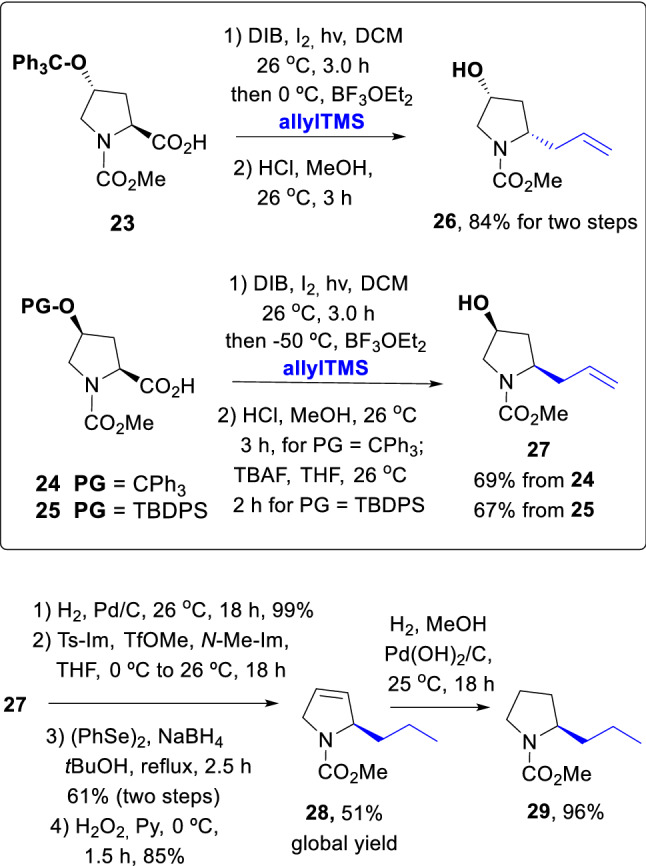
Formation of (−)-coniine analogues **26** and **27**

With the scission-alkylation protocol, both enantiomers of an alkaloid can be obtained using either 4*R*- or 4*S*-hydroxyproline as substrate. The 4*R*- isomer is a natural, low-cost aminoacid, and the 4*S* isomer can be readily prepared therefrom in two efficient steps (Hernández et al. 2021). In Scheme 6, the conversion of Hyp units **23–25** into the 2*R*- or 2*S*-allylpyrrolidines **26** and **27** is shown.

In this case, two different protecting groups were used to compare results. With substrate **23**, the scission-alkylation went smoothly (91%) as well as the deprotection step (92%), to give compound **26** in 84% global yield. In the case of the epimeric substrate **24**, the scission-alkylation proceeded in lower but still good yield (78%), and the deprotection took place in 89% yield. For silyl-protected substrate **25**, the scission took place in 71% yield, but the deprotection afforded product **27** in 95% yield. As a result, both global yields were similar.

Compound **27** was then used to prepare the methyl carbamate of ( −)-norconiine, which is a ring-contracted analogue of ( −)-coniine, the most active alkaloid in the hemlock poison (Passarella et al. 2005; Amat et al. 2003; Blarer et al. 1983). Thus, using the standard protocols commented before, the double bond in substrate **27** was reduced, and the hydroxyl group was tosylated. The tosylate was quickly converted into an intermediate selenide, which was oxidized with hydrogen peroxide to a selenoxide. In-situ elimination afforded the dihydropyrrole **28** in good yield. Hydrogenation of the olefin proceeded quantitatively to give ( −)-norconiine methyl carbamate (**29**). The synthesis of the other norconiine enantiomer (***ent-29***) was carried out using the same methodology, with identical NMR and matching absolute values for the optical activities (see Experimental Section).

The dihydropyrroles are valuable intermediates in the synthesis of other compounds, such as iminosugars and related hydroxylated compounds (Drug Bank, 2021). Many iminosugars are promising glycosidase inhibitors, and some have displayed promising antidiabetic, cytotoxic and antimicrobial activities (Sousa and Alves 2021; Tyrrell et al. 2017; Risseeuw et al. 2013; Horne et al. 2011; Nash et al. 2011; Doddi and Vankar 2007). Therefore, the study of these substances has elicited much interest.

**Scheme 7:**
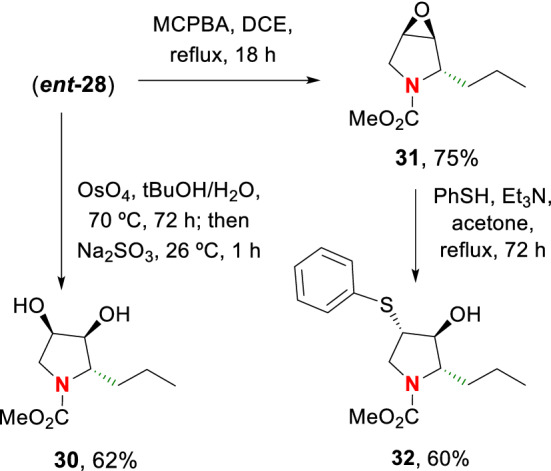
Synthesis of iminosugar derivatives

In the example shown in Scheme 7, **ent-28** was transformed into three iminosugar derivatives. Thus, it underwent dihydroxylation to give compound **30**, and epoxidation to afford compound **31**. The epoxide was cleaved by treatment with thiophene in the presence of triethylamine, affording the thio derivative **32**. In a similar way, other nucleophiles could be introduced to generate a variety of iminosugar analogs.

## Conclusions

The Hyp “customizable unit” can be a valuable intermediate for the formation of nitrogen heterocycles with a variety of lateral chains or functionalities. In a first step, a decarboxylation-alkylation takes place to provide 2-alkyl-4-hydroxyproline derivatives in good yield; remarkably, the 2,4-cis diastereomer is the major or sole isomer. In a second, divergent step, a variety of nitrogen heterocycles are built, either by scission of the C_4_-C_5_ bond followed by reductive amination, or by elimination of the 4-hydroxy group.

In the first case, valuable β-amino-δ-lactams are generated, with different *N*-substituents such as alkylamines, peptides, and alkenyl chains suitable for olefin metathesis. The lactams are useful rigidifying motifs in peptide chemistry and precursors of drugs.

In the second case, dihydropyrroles are formed, that were further functionalized by dihydroxylation, reduction, or epoxidation followed by nucleophile addition, among many possible modifications. In this way, a variety of alkaloid and iminosugar analogues could be generated. This structural diversity from a parent customizable unit could translate into biological diversity, and thus these transformations would be valuable for structure–activity relationship studies.

## Experimental

Commercially available reagents and solvents were of analytical grade or were purified by standard procedures prior to use. All reactions involving air- or moisture-sensitive materials were carried out under a nitrogen atmosphere. Melting points were determined by a hot-stage apparatus and were uncorrected. Optical rotations were measured at the sodium line and ambient temperature (26 °C) in CHCl_3_ solutions. NMR spectra were determined at 500 or 400 MHz for ^1^H and 125.7 or 100.6 MHz for ^13^C, at 25 °C or 70 °C, as stated for each case. Sometimes, due to slower rotamer interconversion at 26 °C, two (or more) sets of signals are visible at room temperature, while only one set of signals (rotamer average) is seen at 70 °C due to faster rotamer interconversion. For some compounds, the ^1^H NMR spectra show some signals as broad bands (br b) due to equilibria between rotamers.

^1^H NMR spectra are reported as follows (s = singlet, d = doublet, t = triplet, dd = doublet of doublets, ddd = doublet of doublet of doublets, q = quartet, m = multiplet, br = broad, br b = broad band, br s = broad singlet; coupling constant(s) in hertz). Mass spectra were recorded using electrospray ionization techniques (ESI) or electronic impact (EI); the latter was determined at 70 eV using an ion trap mass analyzer. Merck silica gel 60 PF254 and 60 (0.063–0.2 mm) were used for preparative thin-layer chromatography (TLC) and column chromatography, respectively. The reagent for TLC analysis was KMnO_4_ in NaOH/K_2_CO_3_ aqueous (aq) solution, and the TLC was heated until development of color.

**Simplified procedure for the scission–alkylation and protecting group removal:** to a solution of the acid substrate (0.2 mmol) in dry dichloromethane (4 mL) were added iodine (25.4 mg, 0.1 mmol) and (diacetoxyiodo)benzene (DIB, 128.9 mg, 0.4 mmol). The resulting solution was stirred for 3 h at 26 °C, under irradiation with visible light (80 W tungsten-filament lamp). Then the reaction mixture was cooled to 0 °C and BF_3_•OEt_2_ (50 µL, 57.0 mg, 0.4 mmol) and the nucleophile (0.6–1.0 mmol) were added. The solution was stirred for 1 h; then was poured into a 1:1 mixture of 10% aqueous Na_2_S_2_O_3_ and saturated aqueous NaHCO_3_ (10 mL) and extracted with CH_2_Cl_2_. The organic layer was dried over sodium sulfate, filtered, and evaporated under vacuum. The crude product was dissolved in THF (3 mL) and treated with TBAF (0.4 mmol, 105.0 mg) for 2 h. Then the reaction mixture was poured into water and extracted with ethyl acetate. After solvent removal, the residue was purified by rotatory chromatography (hexanes/EtOAc) to give the 2-alkyl-4-hydroxypyrrolidine derivatives **8, 9 or 10-cis/10-trans**.

**Synthesis of (2*R*,4*R*)-4-Hydroxy-2-(2-oxo-2-phenylethyl)-*N*-(methoxycarbonyl)pyrrolidine (8):** Substrate 4*R*-((tert-butyldimethylsilyl)oxy)-1-(methoxycarbonyl)-L-proline **1a** was obtained as reported before (Hernández et al. 2021). Product **8** was obtained from the acid **1a** (61.0 mg, 0.2 mmol) according to the simplified procedure, using 1-phenyl-1-trimethylsiloxyethylene (205 µL, 192.3 mg, 1.0 mmol) as the nucleophile in the decarboxylation-alkylation step. After usual work-up and solvent removal, the residue was purified by rotatory chromatography (hexanes/EtOAc, 30:70) yielding the phenyl ketone derivative **8** (35.0 mg, 66%). Product **8** has already been described (Hernández et al. 2021).

**Synthesis of (2*R*,4*R*)-4-(Hydroxy)-2-(2-methoxy-2-oxoethyl)-1-(methoxycarbonyl)pyrrolidine (9):** Obtained from the acid **1a** (61.0 mg, 0.2 mmol) according to the simplified procedure, using 1-methoxy-1-(*tert*-butyldimethylsiloxy)ethene (131 µL, 113.0 mg, 0.6 mmol) as the nucleophile in the decarboxylation-alkylation step. After work-up and solvent evaporation, the residue was purified by rotatory chromatography (hexanes/EtOAc, 30:70) yielding the ester **9** (32.0 mg, 74%) as a viscous oil. [α]_D_^20^ =  + 15 (c 0.48, CHCl_3_). IR (CHCl_3_) ν_max_: 3604, 3437, 1731, 1694, 1455, 1391 cm^–1^. ^1^H NMR (500 MHz, 70 °C, CDCl_3_). Rotamer equilibrium; two sets of signals at 26 °C, one set at 70 °C: δ 4.48–4.41 (m, 1H), 4.28–4.21 (m, 1H), 3.71 (s, 3H), 3.69 (s, 3H), 3.65 (dd, *J* = 11.7, 6.0, Hz, 1H), 3.42 (d, *J* = 11.5 Hz, 1H), 2.99 (br d, *J* = 14.0 Hz, 1H), 2.80 (dd, *J* = 15.7, 9.3 Hz, 1H), 2.29 (ddd, *J* = 13.8, 8.5, 5.5 Hz, 1H), 1.93 (d, *J* = 14.0 Hz, 1H), 1.86 (br s, OH, 1H). ^13^C NMR (125.7 MHz, 70 °C, CDCl_3_): δ 172.3 (C), 155.4 (C), 70.5 (CH), 55.3 (CH_2_), 54.0 (CH), 52.3 (CH_3_), 51.3 (CH_3_), 39.5 (CH_2_), 39.3 (CH_2_). HRMS (ESI-TOF) [M + Na]^+^ calcd for C_9_H_15_NO_5_Na 240.0848, found 240.0843. Anal. Calcd for C_9_H_15_NO_5_: C, 49.76; H, 6.96; N, 6.45. Found: C, 49.73; H, 7.01; N, 6.64.

**(2*R*,4*R*)-2-(1,1-Dimethyl-2-methoxy-2-oxoethyl)-4-(hydroxy)-1-(methoxycarbonyl)pyrrolidine (10-cis) and its (2*S*,4*R*)-diastereomer (10-trans):** Obtained from the acid **1a** (61.0 mg, 0.2 mmol) according to the Simplified Procedure for the Scission-Alkylation and Protecting Group Removal. In the Scission-Alkylation step, (methyltrimethylsilyl)dimethylketene acetal (122 µL, 105.0 mg, 0.6 mmol) was used as the nucleophile. The reaction afforded the 2,4-*cis* compound **10-cis** (17.0 mg, 35%) and its 2,4-trans isomer **10-trans** (16.0 mg, 32%). Products **10-cis/10-trans** have already been described (Hernández et al. 2021).

**General procedure for the scission of the pyrrolidine C**_**4**_**−C**_**5**_** bond:** A solution of 2-alkyl-4-hydroxypyrrolidine (0.2 mmol) in dry DCM (4 mL) was treated with iodine (25.0 mg, 0.1 mmol) and DIB (129.0 mg, 0.4 mmol). The resulting mixture was stirred for 3 h at 26 °C, under irradiation with visible light (80 W tungsten-filament lamp). Then, the reaction mixture was poured into 10% aqueous Na_2_S_2_O_3_ (10 mL) and extracted with CH_2_Cl_2_. The organic layer was dried over sodium sulfate, filtered, and concentrated under vacuum. The residue was purified by chromatography on silica gel (hexanes/ethyl acetate) to give the scission products.

**Methyl (3*R*)-[*N*-(acetoxymethyl)-*N*-(methoxycarbonyl) amino]-5-oxopentanoate** **(11):** Obtained from the 4-hidroxypyrrolidine **9** (43.0 mg, 0.2 mmol) according to the general procedure for the scission of the C_4_-C_5_ bond. After work-up and solvent evaporation, the residue was purified by rotatory chromatography (hexanes/EtOAc, 80:20) yielding the aldehyde **11** (33.0 mg, 60%) as a viscous oil. [α]_D_^20^ = –6 (c 0.37, CHCl_3_). IR (CHCl_3_) ν_max_: 1730, 1572, 1364, 1222, 1015 cm^–1^. ^1^H NMR (500 MHz, 26 °C, CDCl_3_). Rotamer equilibrium; two sets of signals at 26 °C, one set at 70 °C as broad bands: δ 9.71 (s, 1H), 5.43–5.36 (m, 2H), 4.73/4.56 (br b/br b, 1H), 3.73 (br s, 3H), 3.68 (s, 3H), 3.13–3.00 (m, 1H), 2.95–2.80 (m, 2H), 2.78–2.63 (m, 1H), 2.05 (s, 3H). ^13^C NMR (125.7 MHz, 26 °C, CDCl_3_): δ 199.4 (CH), 171.3/170.6 (C), 73.1 (CH_2_), 53.1 (CH_3_), 51.8 (CH_3_), 51.2 (CH), 46.9 (CH_2_), 38.2/37.4 (CH_2_), 21.0 (CH_3_). The carbamate signal was not clearly observed. HRMS (ESI-TOF) [M + Na + MeOH]^+^ calcd for C_12_H_21_NO_8_Na 330.1165, found 330.1166. Anal. Calcd for C_11_H_17_NO_7_: C, 48.00; H, 6.23; N, 5.09. Found: C, 48.25; H, 6.25; N, 4.86.

**(*R*)-1-Benzyl-4-(*N*-methoxycarbonyl)aminopiperidin-2-one (12):** A solution of the aldehyde **11** (27.5 mg, 0.1 mmol) in dry methanol (2.5 mL) was treated with benzylamine (15.3 µL, 15.0 mg, 0.14 mmol) and triethylamine (15 μL, 0.1 mmol). After 1 h at 26 °C, sodium borohydride (6 mg, 0.2 mmol) was added, and the reaction mixture was warmed to 45 °C for 20 h. Then the mixture was allowed to reach room temperature, poured into water and extracted with EtOAc. The organic layer was washed with brine, dried over anhydrous sodium sulfate, and filtered. The solvent was evaporated under vacuum, and the residue was purified by rotatory chromatography (hexanes/EtOAc, 50:50) yielding lactam **12** (19.3 mg, 74%) as a viscous oil. [α]_D_^20^ =  + 8 (c 0.77, CHCl_3_). IR (CHCl_3_) ν_max_: 3440, 1721, 1635, 1515, 1496, 1266, 1071 cm^–1^. ^1^H NMR (500 MHz, 26 °C, CDCl_3_): δ 7.30 (dd, *J* = 7.5, 7.0 Hz, 2H), 7.25 (dd, *J* = 7.5, 7.0 Hz, 1H), 7.20 (d, *J* = 7.0 Hz, 2H), 5.02 (br s, 1H), 4.59 (d, *J* = 15.0 Hz, 1H), 4.54 (d, *J* = 15.0 Hz, 1H), 4.01–3.92 (m, 1H), 3.63 (s, 3H), 3.24–3.20 (m, 2H), 2.80 (ddd, *J* = 17.3, 5.5, 1.5 Hz, 1H), 2.33 (dd, *J* = 17.5, 9.0 Hz, 1H), 2.08–2.02 (m, 1H), 1.78–1.69 (m, 1H). ^13^C NMR (125.7 MHz, 26 °C, CDCl_3_): δ 167.7 (C), 156.2 (C), 136.7 (C), 128.7 (2 × CH), 128.0 (2 × CH), 127.5 (CH), 52.1 (CH_3_), 49.8 (CH_2_), 45.5 (CH), 44.0 (CH_2_), 38.6 (CH_2_), 29.0 (CH_2_). HRMS (ESI-TOF) calcd for C_14_H_18_N_2_O_3_Na [M + Na]^+^ 285.1215, found 285.1216. Anal. Calcd for C_14_H_18_N_2_O_3_: C, 64.11; H, 6.92; N, 10.68. Found: C, 64.05; H, 7.00; N, 10.45.

**Simplified procedure for the Scission of the pyrrolidine C**_**4**_**−C**_**5**_** bond and Reductive Amination**: the 4-hydroxypyrrolidine (0.2 mmol) underwent the general procedure for the scission of the pyrrolidine C_4_ − C_5_ bond. After work-up and solvent evaporation, the residue was dissolved in dry methanol (3 mL) and treated with the amine (0.3 mmol) and triethylamine (30 μL, 0.2 mmol). After 1 h at 26 °C, sodium borohydride (12.0 mg, 0.4 mmol) was added and the reaction mixture was warmed to 45 °C for 20 h. Then the mixture was cooled to room temperature, extracted and purified as commented before. In that way, using benzylamine (31 µL, 30.0 mg, 0.3 mmol) as the amine, the hydroxypyrrolidine **9** (43.0 mg, 0.2 mmol) was transformed into the lactam **12** with improved global yield (31.0 mg, 59%).

**(*R*)-1-Allyl-4-(*N*-methoxycarbonyl)aminopiperidin-2-one (13):** Obtained from the 4-hydroxypyrrolidine **9** (43.5 mg, 0.2 mmol) according to the general Scission and Reductive Amination procedure, using allylamine (21 µL, 16.0 mg, 0.3 mmol) as the amine. After work-up and solvent evaporation, the residue was purified by rotatory chromatography (hexanes/EtOAc, 60:40) yielding the lactam **13** (33.4 mg, 79%) as a syrup. [α]_D_^20^ = +6 (c 0.42, CHCl_3_). ^1^H NMR (500 MHz, 70 °C, CD_3_CN): δ 5.80–5.70 (m, 1H), 5.17–5.10 (m, 2H), 3.95–3.87 (m, 2H), 3.89–3.82 (m, 1H), 3.58 (s, 3H), 3.25 (d, *J* = 5.0 Hz, 1H), 3.24 (d, *J* = 5.1 Hz, 1H), 2.58 (ddd, *J* = 17.2, 5.6, 1.7 Hz, 1H), 2.21 (dd, *J* = 17.2, 8.9 Hz, 1H), 2.05–1.95 (m, 1H) 1.78–1.70 (m, 1H). ^13^C NMR (100.6 MHz, 26 °C, CD_3_CN): δ 168.2 (C), 157.3 (C), 134.3 (CH), 117.1 (CH_2_), 52.4 (CH_3_), 49.4 (CH_2_), 46.7 (CH), 45.0 (CH_2_), 39.1 (CH_2_), 29.6 (CH_2_). HRMS (ESI) [M + Na]^+^ calcd for C_10_H_16_N_2_O_3_Na 235.1059, found 235.1062. Anal. Calcd for C_10_H_16_N_2_O_3_: C, 56.59; H, 7.60; N, 13.20. Found: C, 56.26; H, 7.67; N, 13.30.

**2-[4-(4*R*-(*N*-Methoxycarbonyl)amino-2-oxo-1-piperidinyl)butyl]-2-*N*-(tert-butoxycarbonyl)-L-glycyl-L-isoleucine methyl ester (14):** Obtained from the 4-hydroxypyrrolidine **9** (43.5 mg, 0.2 mmol) according to the general Scission and Reductive Amination procedure, using *N*-(*tert*-butoxycarbonyl)-L-lysyl-L-isoleucine methyl ester (104.5 mg, 0.3 mmol) as the amine. After work-up and solvent evaporation, the residue was purified by rotatory chromatography (hexanes/EtOAc, 40:60) yielding the lactam **14** (78.6 mg, 74%) as a syrup. [α]_D_^20^ = − 4 (c 0.39, CHCl_3_). ^1^H NMR (400 MHz, 70 °C, CD_3_CN): δ 6.85 (d, *J* = 7.6 Hz, 1H), 5.65–5.45 (br b, 2H), 4.38 (dd, *J* = 8.3, 5.9 Hz, 1H), 4.02–3.97 (m, 1H), 3.90–3.82 (m, 1H), 3.69 (s, 3H), 3.61 (s, 3H), 3.36–3.25 (m, 4H), 2.59 (dd, *J* = 16.7, 5.2 Hz, 1H), 2.22 (dd, *J* = 17.1, 8.7 Hz, 1H), 2.09–2.02 (m, 1H), 1.89–1.83 (m, 1H), 1.82–1.70 (m, 2H), 1.65–1.43 (m, 4H), 1.43 (s, 9H), 1.38–1.30 (m, 2H), 1.28–1.16 (m, 1H), 0.95–0.90 (m, 6H). ^13^C NMR (100.6 MHz, 70 °C, CD_3_CN): δ 173.7 (C), 173.3 (C), 168.8 (C), 157.6 (C), 157.1 (C), 80.5 (C), 58.0 (CH), 56.1 (CH), 52.7 (CH_3_), 52.6 (CH_3_), 47.2 (CH), 47.1 (CH_2_), 45.7 (CH_2_), 39.7 (CH_2_), 38.7 (CH), 32.5 (CH_2_), 30.2 (CH_2_), 29.0 (3 × CH_3_), 27.6 (CH_2_), 26.4 (CH_2_), 23.9 (CH_2_), 16.3 (CH_3_), 12.0 (CH_3_). HRMS (ESI) [M + Na]^+^ calcd for C_25_H_44_N_4_O_8_Na 551.3057, found 551.3061. Anal. Calcd for C_25_H_44_N_4_O_8_: C, 56.80; H, 8.39; N, 10.60. Found: C, 56.70; H, 8.29; N, 10.24.

**(*R*)-1-Benzyl-3,3-dimethyl-4-(*N*-methoxycarbonyl)amino piperidin-2-one (15):** Obtained from the 4-(hydroxy)pyrrolidine **10-cis** (49.0 mg, 0.2 mmol) using the simplified procedure for the Scission and Reductive Amination, and benzylamine (31 µL, 30.0 mg, 0.3 mmol) as the amine. After work up and purification using rotatory chromatography (hexanes/EtOAc, 85:15) the lactam **15** was obtained (32.0 mg, 56%) as a viscous oil. [α]_D_^20^ =  + 10 (c 0.67, CHCl_3_). IR (CHCl_3_) ν_max_: 3444, 1721, 1634, 1515, 1453, 1241, 1196 cm^–1^. ^1^H NMR (500 MHz, 26 °C, CDCl_3_): δ 7.32 (t, *J* = 7.0 Hz, 2H), 7.26 (dd, *J* = 7.5, 7.0 Hz, 1H), 7.20 (d, *J* = 7.0 Hz, 2H), 4.72 (br d, *J* = 8.5 Hz, 1H), 4.57 (d, *J* = 14.5 Hz, 1H), 4.53 (d, *J* = 14.5 Hz, 1H), 3.89 (t, *J* = 8.3 Hz, 1H), 3.67 (s, 3H), 3.29–3.23 (m, 1H), 3.21–3.16 (m, 1H), 2.01–1.97 (m, 1H), 1.88–1.80 (m, 1H), 1.33 (s, 3H), 1.21 (s, 3H). ^13^C NMR (125.7 MHz, 26 °C, CDCl_3_): δ 174.4 (C), 156.6 (C), 137.1 (C), 128.6 (2 × CH), 127.9 (2 × CH), 127.4 (CH), 53.8 (CH), 52.2 (CH_3_), 50.4 (CH_2_), 44.0 (CH_2_), 43.0 (C), 25.3 (CH_3_ + CH_2_), 21.2 (CH_3_). HRMS (ESI) [M + Na]^+^ calcd for C_16_H_22_N_2_O_3_Na 313.1528, found 313.1525. Anal. Calcd for C_16_H_22_N_2_O_3_: C, 66.18; H, 7.64; N, 9.65. Found: C, 65.91; H, 7.99; N, 9.47.

**(*R*)-1-[3-(tert-butoxycarbonyl)amino)propyl]-3,3-dimethyl-4-(*N*-methoxycarbonyl)aminopiperidin-2-one (16):** Obtained from the 4-hydroxypyrrolidine **10-cis** (49 mg, 0.2 mmol) according to the general Scission and Reductive Amination procedure, using 1-*tert*-butoxycarbonyl-1,3-propanediamine (49 µL, 49.0 mg, 0.3 mmol) as the amine. After work-up and solvent evaporation, the residue was purified by rotatory chromatography (hexanes/EtOAc, 40:60) yielding the lactam **16** (40.4 mg, 60%) as a syrup. [α]_D_^20^ = −9 (c 0.98, CHCl_3_). ^1^H NMR (400 MHz, 70 °C, CD_3_CN): δ 5.60–5.30 (m, 2H), 3.81 (td, *J* = 9.6, 3.5 Hz, 1H), 3.62 (s, 3H), 3.40–3.23 (m, 4H), 3.00 (q, *J* = 6.4 Hz, 2H), 2.02–1.84 (m, 2H), 1.68–1.61 (m, 2H), 1.43 (s, 9H), 1.14 (s, 3H), 1.10 (s, 3H). ^13^C NMR (100.6 MHz, 70 °C, CD_3_CN): δ 176.0 (C), 158.1 (C), 157.3 (C), 79.6 (C), 55.5 (CH), 52.7 (CH_3_), 45.7 (CH_2_), 45.6 (CH_2_), 44.3 (C), 39.0 (CH_2_), 29.1 (3 × CH_3_), 28.4 (CH_2_), 26.51 (CH_2_), 26.47 (CH_3_), 22.3 (CH_3_). HRMS (ESI) [M + Na]^+^ calcd for C_17_H_31_N_3_O_5_Na 380.2161, found 380.2151. Anal. Calcd for C_17_H_31_N_3_O_5_: C, 57.12; H, 8.74; N, 11.76. Found: C, 57.40; H, 8.65; N, 11.39.

**(*S*)-1-Benzyl-3,3-dimethyl-4-(*N*-methoxycarbonyl)amino piperidin-2-one (17):** Obtained from the 4-(hydroxy)pyrrolidine **10-trans** (49.0 mg, 0.2 mmol) using the simplified procedure for the Scission and Reductive Amination, and benzylamine (31 µL, 30.0 mg, 0.3 mmol) as the amine. After work up and purification using rotatory chromatography (hexanes/EtOAc, 85:15) the lactam **17** was obtained (30.0 mg, 52%) as a viscous oil. [α]_D_^20^ = –9 (c 0.17, CHCl_3_). IR (CHCl_3_) ν_max_: 3444, 1721, 1633, 1515, 1344, 1241, 1063 cm^–1^. ^1^H NMR (500 MHz, 26 °C, CDCl_3_): δ 7.32 (t, *J* = 7.0 Hz, 2H), 7.26 (dd, *J* = 7.5, 7.0 Hz, 1H), 7.20 (d, *J* = 7.0 Hz, 2H), 4.72 (br d, *J* = 8.0 Hz, 1H), 4.57 (d, *J* = 14.8 Hz, 1H), 4.53 (d, *J* = 14.5 Hz, 1H), 3.89 (t, *J* = 8.4 Hz, 1H), 3.67 (s, 3H), 3.29–3.24 (m, 1H), 3.21–3.16 (m, 1H), 2.02–1.97 (m, 1H), 1.88–1.80 (m, 1H), 1.33 (s, 3H), 1.21 (s, 3H). ^13^C NMR (125.7 MHz, 26 °C, CDCl_3_): δ 174.4 (C), 156.5 (C), 137.1 (C), 128.7 (2 × CH), 127.9 (2 × CH), 127.4 (CH), 53.8 (CH), 52.3 (CH_3_), 50.4 (CH_2_), 44.0 (CH_2_), 43.0 (C), 25.3 (CH_3_ + CH_2_), 21.2 (CH_3_). HRMS (ESI) [M + Na]^+^ calcd for C_16_H_22_N_2_O_3_Na 313.1528, found 313.1525. Anal. Calcd for C_16_H_22_N_2_O_3_: C, 66.18; H, 7.64; N, 9.65. Found: C, 66.17; H, 7.41; N, 9.91.

**Methyl (*S*)-3-((methoxycarbonyl)amino)-2,2-dimethyl-5-([(*S*)-1-phenylethyl]amino)pentanoate (18):** Obtained from the 4-hydroxypyrrolidine **10-trans** (49.0 mg, 0.2 mmol) according to the general Scission and Reductive Amination procedure, using (*S*)-1-phenylethan-1-amine (36 µL, 34.0 mg, 0.3 mmol) as the amine. After work-up and solvent evaporation, the residue was purified by rotatory chromatography (hexanes/EtOAc, 30:70) yielding the ester **18** (37.2 mg, 56%) as a syrup. [α]_D_^20^ = − 43 (c 0.38, CHCl_3_). ^1^H NMR (400 MHz, 26 °C, CD_3_CN): δ 7.32–7.28 (m, 4H), 7.24–7.19 (m, 1H), 5.34 (d, *J* = 10.0 Hz, 1H), 3.88 (ddd, *J* = 12.3, 10.0, 2.3 Hz, 1H), 3.69 (q, *J* = 6.6 Hz, 1H), 3.62 (s, 3H), 3.58 (s, 3H), 2.41–2.27 (m, 2H), 1.57–1.49 (m, 1H), 1.37–1.27 (m, 1H), 1.25 (d, *J* = 6.6 Hz, 3H), 1.11 (s, 3H), 1.08 (s, 3H). ^13^C NMR (100.6 MHz, 26 °C, CD_3_CN): δ 177.8 (C), 158.4 (C), 147.3 (C), 129.3 (2 × CH), 127.7 (3 × CH), 59.0 (CH), 55.8 (CH), 52.6 (CH_3_), 52.5 (CH_3_), 47.8 (C), 45.1 (CH_2_), 31.8 (CH_2_), 25.2 (CH_3_), 23.4 (CH_3_), 21.3 (CH_3_). HRMS (ESI) [M + Na]^+^ calcd for C_18_H_28_N_2_O_4_Na 359.1947, found 359.1956. Anal. Calcd for C_18_H_28_N_2_O_4_: C, 64.26; H, 8.39; N, 8.33. Found: C, 64.08; H, 8.36; N, 8.24.

**Methyl (*S*)-3-((methoxycarbonyl)amino)-2,2-dimethyl-5-([(*R*)-1-phenylethyl]amino)pentanoate (19):** Obtained from the 4-hydroxypyrrolidine **10-trans** (49.0 mg, 0.2 mmol) according to the general Scission and Reductive Amination procedure, using (*R*)-1-phenylethan-1-amine (36 µL, 34.0 mg, 0.3 mmol) as the amine. After work-up and solvent evaporation, the residue was purified by rotatory chromatography (hexanes/EtOAc, 30:70) yielding the ester **19** (40.2 mg, 60%) as a syrup. [α]_D_^20^ = + 18 (c 0.30, CHCl_3_). ^1^H NMR (400 MHz, 26 °C, CD_3_CN): δ 7.31–7.29 (m, 4H), 7.24–7.19 (m, 1H), 5.38 (d, *J* = 9.9 Hz, 1H), 3.79 (ddd, *J* = 11.4, 10.5, 2.3 Hz, 1H), 3.68 (q, *J* = 6.6 Hz, 1H), 3.61 (s, 3H), 3.54 (s, 3H), 2.44–2.34 (m, 2H), 1.57–1.48 (m, 1H), 1.37–1.26 (m, 1H), 1.24 (d, *J* = 6.5 Hz, 3H), 1.10 (s, 3H), 1.08 (s, 3H). ^13^C NMR (100.6 MHz, 26 °C, CD_3_CN): δ 177.8 (C), 158.4 (C), 147.5 (C), 129.3 (2 × CH), 127.7 (CH), 127.6 (2 × CH), 59.0 (CH), 56.3 (CH), 52.6 (CH_3_), 52.5 (CH_3_), 47.7 (C), 45.6 (CH_2_), 32.2 (CH_2_), 24.8 (CH_3_), 23.3 (CH_3_), 21.4 (CH_3_). HRMS (ESI) [M + Na]^+^ calcd for C_18_H_28_N_2_O_4_Na 359.1947, found 359.1953. Anal. Calcd for C_18_H_28_N_2_O_4_: C, 64.26; H, 8.39; N, 8.33. Found: C, 64.52; H, 8.21; N, 8.18.

### Synthesis of the methyl carbamate of (+)-norsedamine (22) and its precursors 20 and 21

**(2*R*,4*R*)-2-(2-oxo-2-phenylethyl)-4-(tosyloxy)-*N*-(methoxy carbonyl)pyrrolidine (20):** Methyl triflate (490 µL, 4.5 mmol) was slowly added to a solution of 1-(p-toluenesulfonyl)imidazole (1020.0 mg, 4.5 mmol) in dry THF (6 mL), at 0 °C and under nitrogen atmosphere. The mixture was stirred for 0.5 h and then a solution of product **8** (789.0 mg, 3.0 mmol) in THF (6 mL) and *N-*methylimidazole (369.0 mg, 360 µL, 4.5 equiv) were added. The mixture was stirred at room temperature overnight, then poured into water and extracted with EtOAc. The organic layer was dried and concentrated as usual, and the residue was purified by chromatography on silica gel (hexane/EtOAc 85:15) to give the tosylate **20** (1060.0 mg, 85%) as a viscous oil. [α]_D_^20^ =  + 20 (c 1.0, CHCl_3_). IR (CHCl_3_) ν_max_: 1686, 1453, 1391, 1176, 1126 cm^–1^. ^1^H NMR (500 MHz, 70 °C, CDCl_3_) rotamer equilibrium. Two sets of signals at 26 °C, one set at 70 °C: δ 7.91 (d, *J* = 7.3 Hz, 2H), 7.71 (d, *J* = 8.5 Hz, 2H), 7.54 (dd, *J* = 7.5, 7.0 Hz, 2H), 7.45 (dd, *J* = 8.0, 7.5 Hz, 2H), 7.23 (d, *J* = 8.0 Hz, 2H), 5.11–5.07 (m, 1H), 4.48–4.43 (m, 1H), 3.72–3.69 (m, 2H), 3.67 (s, 3H), 3.69–3.64 (m, 1H), 3.19 (dd, *J* = 15.9, 10.2 Hz, 1H), 2.39 (s, 3H), 2.33 (ddd, *J* = 14.5, 8.5, 5.0 Hz, 1H), 2.08 (d, *J* = 14.5 Hz, 1H). ^13^C NMR (125.7 MHz, 70 °C, CDCl_3_): δ 198.3 (C), 155.0 (C), 145.0 (C), 137.2 (C), 134.4 (C), 133.1 (CH), 130.0 (2 × CH), 128.6 (2 × CH), 128.1 (2 × CH), 127.7 (2 × CH), 79.9 (CH), 53.7 (CH), 52.7 (CH_2_), 52.4 (CH_3_), 43.1 (CH_2_), 37.1 (CH_2_), 21.4 (CH_3_). MS *m/z* (rel intensity) 417 (M^+^, 2), 126 ([*N*-methoxycarbonyl)pyrrole + H]^+^, 100). HRMS (EI) [M]^+^ calcd for C_21_H_23_NO_6_S, 417.1246; found, 417.1238; [*N*-methoxycarbonyl)pyrrole + H]^+^ calcd for C_6_H_8_NO_2_, 126.0555; found, 126.0551. Anal. Calcd for C_21_H_23_NO_6_S: C, 60.42; H, 5.55; N, 3.36; S, 7.68. Found: C, 60.25; H, 5.66; N, 3.33; S, 7.41.

**(2*S*)-2-(2-oxo-2-phenylethyl)-*N*-(methoxycarbonyl)-2,5-dihydro-1H-pyrrole (21):** Sodium borohydride (91.0 mg, 2.4 mmol) was added to a solution of diphenyl diselenide (394.0 mg, 1.3 mmol) in *tert*-butanol (10 mL) and the resulting mixture was refluxed until the disappearance of the yellow color. Then a solution of the tosyl pyrrolidine **20** (830.0 mg, 2.0 mmol) in *tert*-butanol (10 mL) was added, and the mixture was stirred under reflux for 2.5 h. Then it was cooled to room temperature, poured into water and extracted with EtOAc. The organic extract was dried over anhydrous Na_2_SO_4_ and concentrated under vacuum. The selenide was unstable and underwent elimination without further treatment. Purification by chromatography on silica gel (hexane/EtOAc 80:20) afforded the dihydropyrrole **21** (380.0 mg, 78%) as a viscous oil. [α]_D_^20^ =  + 110 (c 0.62, CHCl_3_). IR (CHCl_3_) ν_max_: 1688, 1454, 1392, 1197, 1128 cm^–1^. ^1^H NMR (500 MHz, 70 °C, CDCl_3_) Rotamer equilibrium; two sets of signals at 26 °C, one set at 70 °C: δ 7.96 (d, *J* = 7.3 Hz, 2H), 7.54 (t, *J* = 7.5 Hz, 1H), 7.44 (t, *J* = 7.7 Hz, 2H), 5.96–5.91 (m, 1H), 5.81–5.76 (m, 1H), 5.08–5.00 (m, 1H), 4.28–4.18 (m, 1H), 4.08 (br d, *J* = 13.0 Hz, 1H), 3.95–3.75 (m, 1H), 3.71 (s, 3H), 3.00 (dd, *J* = 15.5, 9.0 Hz, 1H). ^13^C NMR (125.7 MHz, 70 °C, CDCl_3_): δ 198.3 (C), 155.2 (C), 137.4 (C), 133.0 (CH), 130.3 (CH), 128.6 (2 × CH), 128.2 (2 × CH), 125.3 (CH), 61.7 (CH), 53.4 (CH_2_), 52.2 (CH_3_), 43.3 (CH_2_). MS *m/z* (rel intensity) 245 (M^+^, 10), 105 ([PhCO]^+^, 100). HRMS (EI) [M]^+^ calcd for C_14_H_15_NO_3_, 245.1052; found, 245.1045; [PhCO]^+^ calcd for C_7_H_5_O, 105.0340; found, 105.0341. Anal. Calcd for C_14_H_15_NO_3_: C, 68.56; H, 6.16; N, 5.71. Found: C, 68.52; H, 6.13; N, 5.99.

**(2*R*)-2-(2-oxo-2-phenylethyl)-*N*-(methoxycarbonyl) pyrrolidine (22):** The dihydropyrrole **21** (49.0 mg, 0.2 mmol) was dissolved in dry EtOAc (3 mL) and 10% Pd(OH)_2_/C (40.0 mg) was added. The resulting mixture was stirred overnight under hydrogen atmosphere (1 atm). Then it was filtered over Celite and the filtrate was concentrated under vacuum to afford the methyl carbamate of ( +)-norsedamine **22** (48.5 mg, 98%) as a viscous oil. [α]_D_^20^ =  + 27 (c 0.26, CHCl_3_). IR (CHCl_3_) ν_max_: 1682, 1454, 1390, 1216, 1124 cm^–1^. ^1^H NMR (500 MHz, 70 °C, CDCl_3_): δ 7.29–7.20 (m, 2H), 7.20–7.10 (m, 3H), 3.90–3.82 (m, 1H), 3.67 (s, 3H), 3.45 (ddd, *J* = 11.0, 8.0, 7.5 Hz, 1H), 3.33 (ddd, *J* = 11.0, 7.5, 5.0 Hz, 1H), 2.65–2.57 (m, 2H), 2.14–2.07 (m, 1H), 2.00–1.90 (m, 1H), 1.89–1.82 (m, 1H), 1.82–1.76 (m, 1H), 1.72–1.61 (m, 2H). ^13^C NMR (125.7 MHz, 70 °C, CDCl_3_): δ 203.7 (C), 155.8 (C), 142.1 (C), 128.4 (2 × CH), 125.8 (3 × CH), 57.6 (CH), 51.9 (CH_3_), 46.5 (CH_2_), 36.0 (CH_2_), 32.6 (CH_2_), 30.6/23.7 (CH_2_). MS *m/z* (rel intensity) 233 ([M– COPh]^+^, 3), 128 ([M – CH_2_COPh]^+^, 100). HRMS (EI) [M– COPh]^+^ calcd for C_7_H_12_NO_2_, 142.0868; found, 142.0861; [M – CH_2_COPh]^+^ calcd for C_6_H_10_NO_2_, 128.0712; found, 128.0716. Anal. Calcd for C_14_H_17_NO_3_: C, 68.00; H, 6.93; N, 5.66. Found: C, 68.17; H, 6.^63^; N, 5.99.

**Scission-allylation reaction of substrates 23–25 to provide 2-allylpyrrolidines 26 and 27:** The synthesis of the acid precursors following standard methodologies is described in the Supporting information. Reaction products **26** and **27** are known (Hernández et al. 2021), but their synthesis from substrates **23–25** is new and is described below.

**(2*S*,4*R*)-2-Allyl-4-hydroxy-1-methoxycarbonyl pyrrolidine (26):** A solution of the acid **23** (430.0 mg, 1.0 mmol) in dry dichloromethane (15 mL) was treated with iodine (127.0 mg, 0.5 mmol) and (diacetoxyiodo)benzene (DIB, 645.0 mg, 2.0 mmol). The solution was stirred for 3 h at 26 °C, under irradiation with visible light (80 W tungsten-filament lamp). Then the reaction mixture was cooled to 0 °C and BF_3_•OEt_2_ (250 µL, 2.0 mmol) and allyltrimethylsilane (0.8 mL, 5.0 mmol) were added; the stirring proceeded for 1 h. After usual work-up, the crude product was dissolved in 2 M methanolic HCl (10 mL) and stirred for 3 h. The solution was poured slowly into saturated sodium bicarbonate and extracted with dichloromethane. The residue was purified by column chromatography (hexanes:AcOEt 30:70), yielding product **26** (155.5 mg, 84%) as a viscous oil. Product **26** has already been described (Hernández et al. 2021). Four reactions were run in parallel and purified simultaneously to obtain the precursor for the norconiine synthesis.

**(2*R*,4*S*)-2-Allyl-4-hydroxy-1-methoxycarbonyl pyrrolidine (27):** Obtained from acid **24** (430.0 mg, 1.0 mmol) using a procedure similar to the one developed for compound **24**, but using a temperature of –50 °C in the nucleophile addition step. After the deprotection of the 4-OH group and purification by chromatography, compound **27** was obtained (128.0 mg, 69%) as a viscous oil. An alternative procedure using acid **25** as starting material (425.0 mg, 1.0 mmol) where the 4-OH deprotection step was carried out with TBAF (525.0 mg, 2.0 mmol) in THF (10 mL) for 2 h. After aqueous work-up and solvent evaporation, the residue was purified as before, affording compound **27** in a similar yield (124.0 mg, 67%). Product **27** has already been described (Hernández et al. 2021). Five reactions were run in parallel and purified simultaneously to obtain the norconiine precursor.

**Synthesis of the methyl carbamate of (-)-norconiine (29):** The conversion of compound **27** into the dihydropyrrole **28** is very similar to that commented for the sedamine precursor **21**, and is therefore commented in the Supporting Information. The conversion of compound **28** into **29** is commented below. Although compound **29** has been reported (Wistrand and Skrinjar 1991), new characterization details are commented herein.

**(2*R*)-2-(propyl)-*N*-(methoxycarbonyl)pyrrolidine (29):** The dihydropyrrole **28** (34.0 mg, 0.2 mmol) was dissolved in dry EtOAc (3 mL) and 10% Pd(OH)_2_/C (40.0 mg) was added. The resulting mixture was stirred overnight under hydrogen atmosphere (1 atm). Then it was filtered over Celite and the filtrate was concentrated under vacuum to afford the methyl carbamate of (-)-norconiine (**29**) (32.4 mg, 96%) as a viscous oil. [α]_D_^20^ = –22 (c 0.34, CHCl_3_). IR (CHCl_3_) ν_max_: 1681, 1455, 1390, 1220, 1118 cm^–1^. ^1^H NMR (500 MHz, 70 °C, CDCl_3_) Rotamer equilibrium; two sets of signals at 26 °C, one set at 70 °C: δ 3.84–3.79 (m, 1H), 3.68 (s, 3H), 3.43 (dt, *J* = 10.5, 7.5, 7.5 Hz, 1H), 3.32 (ddd, *J* = 11.0, 7.5, 5.0 Hz, 1H), 1.95–1.87 (m, 1H), 1.88–1.82 (m, 1H), 1.81–1.74 (m, 1H), 1.72 (br b, 1H, OH), 1.67–1.63 (m, 1H), 1.35–1.27 (m, 3H), 0.93 (t, *J* = 7.1 Hz, 3H). ^13^C NMR (125.7 MHz, 70 °C, CDCl_3_): δ 155.7 (C), 57.6 (CH), 51.9 (CH_3_), 46.4 (CH_2_), 36.7 (CH_2_), 30.4 (CH_2_), 23.6 (CH_2_), 19.4 (CH_2_), 13.9 (CH_3_). HRMS (ESI-TOF) *m/z* [M + Na]^+^ calcd for C_9_H_17_NO_2_Na 194.1157; found, 194.1161. Anal. Calcd for C_9_H_17_NO_2_: C, 63.13; H, 10.01; N, 8.18. Found: C, 63.50; H, 9.82; N, 7.83. In the literature, Wistrand LG, Skrinjar, M. (1991) reported the ^1^H and HRMS for this compound, which match our observed data. Their optical activity was described with methanol ([α]_D_^20^ = –69.4 (c 1.0, MeOH), but Blarer and Seebach (1983) reported the optical activity of the closely related *t*-butylcarbamate in chloroform ([α]_D =_  − 34.1 (c 1.1, CHCl_3_).

**Synthesis of the methyl carbamate of (+)-norconiine (ent-29):** The synthesis was repeated from the alcohol **26**, following the same procedures to obtain **ent-28**, which was finally transformed into ( +)-norconiine (**ent-29**). The spectroscopic data of the intermediates and **ent-29** were identical to those of its enantiomers, as shown in the Supplementary Information. The value of the optical activities matched for both enantiomers. Thus, [α]_D_^20^ =  + 39 (c 0.25, CHCl_3_) for **ent-28a**; [α]_D_^20^ =  + 12 (c 0.41, CHCl_3_) for **ent-28b**; [α]_D_^20^ =  + 70 (c 0.17, CHCl_3_) for **ent-28** and [α]_D_^20^ =  + 23 (c 0.35, CHCl_3_) for **ent-29**. For ( +)-norconiine **ent-29**, Anal. Calcd for C_9_H_17_NO_2_: C, 63.13; H, 10.01; N, 8.18. Found: C, 63.38; H, 10.14; N, 8.06.

**Synthesis of iminosugar derivatives. (2*S*,3*S*,4*R*)-3,4-dihydroxy-2-propyl-*N*-(methoxycarbonyl)pyrrolidine (30):** Pyrrolidine **ent-28** (17.0 mg, 0.1 mmol) was added to a solution of OsO_4_ (25.4 mg, 0.1 mmol) in tBuOH/H_2_O (5 mL, 1/1) and the mixture was stirred at 70 °C for 72 h. Then it was allowed to reach room temperature and sodium sulfite (126.0 mg) was added to continue stirring for one hour. The mixture was then poured into H_2_O and extracted with EtOAc. The organic extract was dried over anhydrous Na_2_SO_4_ and concentrated under vacuum. The residue was purified by chromatography on silica gel (CH_2_Cl_2_/MeOH, 98:2) to give the diol **30** (12.7 mg, 62%) as a viscous oil. [α]_D_^20^ =  + 28 (c 0.10, CHCl_3_). IR (CHCl_3_) ν_max_: 3553, 3401, 1688, 1455, 1392, 1226, 1090 cm^–1^. ^1^H NMR (500 MHz, 70 °C, CD_3_OD) Rotamer equilibrium; two sets of signals at 26 °C, one set at 70 °C: δ 4.23–4.18 (m, 1H), 3.88 (dd, *J* = 4.0, 2.5 Hz, 1H), 3.68–3.63 (m, 1H), 3.67 (s, 3H), 3.48 (dd, *J* = 11.3, 6.3 Hz, 1H), 3.36 (dd, *J* = 11.2, 6.1 Hz, 1H), 1.71–1.63 (m, 1H), 1.47–1.34 (m, 3H), 0.93 (t, *J* = 7.3 Hz, 3H). ^13^C NMR (125.7 MHz, 26 °C, CD_3_OD): δ 158.0/157.8 (C), 76.5/75.8 (CH), 71.2/70.7 (CH), 65.1/64.6 (CH), 53.0/52.9 (CH_3_), 51.5/51.1 (CH_2_), 35.9/35.3 (CH_2_), 20.0 (CH_2_), 14.4 (CH_3_). MS *m/z* (rel intensity) 204 ([M + H]^+^, 6), 161 ([M – propyl + H]^+^, 100). HRMS (EI) [M + H]^+^ calcd for C_9_H_18_NO_4_, 204.1236; found, 204.1238; [M – propyl + H]^+^ calcd for C_6_H_11_NO_4_, 161.0688; found, 161.0886. Anal. Calcd for C_9_H_17_NO_4_: C, 53.19; H, 8.43; N, 6.89. Found: C, 53.32; H, 8.63; N, 6.99.

**(2*S*,3*S*,4*R*)-3,4-epoxy-2-propyl-*N*-(methoxycarbonyl)pyrrolidine (31):** 3-Chloroperbenzoic acid (122.0 mg, 0.7 mmol) was added to a solution of the olefin **ent-28** (100.0 mg, 0.6 mmol) in 1,2-dichloroethane (10 mL) and the mixture was refluxed with stirring overnight. Then the mixture was poured into aqueous saturated NaHCO_3_ and extracted with CH_2_Cl_2_. The organic extract was dried over anhydrous Na_2_SO_4_ and concentrated under vacuum. The residue was purified by chromatography on silica gel (hexanes/EtOAc, 85:15) to give the epoxide **31** (81.6 mg, 75%) as a viscous oil: [α]_D_^20^ =  + 42 (c 0.67, CHCl_3_). IR (CHCl_3_) ν_max_: 1690, 1457, 1388, 1214, 1123 cm^–1^. ^1^H NMR (500 MHz, 70 °C, CDCl_3_) Rotamer equilibrium; two sets of signals at 26 °C, one set at 70 °C: δ 4.11–4.00 (br b, 1H), 4.00–3.80 (br b, 1H), 3.67 (s, 3H), 3.56 (d, *J* = 3.0 Hz, 1H), 3.42 (d, *J* = 3.0 Hz, 1H), 3.26 (dd, *J* = 13.0, 1.0 Hz, 1H), 1.62–1.51 (m, 2H), 1.41 (sextuplet, *J* = 7.5 Hz, 2H), 0.97 (t, *J* = 7.5 Hz, 3H). ^13^C NMR (125.7 MHz, 70 °C, CDCl_3_): δ 156.1 (C), 58.2 (CH), 58.0 (CH), 54.5/54.0 (CH), 52.2 (CH_3_), 47.1 (CH_2_), 33.4/32.9 (CH_2_), 18.7 (CH_2_), 14.0 (CH_3_). MS *m/z* (rel intensity) 185 (M^+^, 10), 142 ([M – propyl]^+^, 100). HRMS (EI) [M]^+^ calcd for C_9_H_15_NO_3_, 185.1052; found, 185.1051; [M – propyl]^+^ calcd for C_6_H_8_NO_3_, 142.0504; found, 142.0498. Anal. Calcd for C_9_H_15_NO_3_: C, 58.36; H, 8.16; N, 7.56. Found: C, 58.49; H, 8.12; N, 7.47.

**(2*S*,3*S*,4*S*)-3-Hydroxy-4-phenylthio-2-propyl-*N*-(methoxycarbonyl)pyrrolidine (32):** The epoxide **31** (18.5 mg, 0.1 mmol) was dissolved in dry acetone (5 mL) and treated with PhSH (31 µL, 33.0 mg, 0.3 mmol) and Et_3_N (42 µL, 30.3 mg, 0.3 mmol). The mixture was stirred at 50 °C for 72 h. Then it was poured into H_2_O and extracted with CH_2_Cl_2_. The organic extract was dried over anhydrous Na_2_SO_4_ and concentrated under vacuum. The residue was purified by chromatography on silica gel (hexanes/EtOAc, 80:20) to give the phenylthio derivative **32** (18.0 mg, 60%) as a viscous oil: [α]_D_^20^ =  + 37 (c 0.12, CHCl_3_). IR (CHCl_3_) ν_max_: 3594, 1688, 1454, 1391, 1204, 1123 cm^–1^. ^1^H NMR (500 MHz, 70 °C, CDCl_3_) rotamer equilibrium. Two sets of signals at 26 °C, one set at 70 °C: δ 7.45 (d, *J* = 8.0 Hz, 2H), 7.32 (t, *J* = 7.5 Hz, 2H), 7.28–7.25 (m, 1H), 4.18–4.12 (m, 1H), 4.02 (t, *J* = 4.8 Hz, 1H), 3.76–3.72 (m, 1H), 3.70 (s, 3H), 3.50 (ddd, *J* = 8.0, 7.5, 6.0 Hz, 1H), 3.28 (dd, *J* = 8.3, 11.8 Hz, 1H), 1.93–1.85 (m, 1H), 1.73–1.65 (m, 1H), 1.44–1.36 (m, 2H), 0.96 (t, *J* = 7.5 Hz). ^13^C NMR (100.6 MHz, 26 °C, CDCl_3_) δ 156.4/155.4 (C), 133.9 (C), 131.6 (CH), 129.2 (2 × CH), 127.5 (2 × CH), 80.4/79.6 (CH), 65.1/64.6 (CH), 52.4/51.9 (CH_3_), 51.0 (CH_2_), 34.9/34.4 (CH), 29.7 (CH_2_), 18.6 (CH_2_), 14.0 (CH_3_). MS *m/z* (rel intensity) 295 (M^+^, 13), 186 ([M – SPh]^+^, 100). HRMS (EI) [M]^+^ calcd for C_15_H_21_NO_3_S, 295.1242; found, 295.1237; [M – SPh]^+^ calcd for C_9_H_16_NO_3_, 186.1130; found, 186.1125. Anal. Calcd for C_15_H_21_NO_3_S: C, 60.99; H, 7.17; N, 4.74; S, 10.85. Found: C, 61.39; H, 7.21; N, 4.66; S, 10.99.

## Supplementary Information

Below is the link to the electronic supplementary material.

Supplementary file1 (PDF 3413 kb)

## Abbreviations

DIB: (Diacetoxyiodo)benzene
DCM: Dichloromethane
Hyp: Hydroxyproline
MeOH: Methanol
EtOAc: Ethyl acetate
