# Supplementary material for: Structural diversity using amino acid “Customizable Units”: conversion of hydroxyproline (Hyp) into nitrogen heterocycles
Source: Amino Acids. 2022 Apr 12;54(6):955–66. doi: 10.1007/s00726-022-03159-z (PMC9213323; doi:10.1007/s00726-022-03159-z)
Supplement: Supplementary file 1 — Supplementary file1 (PDF 3413 kb) [file 726_2022_3159_MOESM1_ESM.pdf]

Supporting Information

Structural Diversity by Using Amino Acid "Customizable Units": Conversion of Hydroxyproline (Hyp) into Nitrogen Heterocycles

Dácil Hernández,*[,1] Marina Porras,[1] Alicia Boto*[,1]

[1]Instituto de Productos Naturales y Agrobiología del CSIC, Avda. Astrofísico Francisco Sánchez 3, 38206-La Laguna, Tenerife, Spain. E-mail: alicia@ipna.csic.es, dacil@ipna.csic.es

Synthesis and characterization of scission-allylation substrates, acids **23-25**

Synthesis of the precursors of (-)-norconiine methyl carbamate **28a,b** and **28**

^{1}H and ^{13}C NMR spectra of compounds **9**, **11-22**, and **28-32**

HSQC of compounds **13**, **14**, **16**, **18** and **19**

Synthesis of scission-allylation substrates 23-25. Acid substrates **23** and **24** were prepared using classical methodologies as described below. Compound **25** was known and its synthesis followed the reported protocol (Hernández et al, 2021).

Preparation of Methyl (2*S*,4*R*)-4-trityloxy-N-methoxycarbonyl-L-proline (23a), methyl ester precursor of acid 23. A solution of commercial *N*-methoxycarbonyl-4*R*-hydroxy-L-proline methyl ester (260.0 mg, 1.3 mmol) in dry pyridine (1.5 mL) was treated with trityl chloride (714.0 mg, 2.6 mmol) and 4-dimethylaminopyridine (50.0 mg) and the reaction mixture was stirred at 80 °C for 72 h. Then it was cooled to room temperature, poured into 5% aq. HCl and extracted with EtOAc. The organic layer was dried and evaporated as usual, and the crude residue was purified by column chromatography on silica gel (hexane/EtOAc 80:20), yielding compound **23a** (543.0 mg, 95%) as viscous oil. $[\alpha]_D^{20}$ = –6 (c 0.88, $CHCl_3$); IR ($CHCl_3$) ν_{max}: 1745, 1697, 1452, 1394, 1091 cm^{-1}. ^1H NMR (500 MHz, 70 °C, CD_3OD). Rotamer equilibrium; two sets of signals at 26 °C, one set at 70 °C: δ 7.42 (d, *J* = 8.0 Hz, 6H), 7.29 (dd, *J* = 8.0, 7.0 Hz, 6H), 7.23 (dd, *J* = 7.5, 7.0 Hz, 3H), 4.34 (dd, *J* = 8.5, 5.0 Hz, 1H), 4.28–4.23 (m, 2H), 3.60 (s, 6H), 3.23–3.15 (m, 1H), 3.16–3.08 (m, 1H), 2.02 (ddd, *J* = 13.5, 8.8, 6.0 Hz, 1H), 1.59 (dt, *J* = 13.0, 5.3 Hz, 1H). ^{13}C NMR (125.7 MHz, 26 °C, CD_3OD): δ 174.4/174.3 (C), 157.3/156.6 (C), 145.6 (3 × C), 129.8 (6 × CH), 129.0 (6 × CH), 128.4 (3 × CH), 88.8/88.7 (C), 73.4/72.6 (CH), 59.2/58.8 (CH), 53.3/52.9 (CH_2), 53.25 (CH_3), 52.7 (CH_3), 38.1/37.4 (CH_2). MS *m/z* (rel intensity) 386 ([M – CO_2Me]+, 3) , 243 ($[CPh_3]^+$, 100). HRMS (EI) [M – CO_2Me]+, calcd for $C_{25}H_{24}NO_3$, 386.1756; found, 386.1759; $[CPh_3]$+, calcd for $C_{19}H_{15}$, 243.1174; found, 243.1178. Anal. Calcd for $C_{27}H_{27}NO_5$: C, 72.79; H, 6.11; N, 3.14. Found: C, 72.59; H, 6.33; N, 3.16.

Preparation of (2*S*,4*R*)-4-Trityloxy-*N*-methoxycarbonyl-L-proline (23). To an 1M NaOH solution in 9:1 MeOH:H_2O (5 mL) was added the methyl ester **23a** (543.0 mg, 1.2 mmol). The reaction mixture was stirred at 0 °C for 3 h. Then 5% aqueous HCl was added until pH = 2 and the mixture was extracted with EtOAc. The organic layer was dried and evaporated as before, affording the acid **23** (509.0 mg, 97%) as a viscous oil: $[\alpha]_D^{20}$ = –9 (c 0.62, $CHCl_3$); IR ($CHCl_3$) ν_{max} 1760, 1703, 1697, 1455, 1394, 1089 cm^{-1}. ^1H NMR (500 MHz, 70 °C, CD_3OD). Rotamer equilibrium; two sets of signals at 26 °C, one set at 70 °C: δ 7.42 (d, *J* = 7.5 Hz, 6H), 7.29 (dd, *J* = 7.5, 7.0 Hz, 6H), 7.23 (dd, *J* = 7.5, 7.0 Hz, 3H), 4.35–4.25 (m, 2H), 3.61 (s, 3H), 3.22–3.15 (m, 1H), 3.15–3.08 (m, 1H), 2.06 (ddd, *J* = 13.0, 9.0, 6.5 Hz, 1H), 1.67 (dt, *J* = 13.0, 5.3 Hz, 1H). ^{13}C NMR (125.7 MHz, 26 °C, CD_3OD): δ 175.8/175.7 (C), 157.3/156.8 (C), 145.7 (3 × C), 129.8 (6 × CH), 129.0 (6 × CH), 128.4 (3 × CH), 88.7 (C), 73.4/72.6 (CH), 59.1/58.9 (CH), 53.3/52.9 (CH_2), 53.2 (CH_3), 38.2/37.5 (CH_2). HRMS (ESI) *m/z* [M]+ calcd for $C_{26}H_{25}NO_5$ 431.1733; found, 431.1702. Anal. Calcd for $C_{26}H_{25}NO_5$: C, 72.37; H, 5.84; N, 3.25. Found: C, 72.22; H, 6.09; N, 3.20.

Preparation of (2*S*,4*S*)-4-Trityloxy-*N*-methoxycarbonyl-L-proline (24). The precursor (2*S*,4*S*)-4-Hydroxy-*N*-methoxycarbonyl-L-proline methyl ester is known and was prepared as previously reported (Hernández et al, 2021). A solution of the precursor (520.0 mg, 2.6 mmol) in dry pyridine (3 mL) was treated with trityl chloride (1430.0 mg, 5.1 mmol) and 4-dimethylaminopyridine (100.0 mg) and the reaction mixture was stirred at 80 °C for 72 h. Then it was cooled to room temperature, poured into 5% aq. HCl and extracted with EtOAc. The organic layer was dried and evaporated as usual, and the crude was dissolved in 1M NaOH in 9:1 MeOH:H_2O (5 mL). After stirring at 0 °C for 7 h, 5% aq. HCl was added until pH = 2, and the mixture was extracted with EtOAc. The organic layer was dried and evaporated as before, and the residue was purified by column chromatography (CH_2Cl_2/MeOD 95:5) affording acid **24** (837.0 mg, 76%) as a viscous oil: $[\alpha]_D^{20}$ = –43 (c 0.65, $CHCl_3$); IR ($CHCl_3$) ν_{max}: 1737, 1696, 1450, 1395, 1082 cm^{-1}. ^1H NMR (500 MHz, 70 °C, CD_3OD). Rotamer equilibrium; two sets of signals visible at 26 °C, one set at 70 °C: δ 7.45–7.43 (m, 6H), 7.30–7.27 (m, 6H), 7.24–7.20 (m, 3H), 4.21 (dq, *J* = 6.0, 5.3 Hz, 1H), 4.16–4.10 (m, 1H), 3.59 (s, 3H), 3.23–3.16 (m, 1H), 3.16–3.08 (m, 1H), 2.06–1.98 (m, 1H), 1.82 (dt, *J* = 13.0, 4.7 Hz, 1H). ^{13}C NMR (100.6 MHz, 26 °C, CD_3OD): δ 176.0 (C), 157.3/157.0 (C), 145.8 (3 × C), 129.9/129.8 (6 × CH), 129.0/128.7 (6 × CH), 128.3/128.0 (3 × CH), 89.0 (C), 73.7/72.9 (CH), 59.4 (CH), 54.0/53.7 (CH_2), 53.1/53.0 (CH_3), 38.4/37.6 (CH_2). HRMS (ESI-TOF) *m/z* calcd for $C_{26}H_{25}NO_5Na$ [M + Na]+, 454.1630; found, 454.1632. Anal. Calcd for $C_{26}H_{25}NO_5$: C, 72.37; H, 5.84; N, 3.25. Found: C, 72.55; H, 5.59; N, 2.95.

Synthesis of the precursors of the methyl carbamate of (-)-norconiine 28a, 28b and 28.

(2*R*,4*S*)-2-(Propyl)-4-(hydroxy)-*N*-(methoxycarbonyl) pyrrolidine (28a). A solution of the 4-hydroxypyrrolidine **27** (556.0 mg, 3.0 mmol) in MeOH (20 mL) was treated with 10% Pd/C (160 mg) and stirred overnight under hydrogen atmosphere (1 atm). The mixture was filtered through a Celite column, and the filtrate was evaporated to give the reduced product **28a** in quantitative yield (560.0 mg, 3.0 mmol, 99%). Compound **28a** is known (Hernández et al, 2021).

(2*R*,4*S*)-4-(Phenylselanyl)-2-(propyl)-*N*-(methoxy- carbonyl)pyrrolidine (28b). Compound **28a** (560.0 mg, 3.0 mmol) was dissolved in dry THF (6 mL) and the solution was stored for the next step. Meanwhile, methyl triflate (0.5 mL, 4.5 mmol) was slowly added to a solution of 1-(p-toluensulfonyl)imidazole (1020.0 mg, 4.5 mmol) in dry THF (6 mL), at 0 °C and under nitrogen atmosphere. The mixture was stirred for 0.5 h and then the stored solution of the 4-hydroxypyrrolidine in THF and *N*-methylimidazole (369.0 mg, 360 µL, 4.5 equiv) were added. The mixture was stirred at room temperature overnight, then poured into water and extracted with EtOAc. The in dry dichloroethane organic extract was dried over anhydrous Na_2SO_4 and concentrated under vacuum. The residue was quickly purified by chromatography on silica gel (hexanes/ethyl acetate 85:15) to give an intermediate tosylate (820.0 mg) as a viscous oil (HRMS (ESI) *m/z* calcd for $C_{16}H_{23}NO_5SNa$ $[M + Na]^+$ 364.11.95; found, 364.1191). The tosylate was dissolved in *tert*-butanol (12 mL) and treated with sodium phenylselenide, which was formed beforehand from diphenyl selenide (493.0 mg, 1.6 mmol) and sodium borohydride (114.0 mg, 3.0 mmol) in *tert*-butanol (12 mL) under reflux until the disappearance of the yellow color. After addition of the tosylate, the mixture was stirred under reflux for 2.5 h. Then it was cooled to room temperature, poured into water and extracted with EtOAc. The organic extract was dried and evaporated as usual, and the residue was purified by chromatography on silica gel (hexane/EtOAc 80:20), affording the selenide **28b** (598.0 mg, 61%) as a viscous oil. $[\alpha]_D^{20}$= –15 (c 0.47, $CHCl_3$). IR ($CHCl_3$) ν_{max}: 1686, 1454, 1387, 1131, 1099 cm^{-1}. ^{1}H NMR (500 MHz, 70 °C, $CDCl_3$) Rotamer equilibrium; two sets of signals at 26 °C, one set at 70 °C: δ 7.53 (br d, *J* = 8.0 Hz, 2H), 7.27–7.23 (m, 3H), 3.94–3.88 (m, 1H), 3.72–3.67 (m, 2H), 3.65 (s, 3H), 3.56–3.49 (m, 1H), 2.05–2.02 (m, 2H), 1.80–1.68 (m, 1H), 1.36–1.24 (m, 3H), 0.89 (t, *J* = 7.0 Hz, 3H). ^{13}C NMR (125.7 MHz, 70 °C, $CDCl_3$) δ 155.5 (C), 134.9 (2 × CH), 129.2 (2 × CH), 128.8 (C), 127.9 (CH), 57.5 (CH), 53.3 (CH_2), 52.1 (CH_3), 38.1 (CH), 37.7 (CH_2), 36.8 (CH_2), 19.3 (CH_2), 13.9 (CH_3). HRMS (ESI) *m/z* $[M + Na]^+$ calcd for $C_{15}H_{21}NO_2SeNa$, 350.0635; found, 350.0636. Anal. Calcd for $C_{15}H_{21}NO_2Se$: C, 55.21; H, 6.49; N, 4.29. Found: C, 55.34; H, 6.28; N, 4.05.

(2*R*)-2-(Propyl)-*N*-(methoxycarbonyl)-2,5-dihydro-1*H*-pyrrole (28). The seleno derivative **28b** (654.0 mg, 2.0 mmol) was dissolved in dry pyridine (220 µL, 213.0 mg, 2.7 mmol) and 33% hydrogen peroxide (aqueous solution, 30% w/w, 230 µL, 2.2 mmol) was added at 0 °C. The mixture was stirred for 1.5 h, then poured into aqueous $NaHCO_3$ and extracted with CH_2Cl_2. The organic extract was dried over anhydrous Na_2SO_4 and concentrated under vacuum. The residue was purified by chromatography on silica gel (hexane/EtOAc 95:5) to give the dihydropyrrole **28** (286.0 mg, 85%) as a viscous oil. $[\alpha]_D^{20}$= –70 (c 0.10, $CHCl_3$). IR ($CHCl_3$) ν_{max}: 1689, 1455, 1391, 1127, 1112 cm^{-1}. ^{1}H NMR (500 MHz, 70 °C, $CDCl_3$) Rotamer equilibrium; two sets of signals at 26 °C, one set at 70 °C: δ 5.75 (s, 2H), 4.63–4.54 (m, 1H), 4.27–4.17 (m, 1H), 4.04 (dd, *J* = 15.5, 5.0 Hz, 1H), 3.71 (s, 3H), 1.80–1.68 (m, 1H), 1.68–1.60 (m, 1H), 1.30 (sextuplet, *J* = 7.5 Hz, 2H), 0.92 (t, *J* = 7.5 Hz, 3H). ^{13}C NMR (125.7 MHz, 70 °C, $CDCl_3$): δ 155.4 (C), 130.4 (CH), 124.8 (CH), 64.3 (CH), 53.7 (CH_2), 52.0 (CH_3), 36.2 (CH_2), 17.8 (CH_2), 14.0 (CH_3). HRMS (ESI) *m/z* $[M + Na]^+$ calcd for $C_9H_{15}NO_2Na$ 192.1000; found, 192.0997. Anal. Calcd for $C_9H_{15}NO_2$: C, 63.88; H, 8.93; N, 8.28. Found: C, 63.84; H, 8.66; N, 8.62.

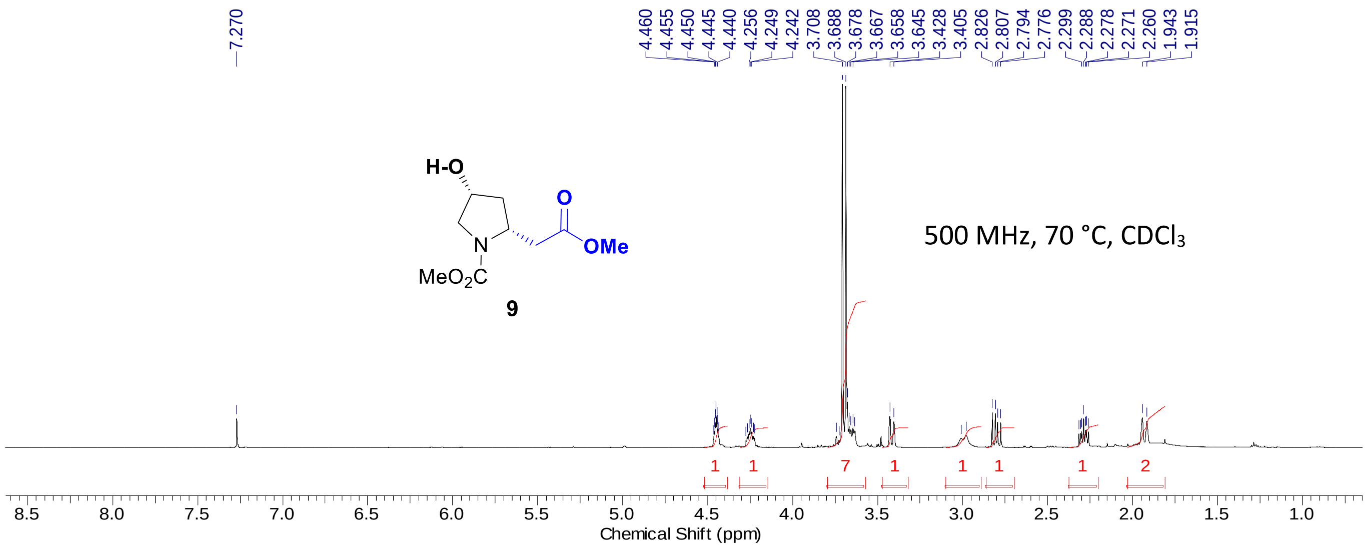
7.270
4.460 4.455 4.450 4.445 4.440 4.256 4.249 4.242 3.708 3.688 3.678 3.667 3.658 3.645 3.428 3.405 2.826 2.807 2.794 2.776 2.299 2.288 2.278 2.271 2.260 1.943 1.915
H-O
O
OMe
N
MeO2C
9
500 MHz, 70 °C, CDCl3
1 1 7 1 1 1 1 2
8.5 8.0 7.5 7.0 6.5 6.0 5.5 5.0 4.5 4.0 3.5 3.0 2.5 2.0 1.5 1.0
Chemical Shift (ppm)

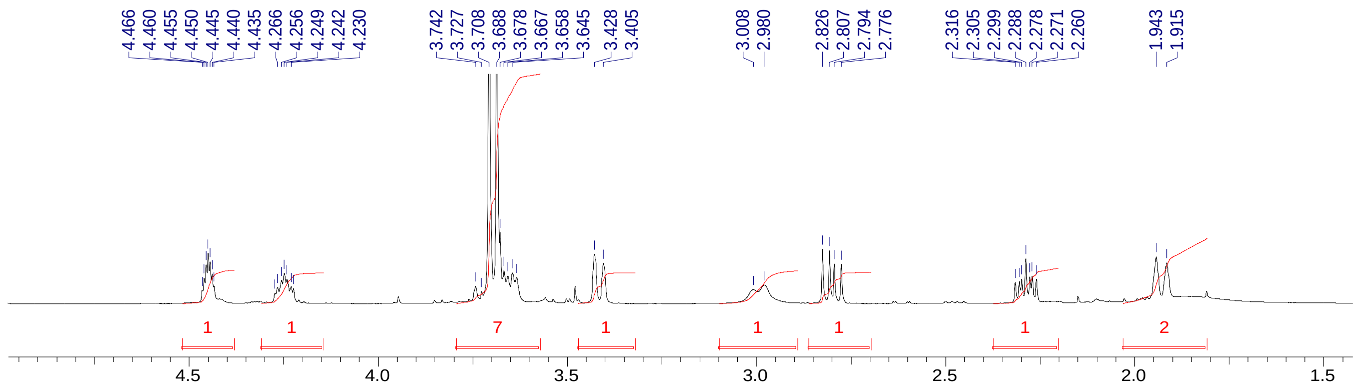
4.466 4.460 4.455 4.450 4.445 4.440 4.435 4.266 4.256 4.249 4.242 4.230 3.742 3.727 3.708 3.688 3.678 3.667 3.658 3.645 3.428 3.405 3.008 2.980 2.826 2.807 2.794 2.776 2.316 2.305 2.299 2.288 2.278 2.271 2.260 1.943 1.915
1 1 7 1 1 1 1 2
4.5 4.0 3.5 3.0 2.5 2.0 1.5

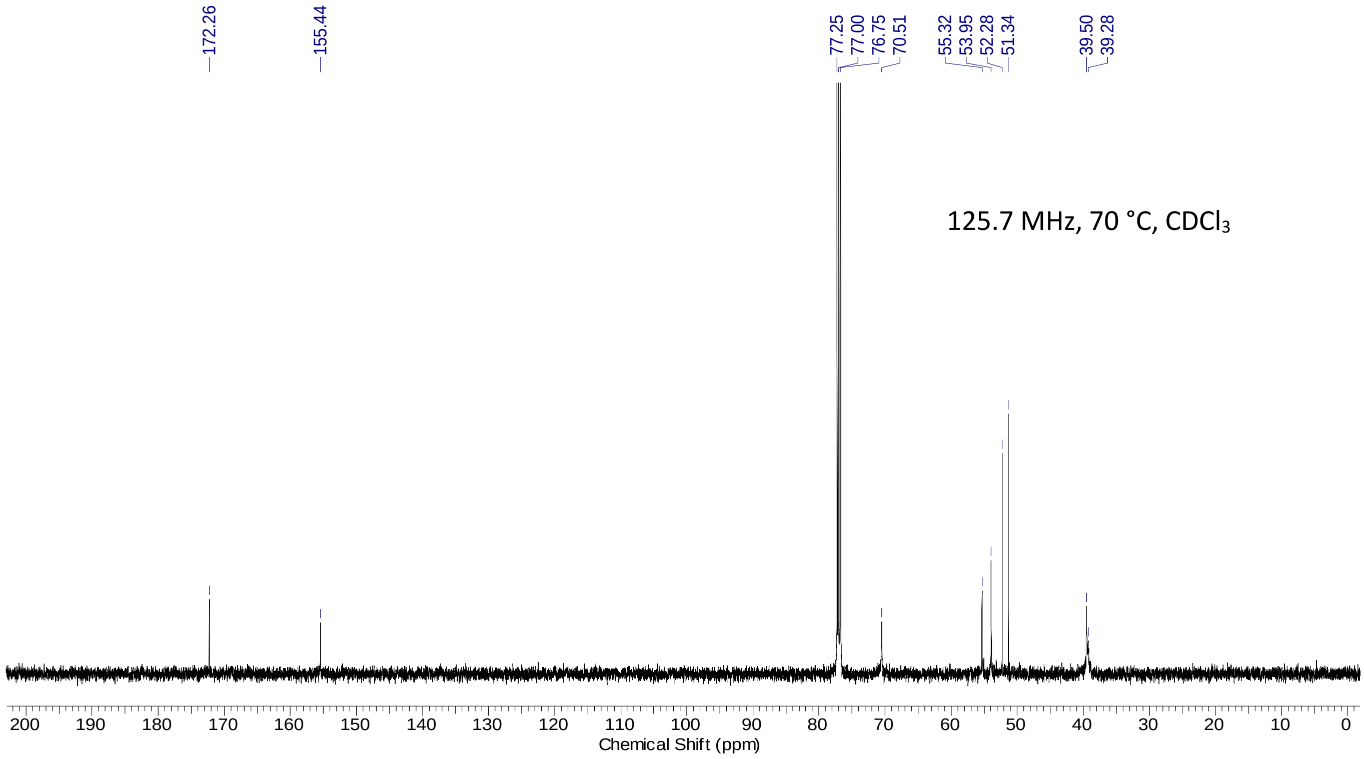
172.26 155.44 77.25 77.00 76.75 70.51 55.32 53.95 52.28 51.34 39.50 39.28
125.7 MHz, 70 °C, CDCl3
200 190 180 170 160 150 140 130 120 110 100 90 80 70 60 50 40 30 20 10 0
Chemical Shift (ppm)

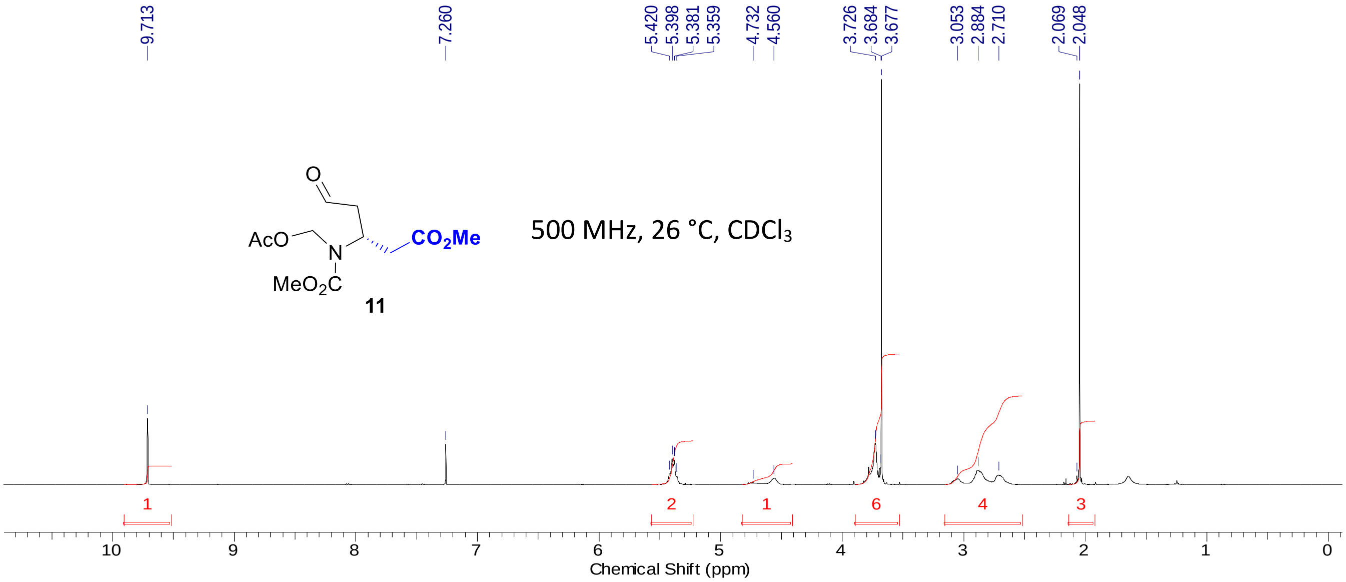
O
AcO
N
CO2Me
MeO2C
11
500 MHz, 26 °C, CDCl3
9.713
7.260
5.420
5.398
5.381
5.359
4.732
4.560
3.726
3.684
3.677
3.053
2.884
2.710
2.069
2.048
1
2
1
6
4
3
Chemical Shift (ppm)

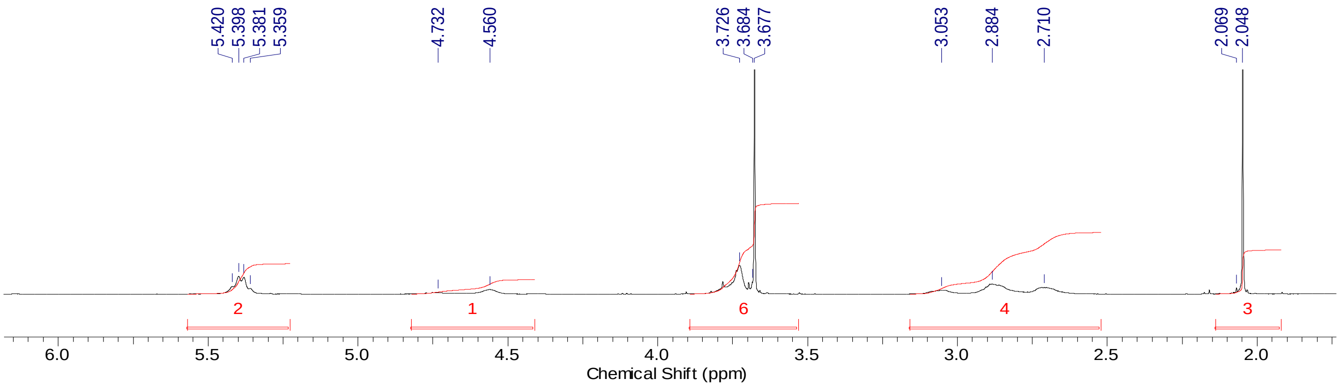
5.420
5.398
5.381
5.359
4.732
4.560
3.726
3.684
3.677
3.053
2.884
2.710
2.069
2.048
2
1
6
4
3
Chemical Shift (ppm)

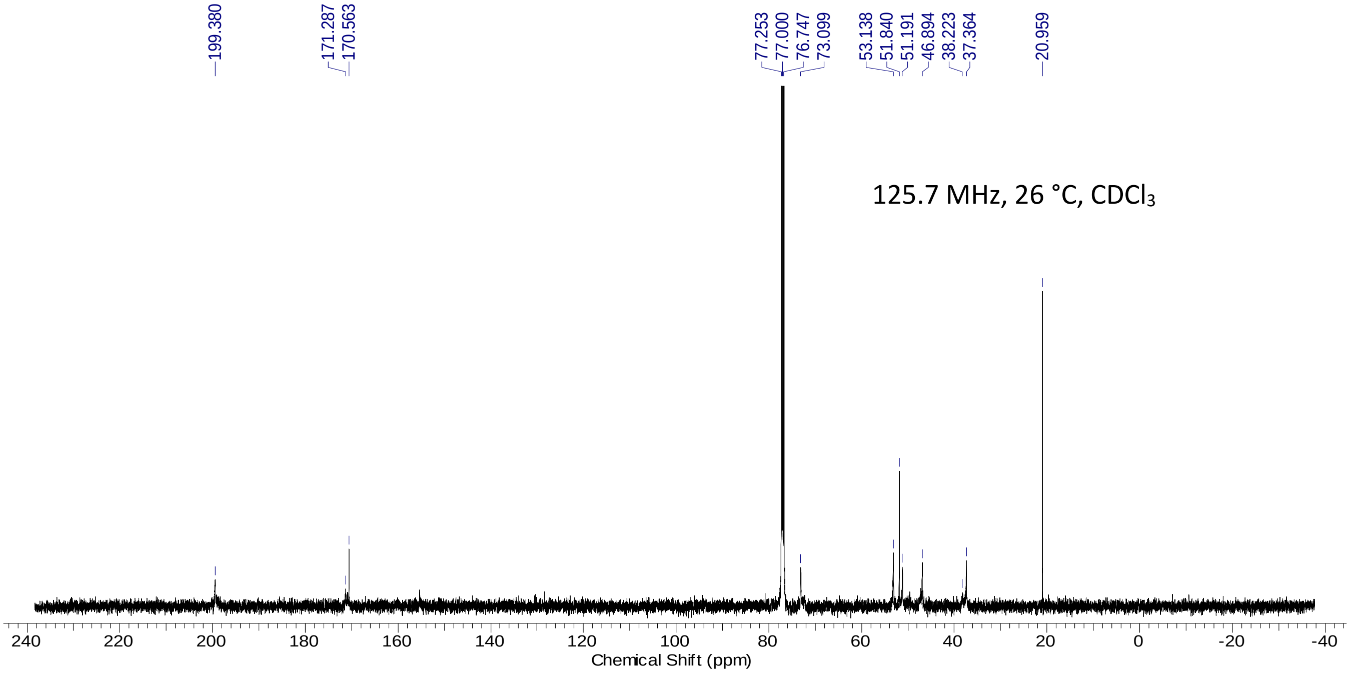
125.7 MHz, 26 °C, CDCl3
199.380
171.287
170.563
77.253
77.000
76.747
73.099
53.138
51.840
51.191
46.894
38.223
37.364
20.959
Chemical Shift (ppm)

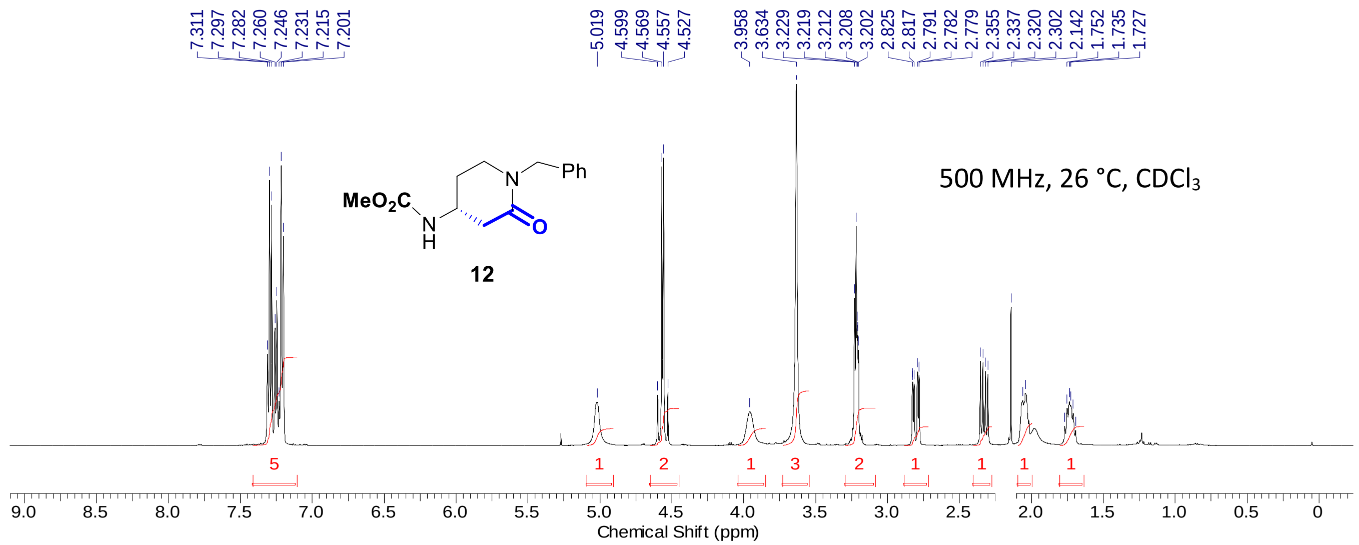
7.311 7.297 7.282 7.260 7.246 7.231 7.215 7.201
5.019 4.599 4.569 4.557 4.527
3.958 3.634 3.229 3.219 3.212 3.208 3.202 2.825 2.817 2.791 2.782 2.779 2.355 2.337 2.320 2.302 2.142 1.752 1.735 1.727
MeO2C
N
H
N
Ph
O
12
500 MHz, 26 °C, CDCl3
5 1 2 1 3 2 1 1 1 1
9.0 8.5 8.0 7.5 7.0 6.5 6.0 5.5 5.0 4.5 4.0 3.5 3.0 2.5 2.0 1.5 1.0 0.5 0
Chemical Shift (ppm)

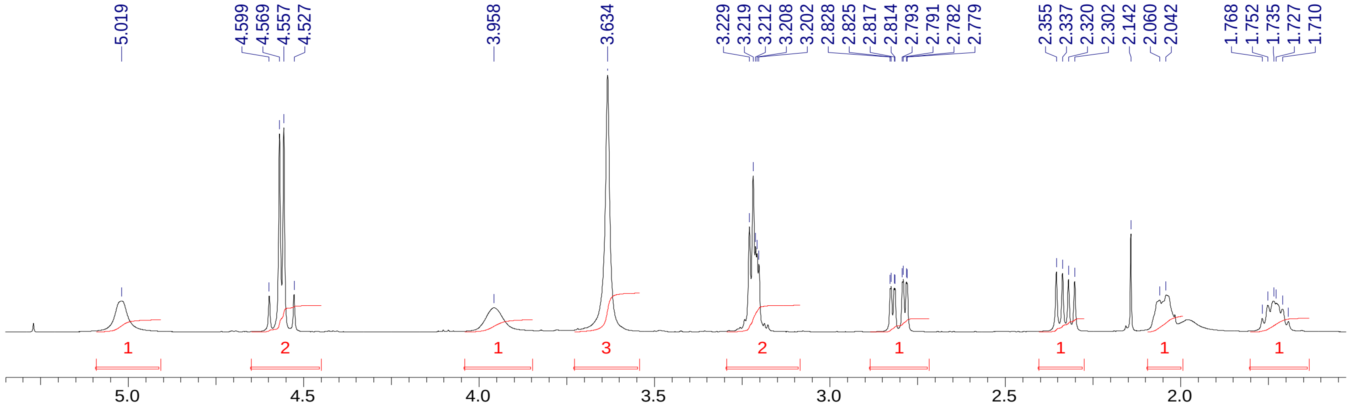
5.019
4.599 4.569 4.557 4.527
3.958
3.634
3.229 3.219 3.212 3.208 3.202 2.828 2.825 2.817 2.814 2.793 2.791 2.782 2.779
2.355 2.337 2.320 2.302 2.142 2.060 2.042
1.768 1.752 1.735 1.727 1.710
1 2 1 3 2 1 1 1 1
5.0 4.5 4.0 3.5 3.0 2.5 2.0

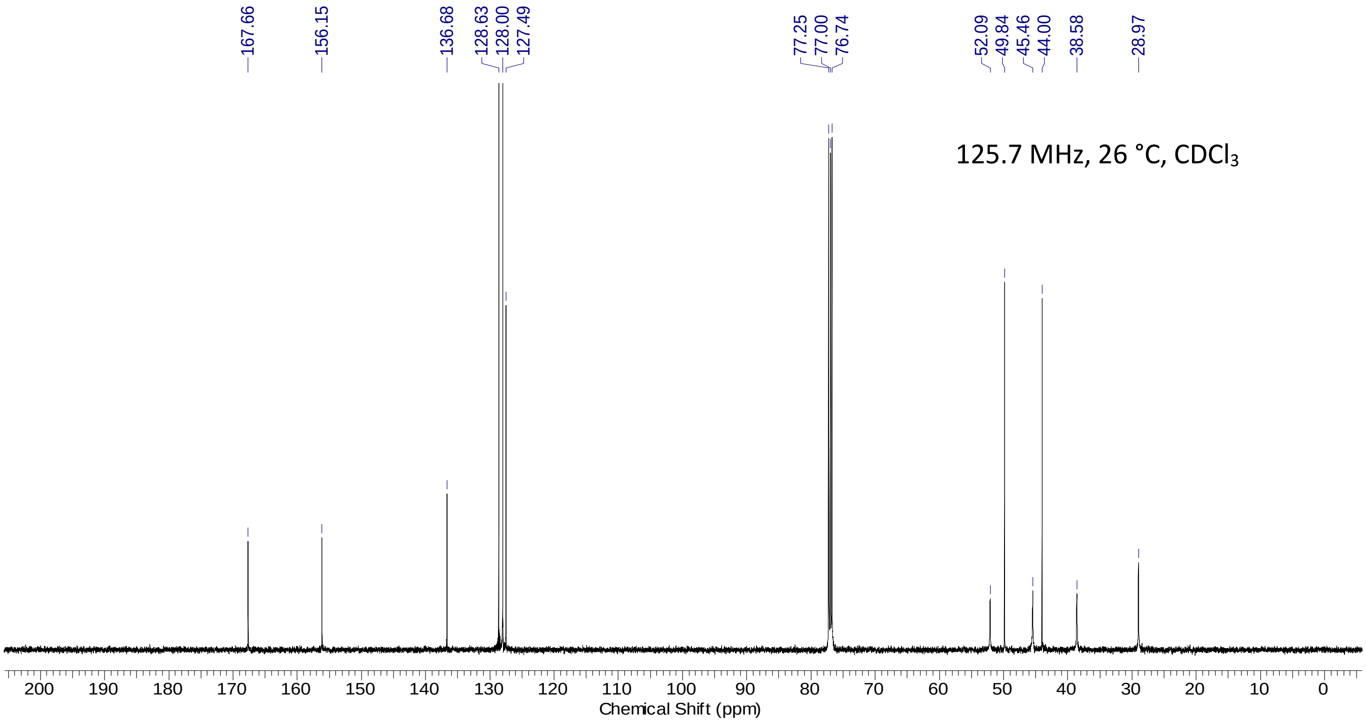
167.66
156.15
136.68 128.63 128.00 127.49
77.25 77.00 76.74
52.09 49.84 45.46 44.00 38.58
28.97
125.7 MHz, 26 °C, CDCl3
200 190 180 170 160 150 140 130 120 110 100 90 80 70 60 50 40 30 20 10 0
Chemical Shift (ppm)

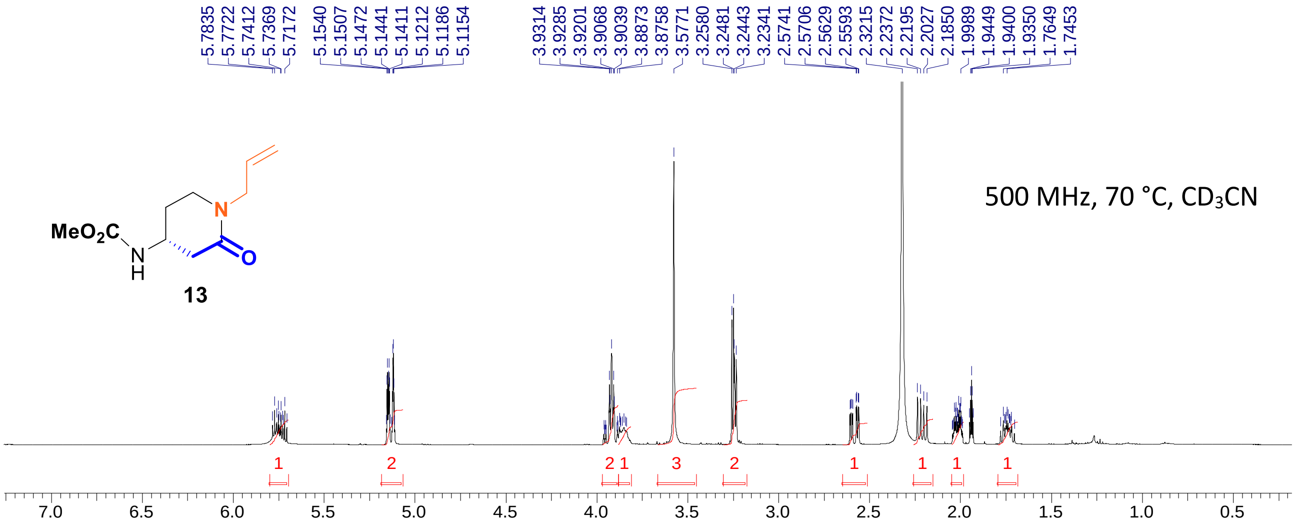
MeO$_2$C
N
H
N
O
500 MHz, 70 °C, CD$_3$CN

13

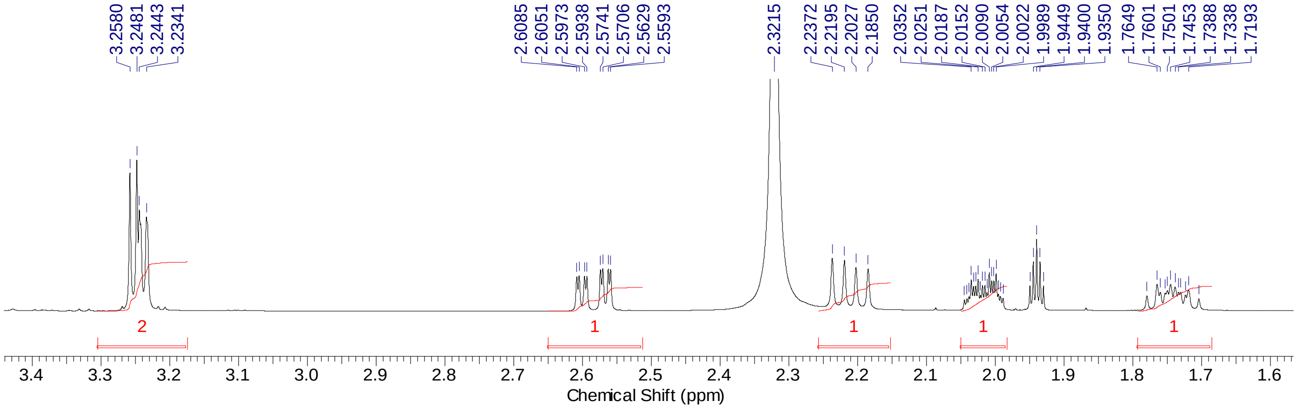
Chemical Shift (ppm)

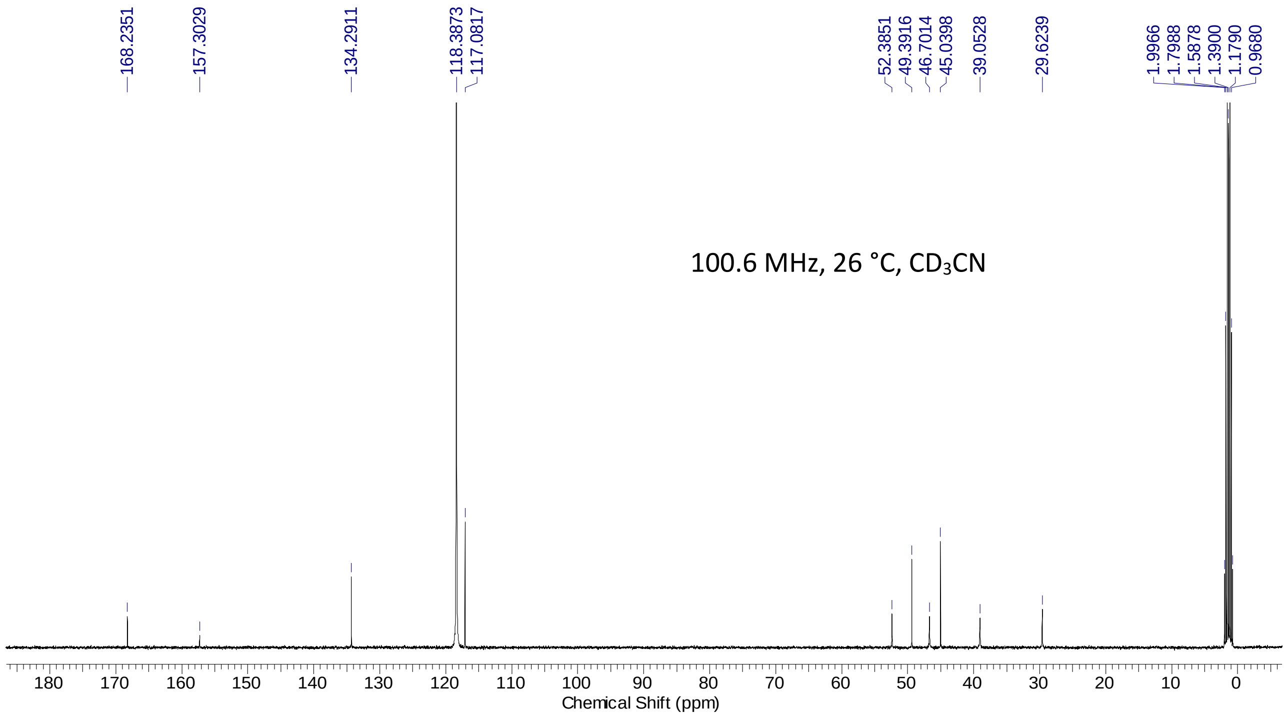
100.6 MHz, 26 °C, CD$_3$CN
Chemical Shift (ppm)

HSQC, 26 °C, CD_3CN

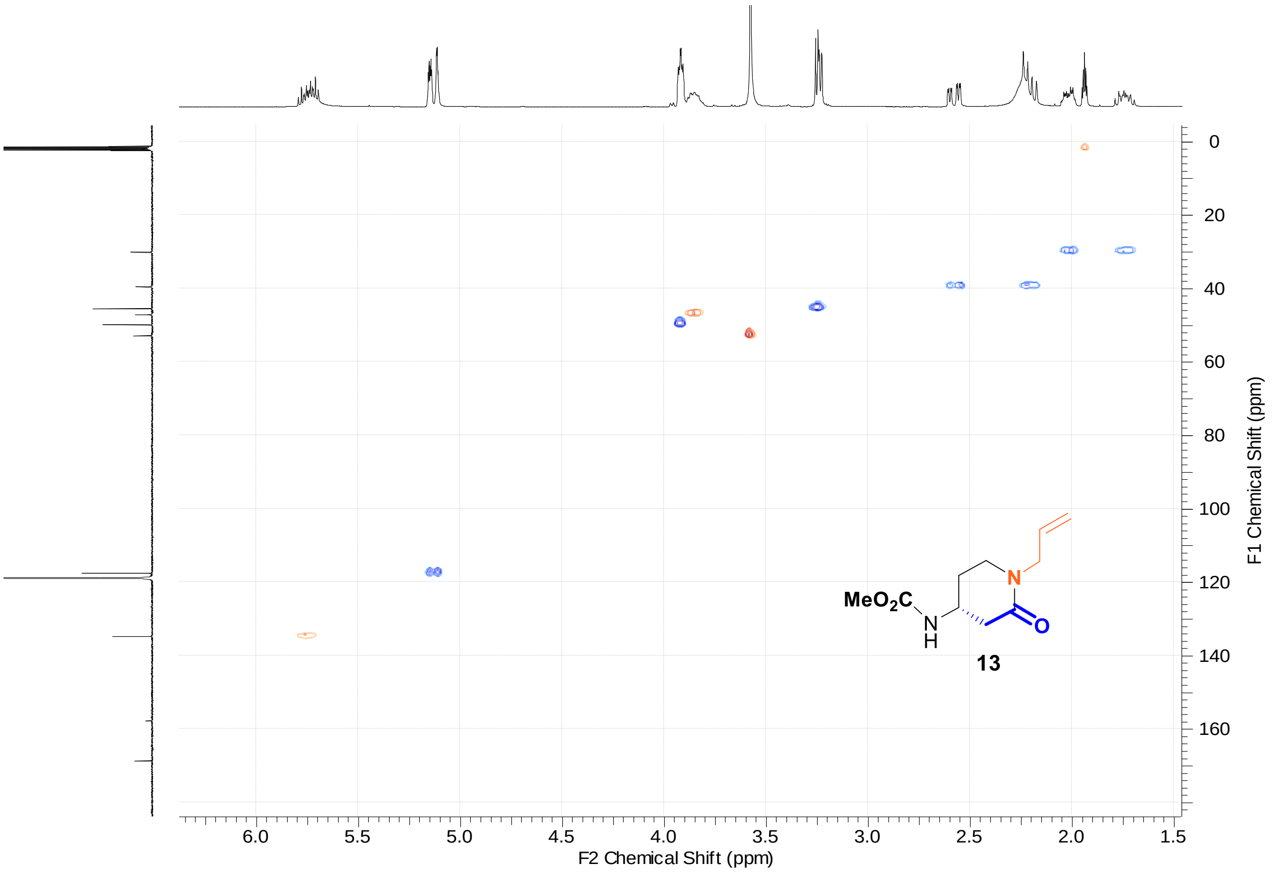

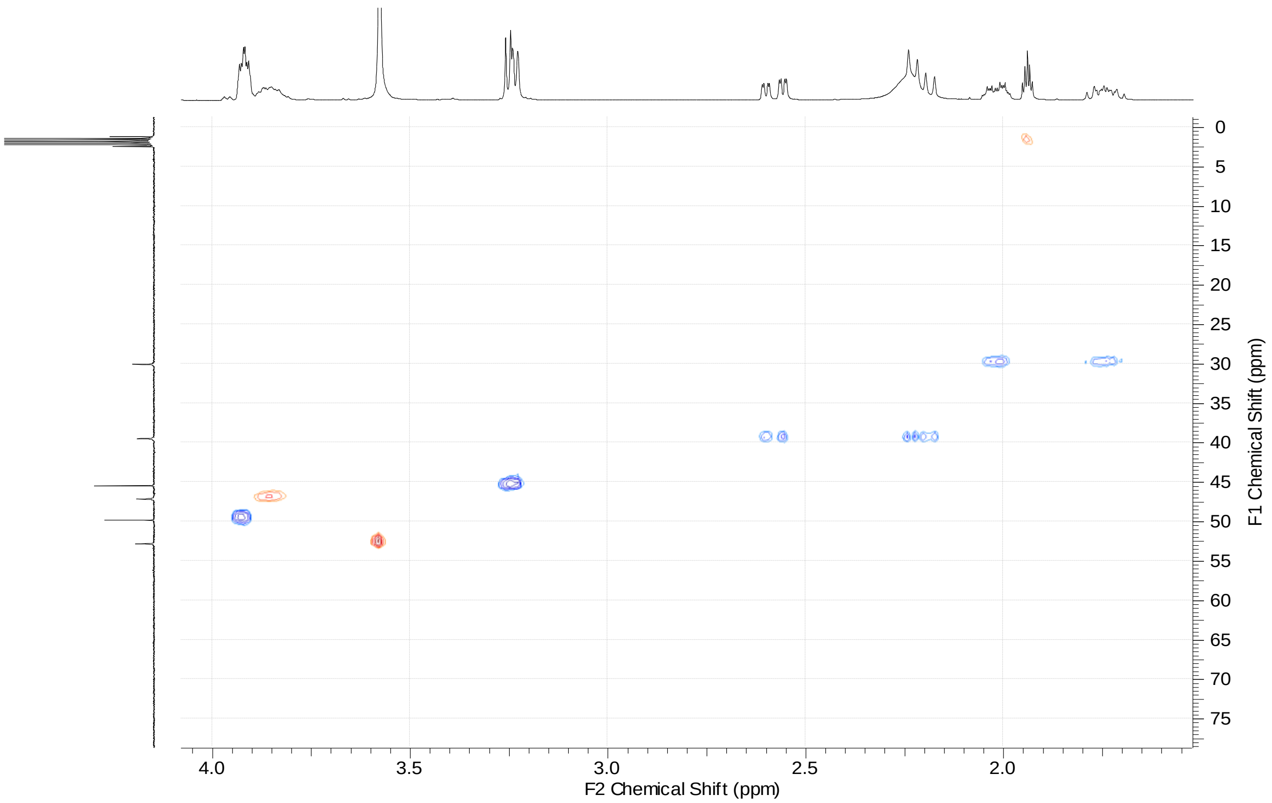

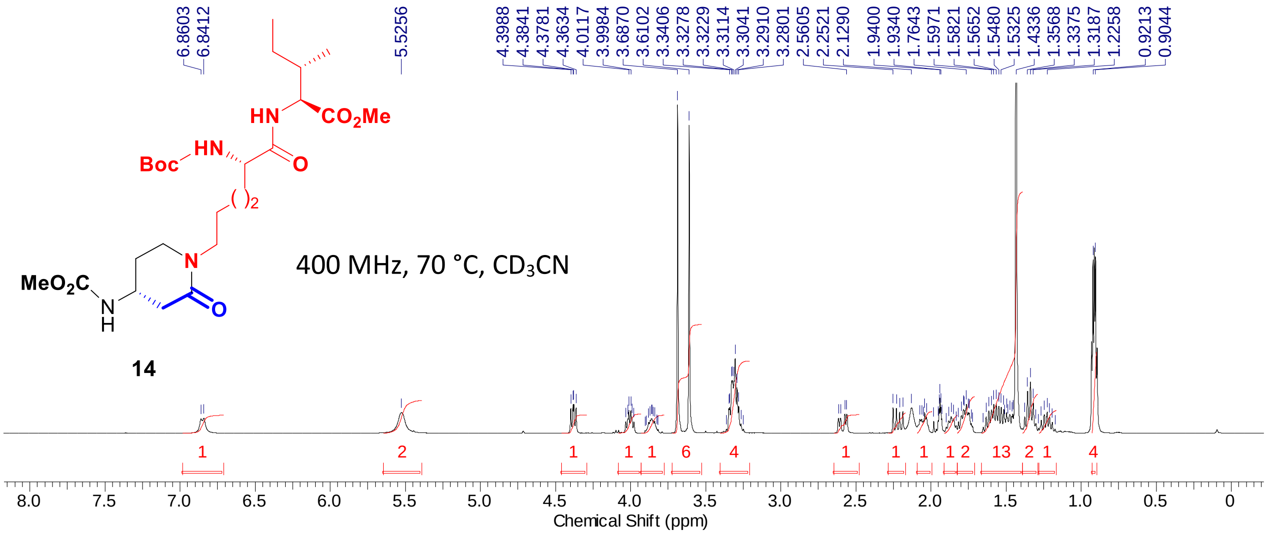

Boc
HN
HN
CO_2Me
O
( )2
N
MeO_2C
N
H
O
14
400 MHz, 70 °C, CD_3CN
6.8603
6.8412
5.5256
4.3988
4.3841
4.3781
4.3634
4.0117
3.9984
3.6870
3.6102
3.3406
3.3278
3.3229
3.3114
3.3041
3.2910
3.2801
2.5605
2.2521
2.1290
1.9400
1.9340
1.7643
1.5971
1.5821
1.5652
1.5480
1.5325
1.4336
1.3568
1.3375
1.3187
1.2258
0.9213
0.9044
1
2
1
1
1
6
4
1
1
1
1
2
13
2
1
4
Chemical Shift (ppm)

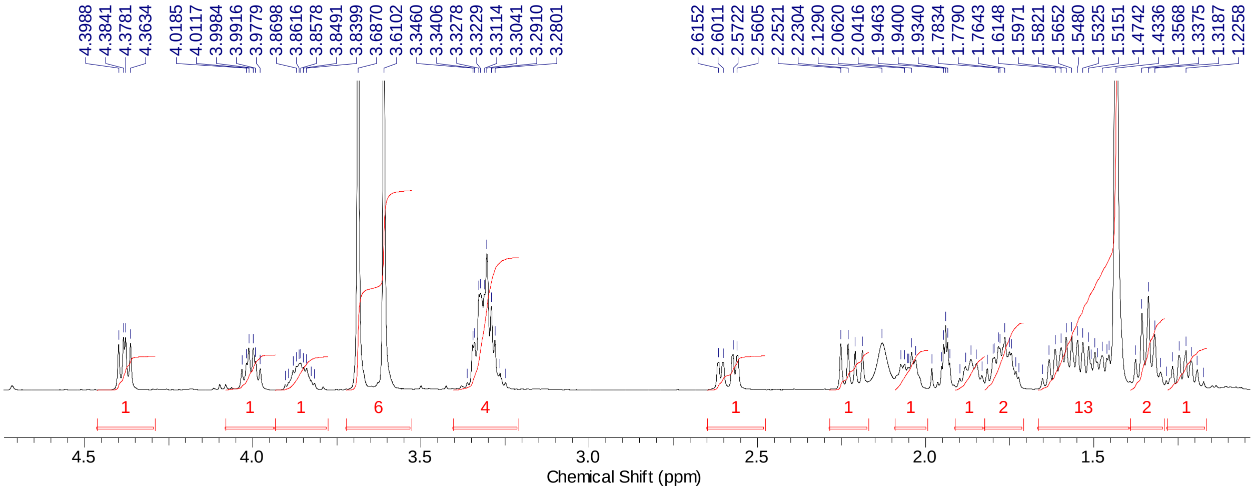

4.3988
4.3841
4.3781
4.3634
4.0185
4.0117
3.9984
3.9916
3.9779
3.8698
3.8616
3.8578
3.8491
3.8399
3.6870
3.6102
3.3460
3.3406
3.3278
3.3229
3.3114
3.3041
3.2910
3.2801
2.6152
2.6011
2.5722
2.5605
2.2521
2.2304
2.1290
2.0620
2.0416
1.9463
1.9400
1.9340
1.7834
1.7790
1.7643
1.6148
1.5971
1.5821
1.5652
1.5480
1.5325
1.5151
1.4742
1.4336
1.3568
1.3375
1.3187
1.2258
1
1
1
6
4
1
1
1
1
2
13
2
1
Chemical Shift (ppm)

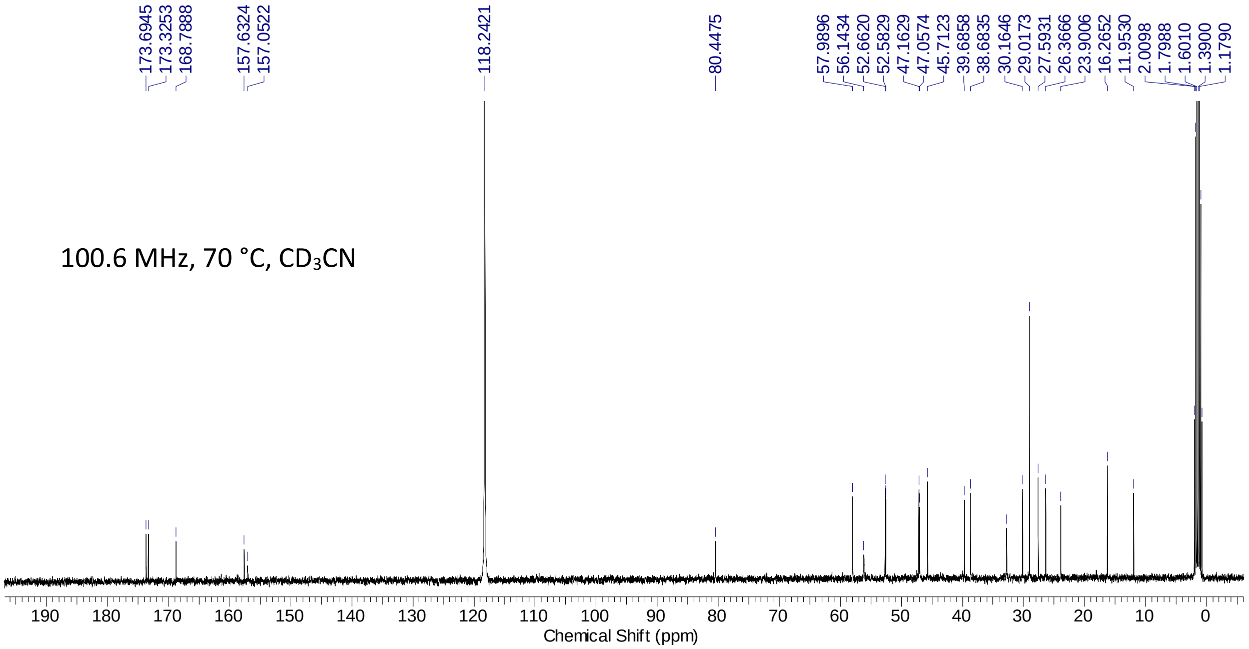

173.6945
173.3253
168.7888
157.6324
157.0522
118.2421
80.4475
57.9896
56.1434
52.6620
52.5829
47.1629
47.0574
45.7123
39.6858
38.6835
30.1646
29.0173
27.5931
26.3666
23.9006
16.2652
11.9530
2.0098
1.7988
1.6010
1.3900
1.1790
100.6 MHz, 70 °C, CD_3CN
Chemical Shift (ppm)

HSQC, 70 °C, CD_3CN

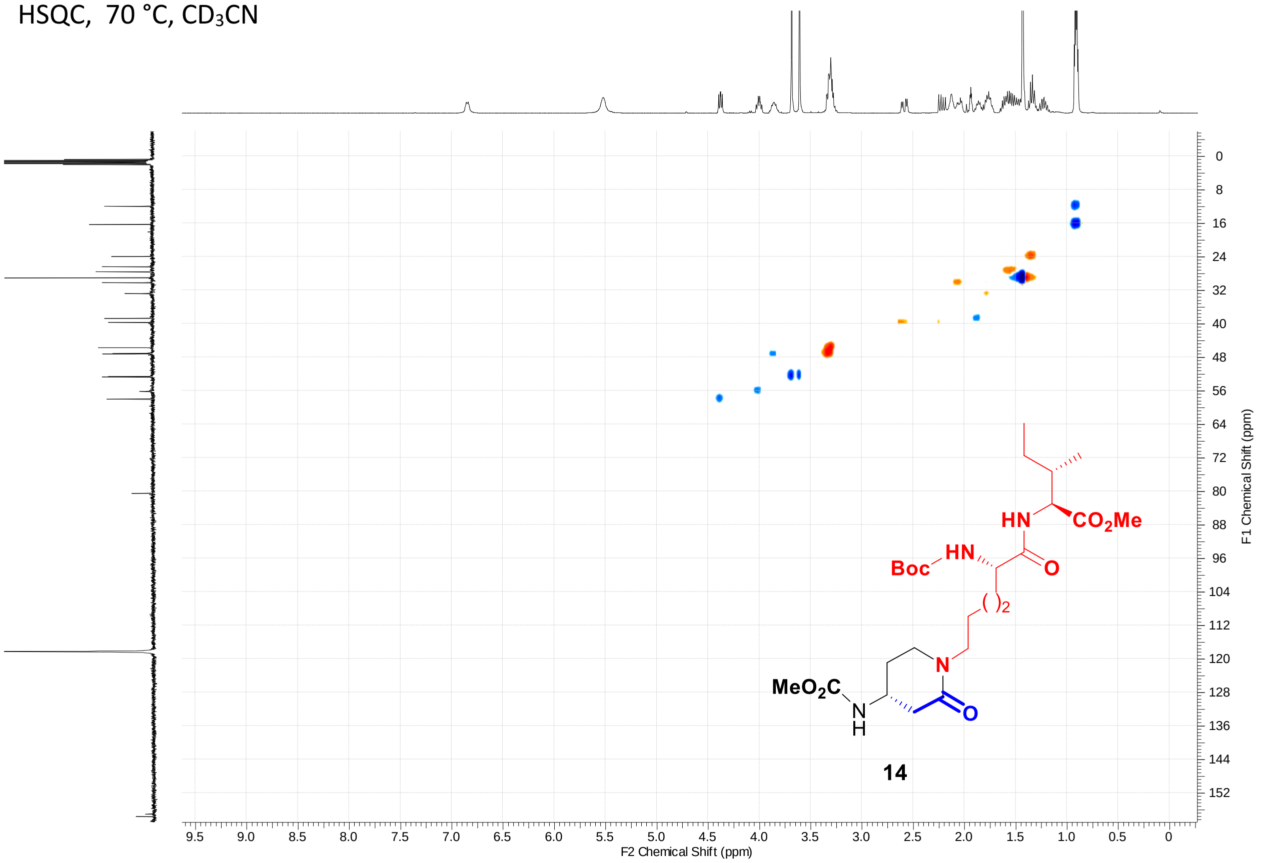

DA1-148-P-HSQC.2rr.esp

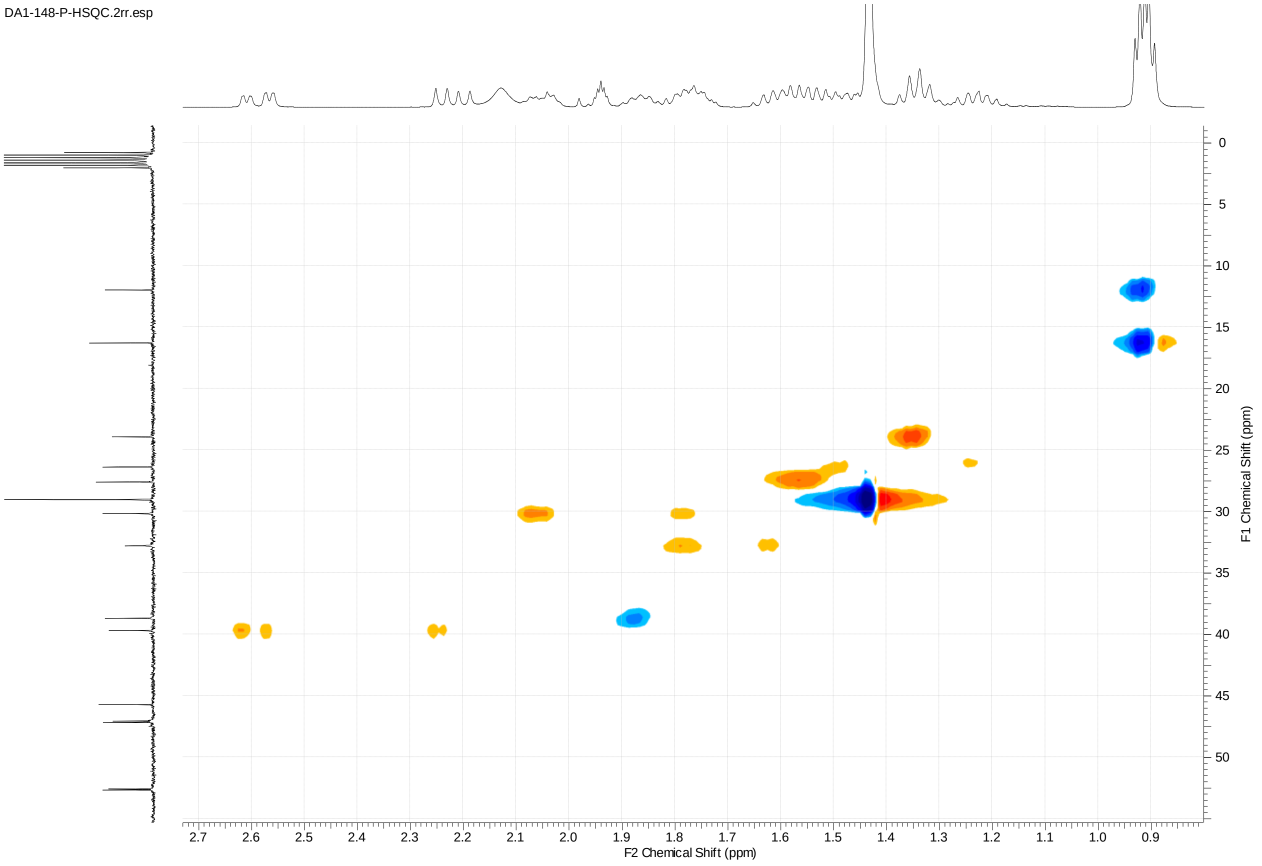

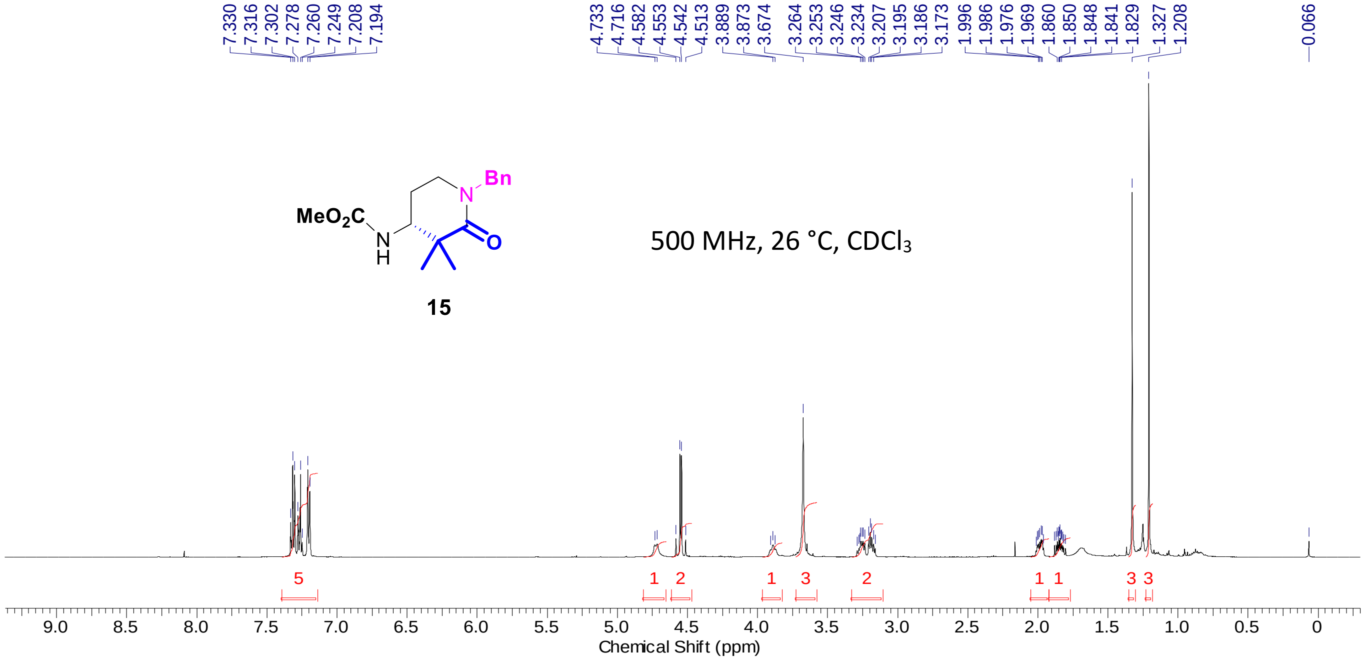
7.330
7.316
7.302
7.278
7.260
7.249
7.208
7.194
4.733
4.716
4.582
4.553
4.542
4.513
3.889
3.873
3.674
3.264
3.253
3.246
3.234
3.207
3.195
3.186
3.173
1.996
1.986
1.976
1.969
1.860
1.850
1.848
1.841
1.829
1.327
1.208
0.066
MeO2C
N
H
N
Bn
O
15
500 MHz, 26 °C, CDCl3
5
1
2
1
3
2
1
1
3
3
9.0 8.5 8.0 7.5 7.0 6.5 6.0 5.5 5.0 4.5 4.0 3.5 3.0 2.5 2.0 1.5 1.0 0.5 0
Chemical Shift (ppm)

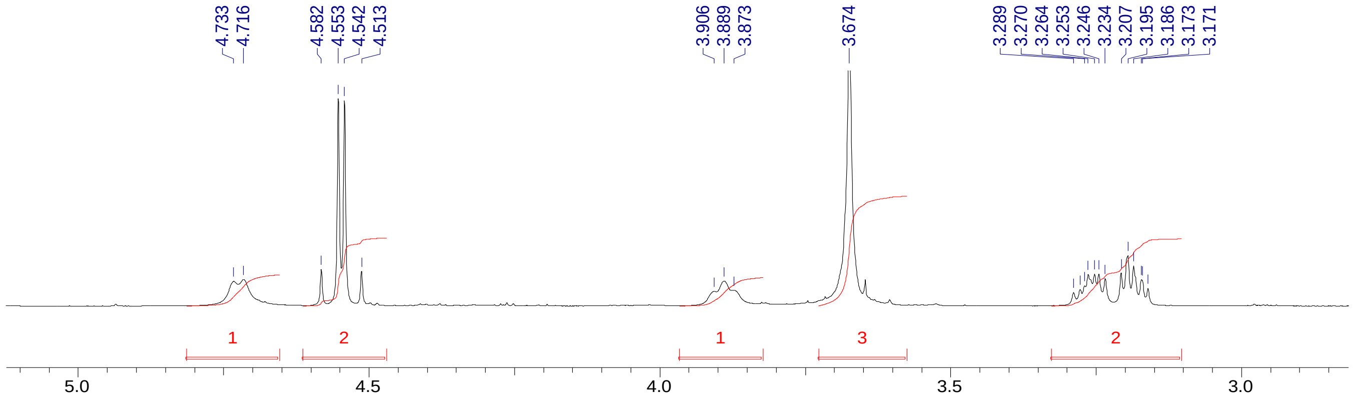
4.733
4.716
4.582
4.553
4.542
4.513
3.906
3.889
3.873
3.674
3.289
3.270
3.264
3.253
3.246
3.234
3.207
3.195
3.186
3.173
3.171
1
2
1
3
2
5.0 4.5 4.0 3.5 3.0

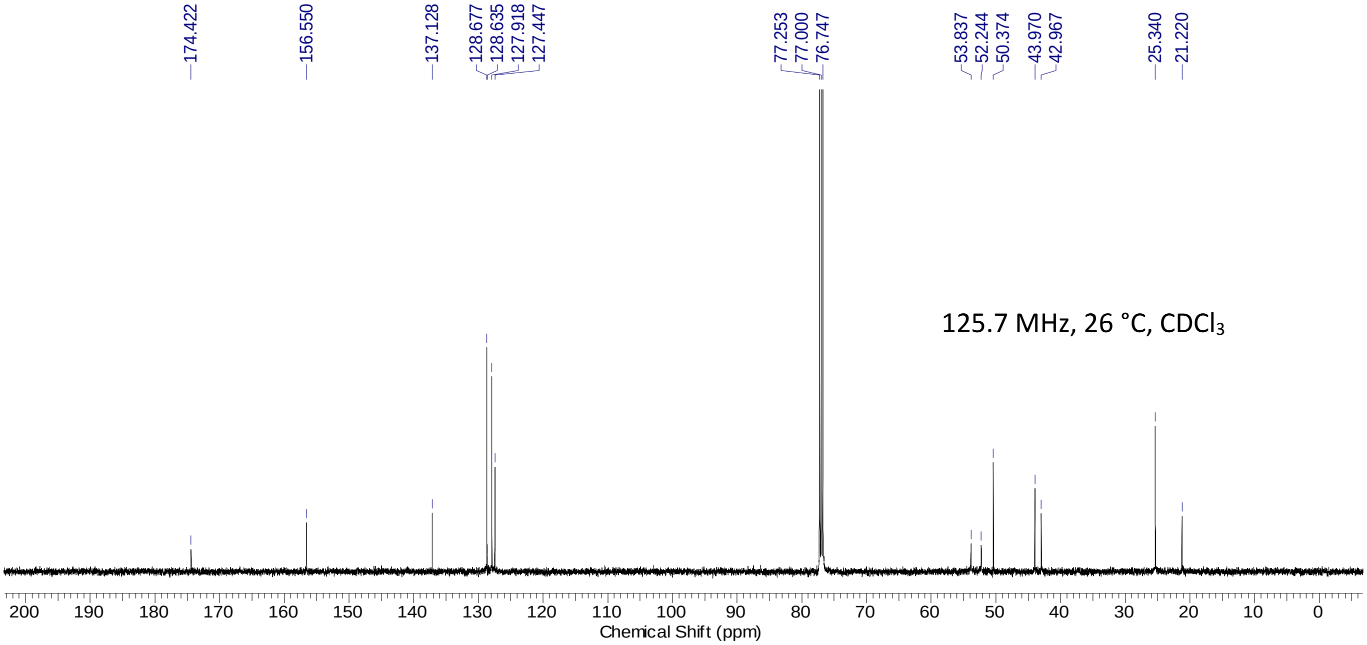
174.422
156.550
137.128
128.677
128.635
127.918
127.447
77.253
77.000
76.747
53.837
52.244
50.374
43.970
42.967
25.340
21.220
125.7 MHz, 26 °C, CDCl3
200 190 180 170 160 150 140 130 120 110 100 90 80 70 60 50 40 30 20 10 0
Chemical Shift (ppm)

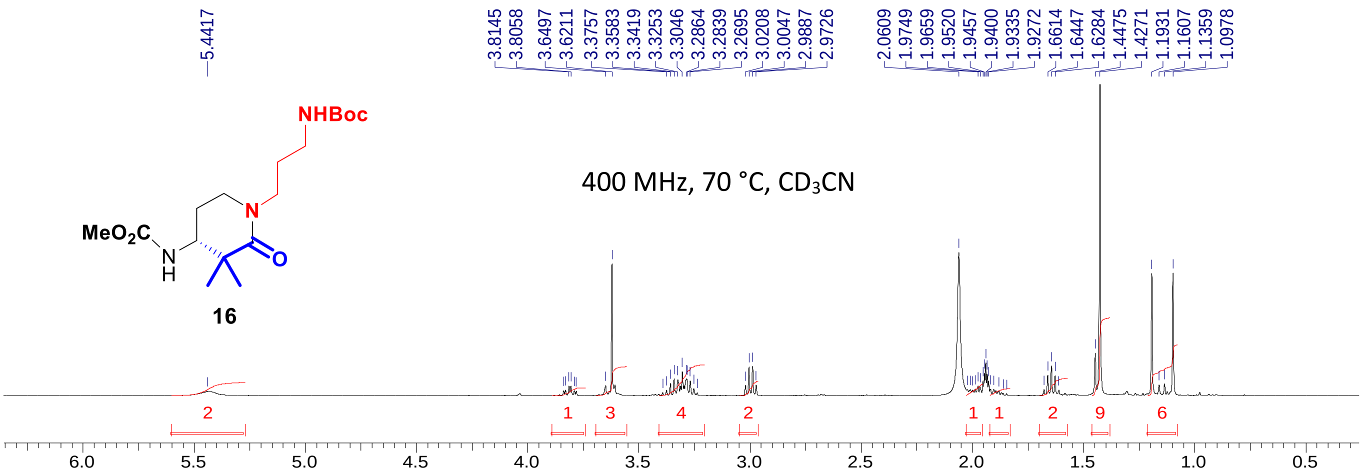
5.4417
3.8145 3.8058 3.6497 3.6211 3.3757 3.3583 3.3419 3.3253 3.3046 3.2864 3.2839 3.2695 3.0208 3.0047 2.9887 2.9726
2.0609 1.9749 1.9659 1.9520 1.9457 1.9400 1.9335 1.9272 1.6614 1.6447 1.6284 1.4475 1.4271 1.1931 1.1607 1.1359 1.0978
NHBoc
N
O
MeO_2C
N
H
16
400 MHz, 70 °C, CD_3CN
2
1
3
4
2
1
1
2
9
6
6.0
5.5
5.0
4.5
4.0
3.5
3.0
2.5
2.0
1.5
1.0
0.5

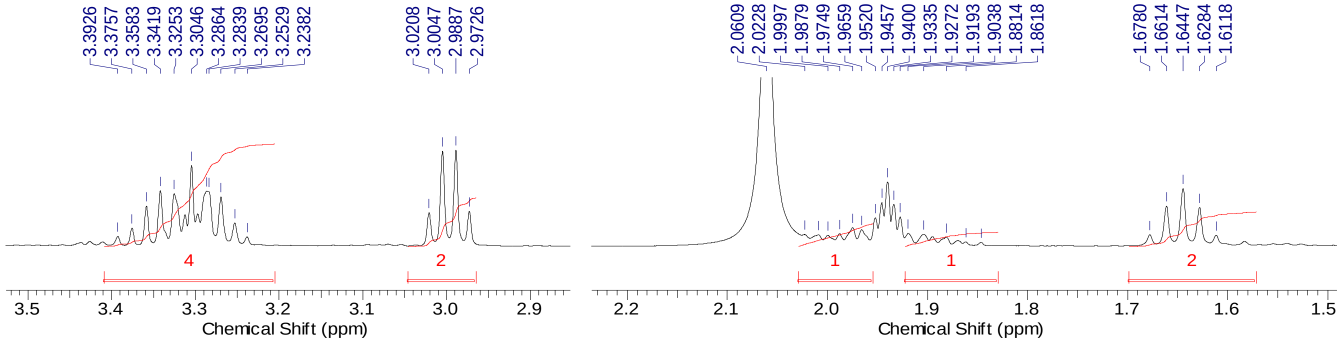
3.3926 3.3757 3.3583 3.3419 3.3253 3.3046 3.2864 3.2839 3.2695 3.2529 3.2382
3.0208 3.0047 2.9887 2.9726
4
2
3.5 3.4 3.3 3.2 3.1 3.0 2.9
Chemical Shift (ppm)
2.0609 2.0228 1.9997 1.9879 1.9749 1.9659 1.9520 1.9457 1.9400 1.9335 1.9272 1.9193 1.9038 1.8814 1.8618
1.6780 1.6614 1.6447 1.6284 1.6118
1
1
2
2.2 2.1 2.0 1.9 1.8 1.7 1.6 1.5
Chemical Shift (ppm)

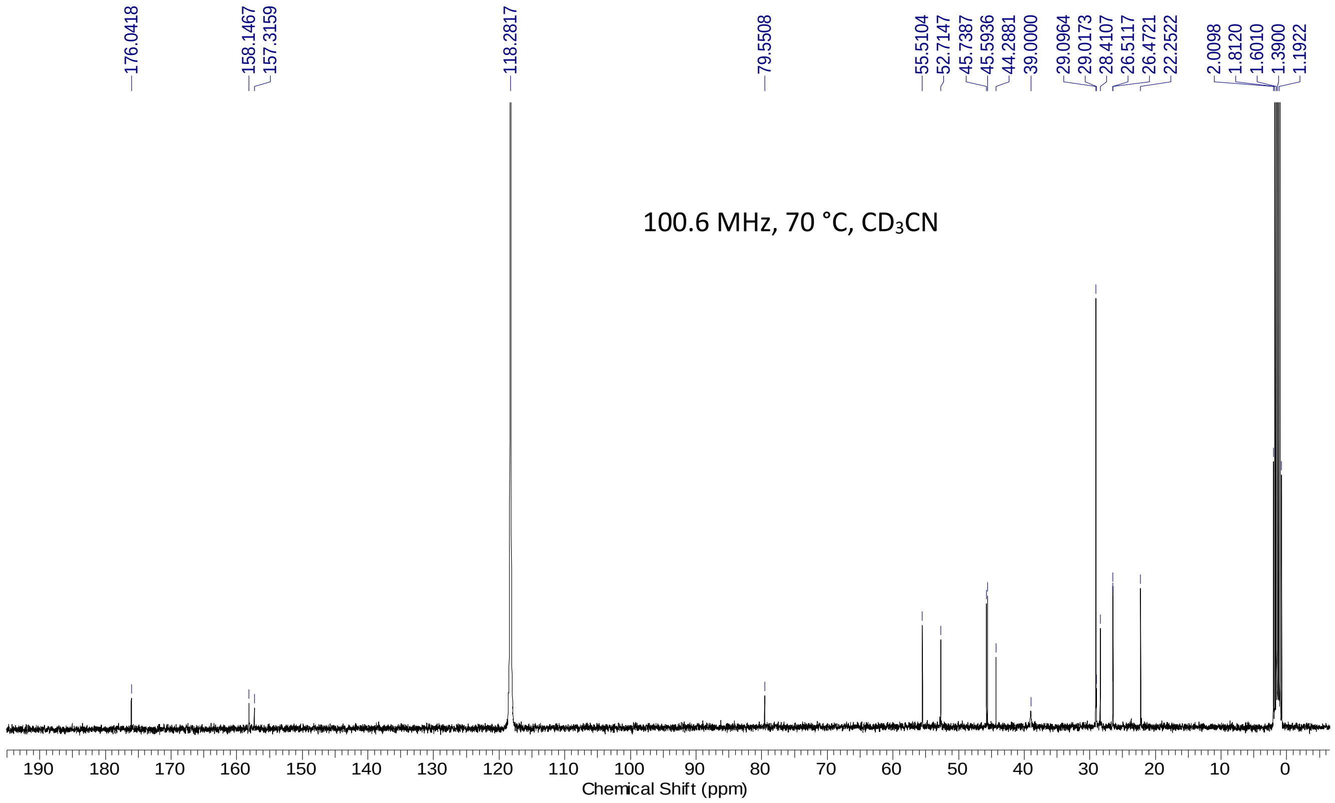
176.0418
158.1467 157.3159
118.2817
79.5508
55.5104 52.7147 45.7387 45.5936 44.2881 39.0000
29.0964 29.0173 28.4107 26.5117 26.4721 22.2522
2.0098 1.8120 1.6010 1.3900 1.1922
100.6 MHz, 70 °C, CD_3CN
190 180 170 160 150 140 130 120 110 100 90 80 70 60 50 40 30 20 10 0
Chemical Shift (ppm)

HSQC, 70 °C, CD_3CN

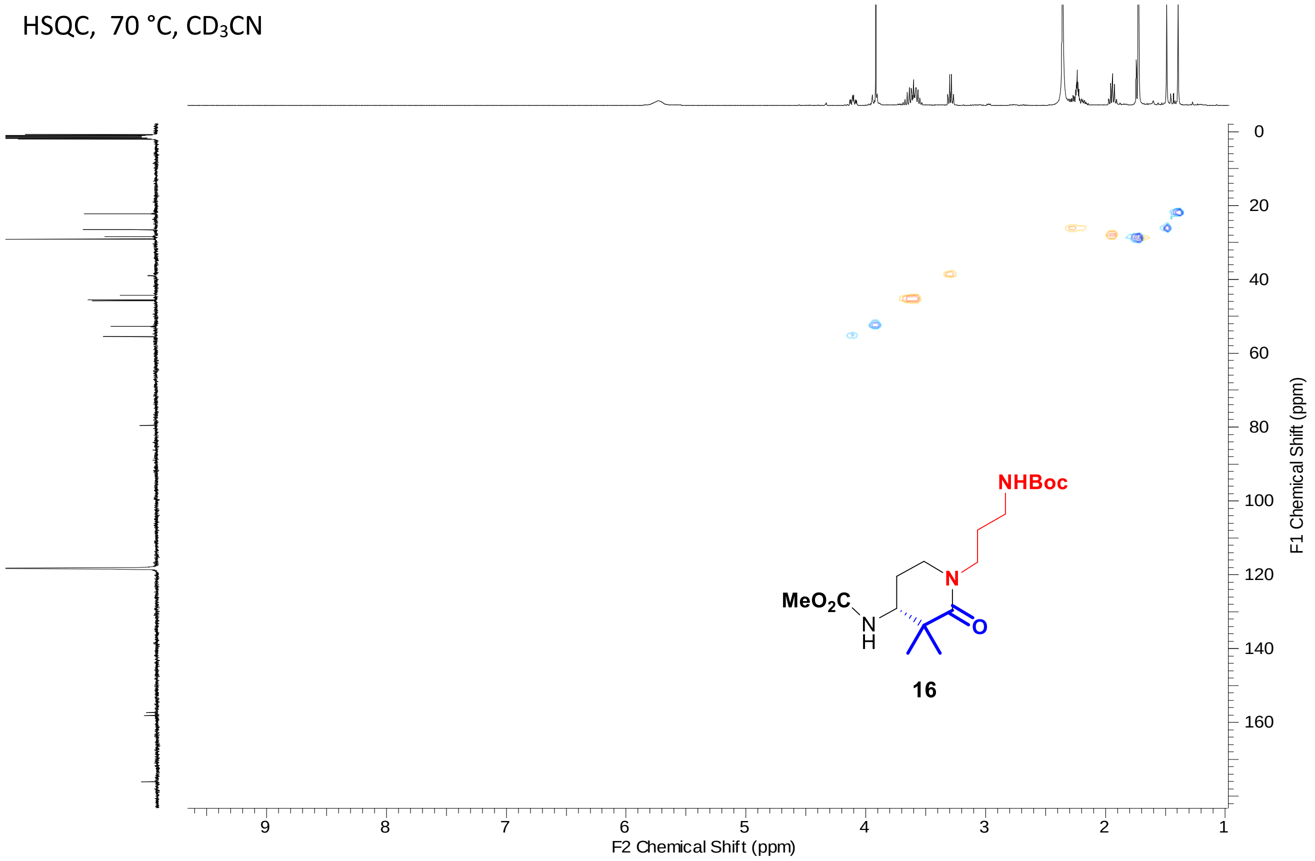

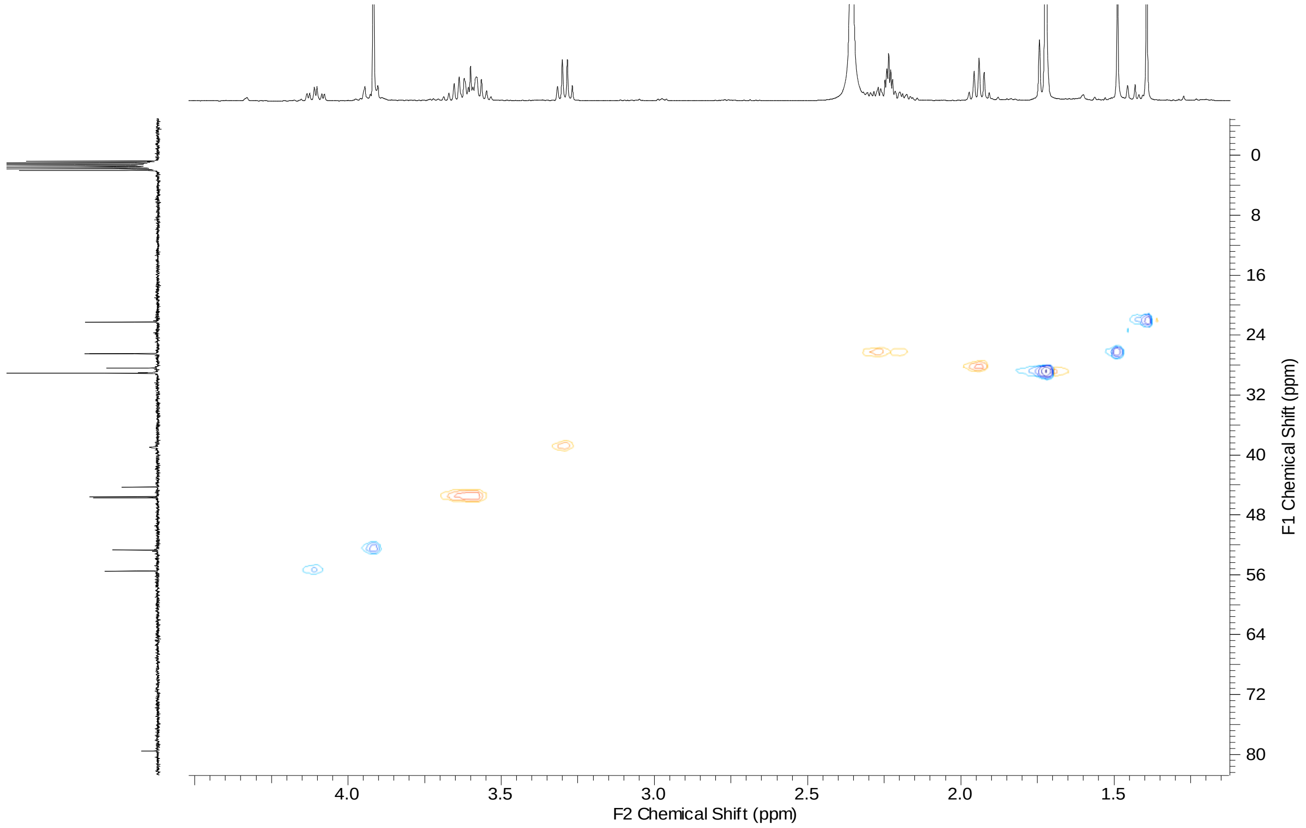

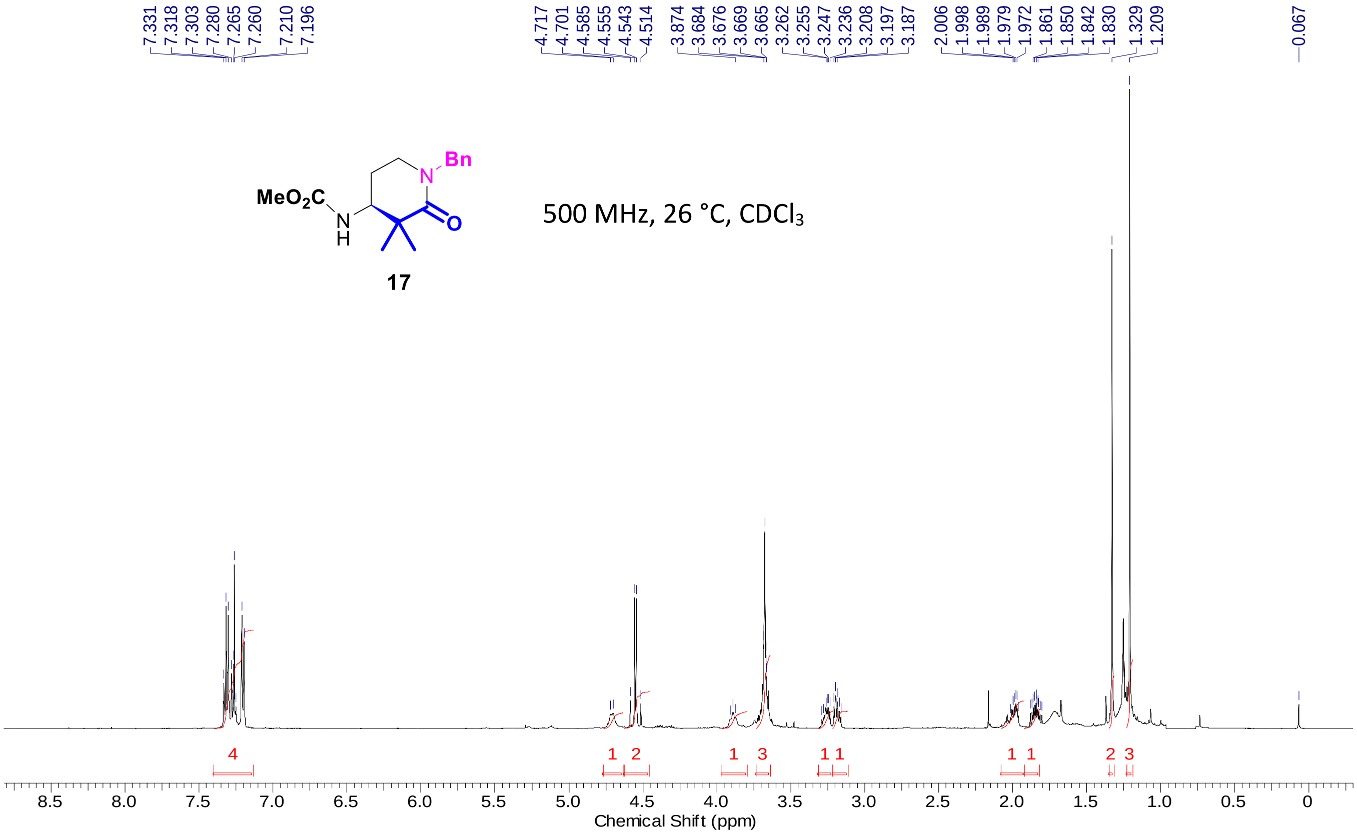
7.331 7.318 7.303 7.280 7.265 7.260 7.210 7.196
4.717 4.701 4.585 4.555 4.543 4.514
3.874 3.684 3.676 3.669 3.665 3.262 3.255 3.247 3.236 3.208 3.197 3.187
2.006 1.998 1.989 1.979 1.972 1.861 1.850 1.842 1.830 1.329 1.209
-0.067
MeO2C
N
H
N
Bn
O
17
500 MHz, 26 °C, CDCl3
4
1 2
1 3
1 1
1 1
2 3
Chemical Shift (ppm)

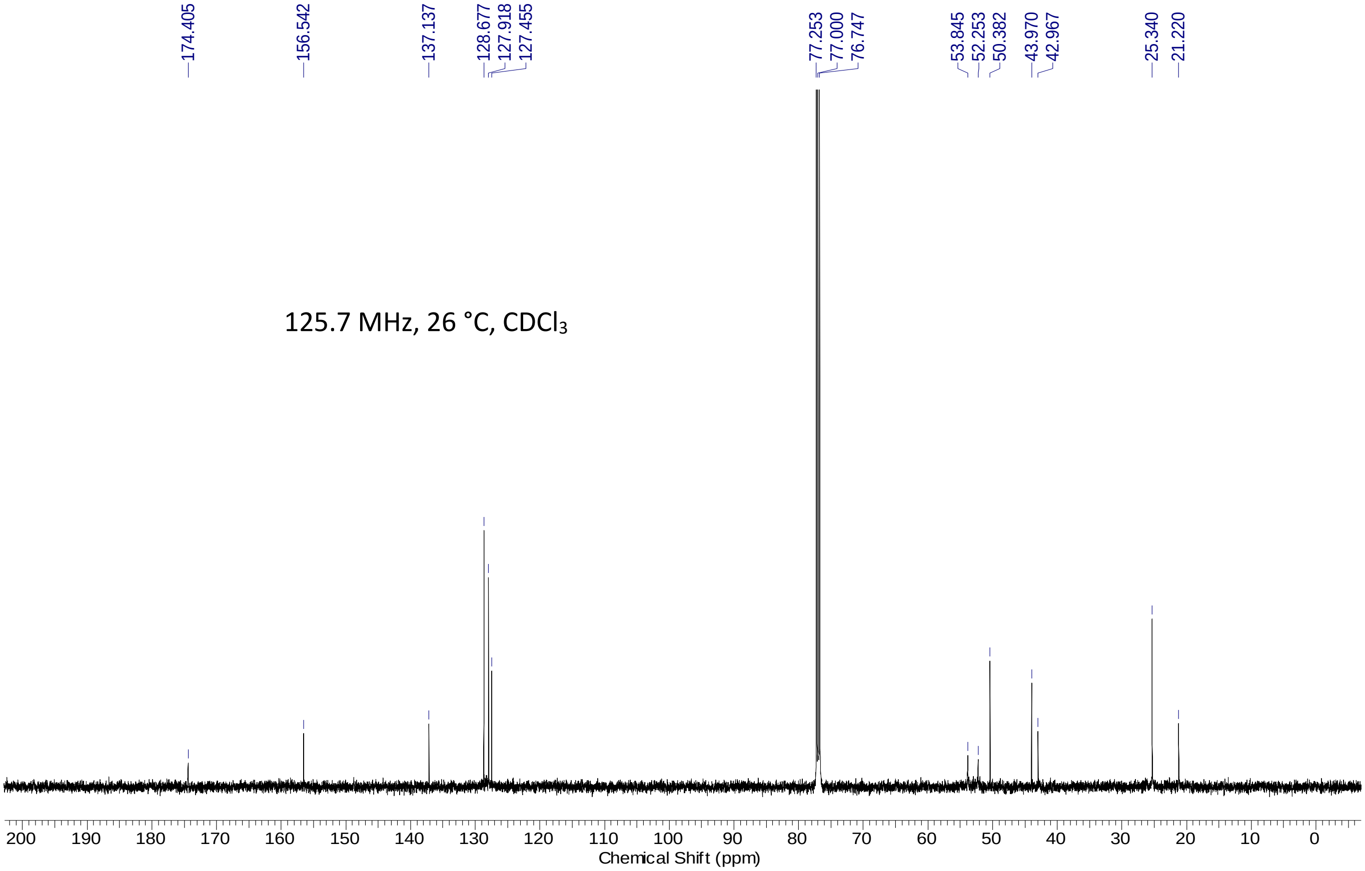
174.405
156.542
137.137
128.677 127.918 127.455
77.253 77.000 76.747
53.845 52.253 50.382
43.970 42.967
25.340 21.220
125.7 MHz, 26 °C, CDCl3
Chemical Shift (ppm)

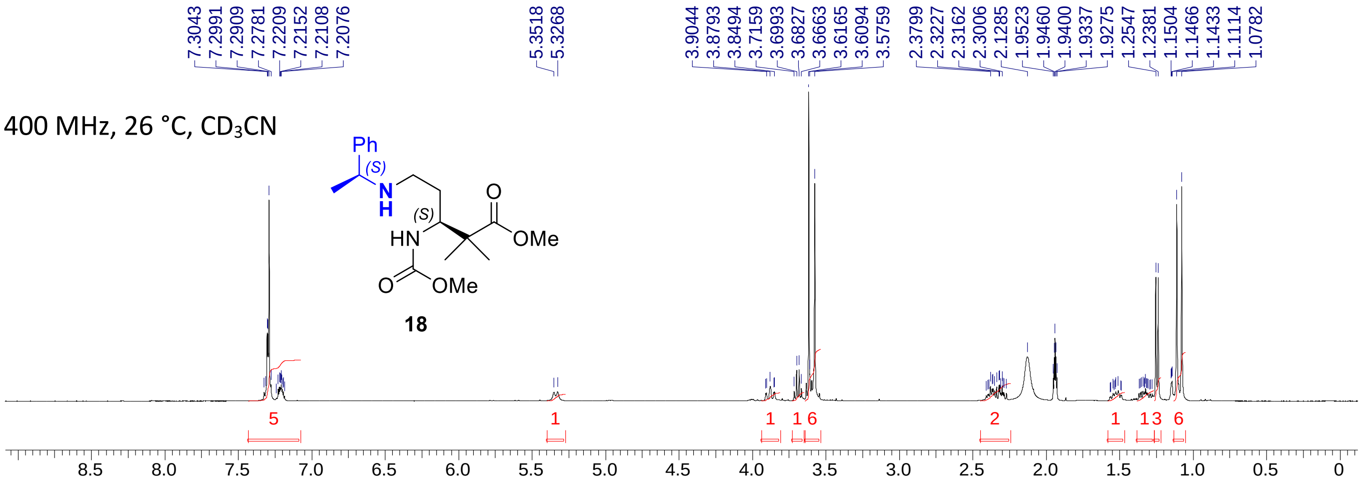
7.3043 7.2991 7.2909 7.2781 7.2209 7.2152 7.2108 7.2076
5.3518 5.3268
3.9044 3.8793 3.8494 3.7159 3.6993 3.6827 3.6663 3.6165 3.6094 3.5759
2.3799 2.3227 2.3162 2.3006 2.1285 1.9523 1.9460 1.9400 1.9337 1.9275
1.2547 1.2381 1.1504 1.1466 1.1433 1.1114 1.0782
400 MHz, 26 °C, CD3CN
Ph
(S)
N
H
(S)
HN
O
OMe
O
OMe
18
5
1
1
1
6
2
1
1
3
6
8.5 8.0 7.5 7.0 6.5 6.0 5.5 5.0 4.5 4.0 3.5 3.0 2.5 2.0 1.5 1.0 0.5 0

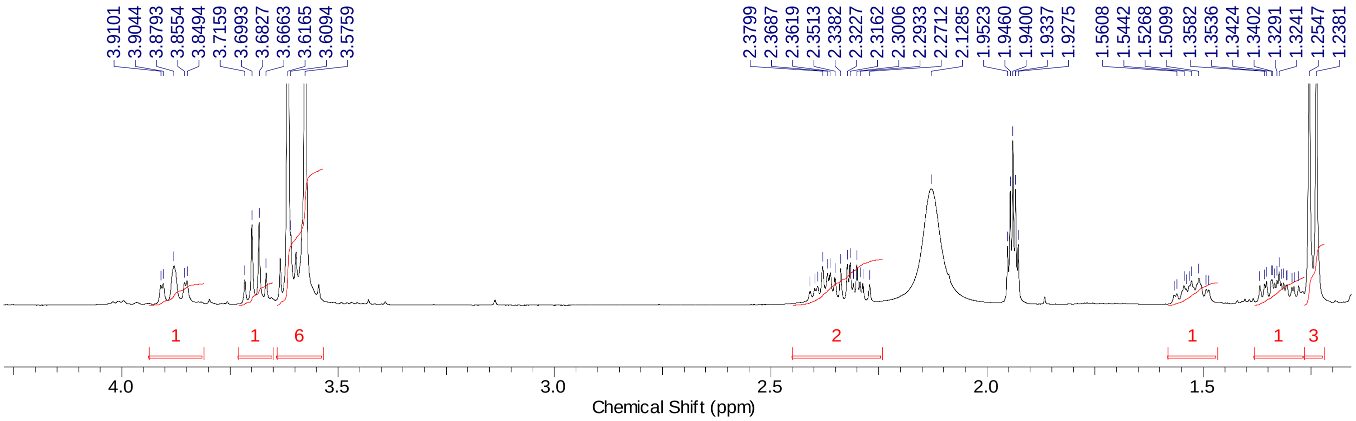
3.9101 3.9044 3.8793 3.8554 3.8494 3.7159 3.6993 3.6827 3.6663 3.6165 3.6094 3.5759
2.3799 2.3687 2.3619 2.3513 2.3382 2.3227 2.3162 2.3006 2.2933 2.2712 2.1285 1.9523 1.9460 1.9400 1.9337 1.9275
1.5608 1.5442 1.5268 1.5099 1.3582 1.3536 1.3424 1.3402 1.3291 1.3241 1.2547 1.2381
1
1
6
2
1
1
3
4.0 3.5 3.0 2.5 2.0 1.5
Chemical Shift (ppm)

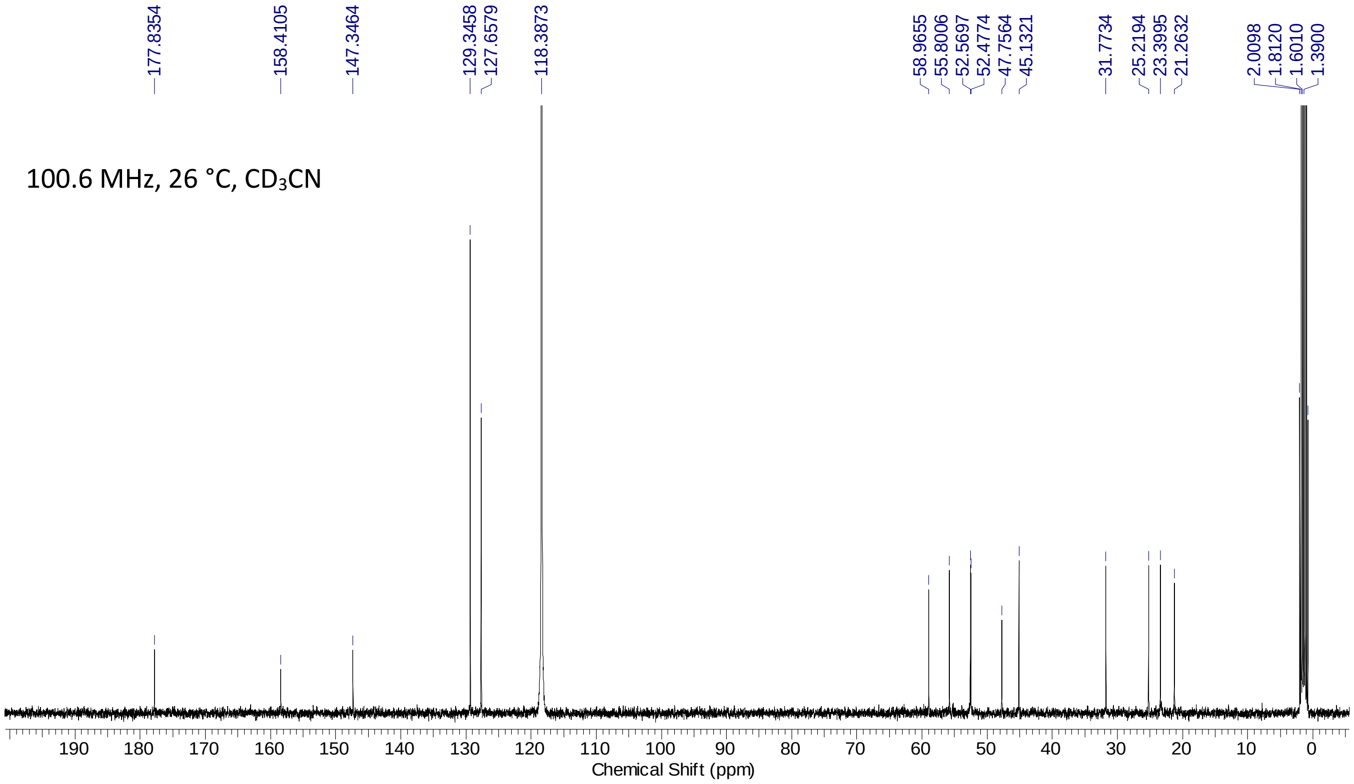
177.8354
158.4105
147.3464
129.3458 127.6579
118.3873
58.9655 55.8006 52.5697 52.4774 47.7564 45.1321
31.7734
25.2194 23.3995 21.2632
2.0098 1.8120 1.6010 1.3900
100.6 MHz, 26 °C, CD3CN
190 180 170 160 150 140 130 120 110 100 90 80 70 60 50 40 30 20 10 0
Chemical Shift (ppm)

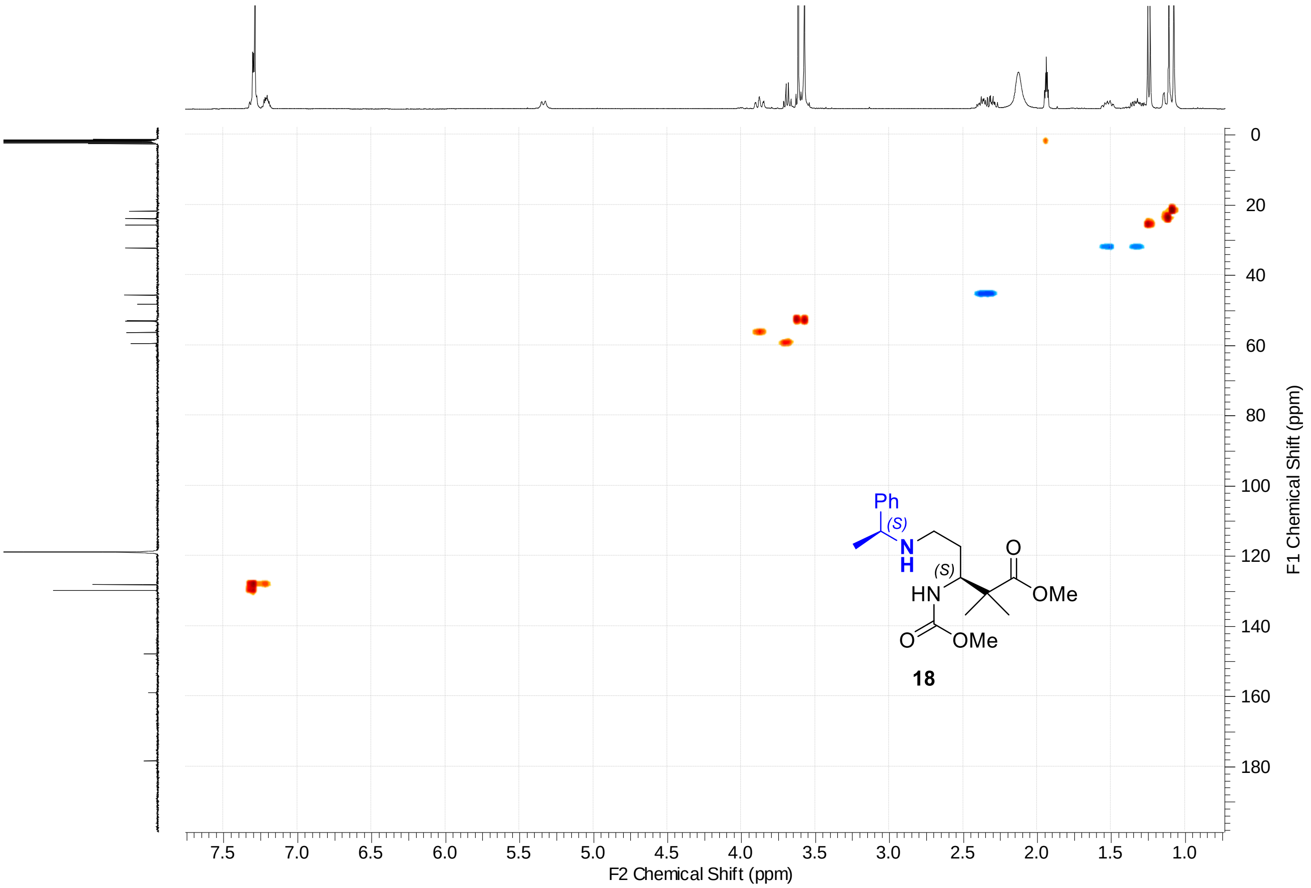

HSQC, 26 °C, CD_3CN

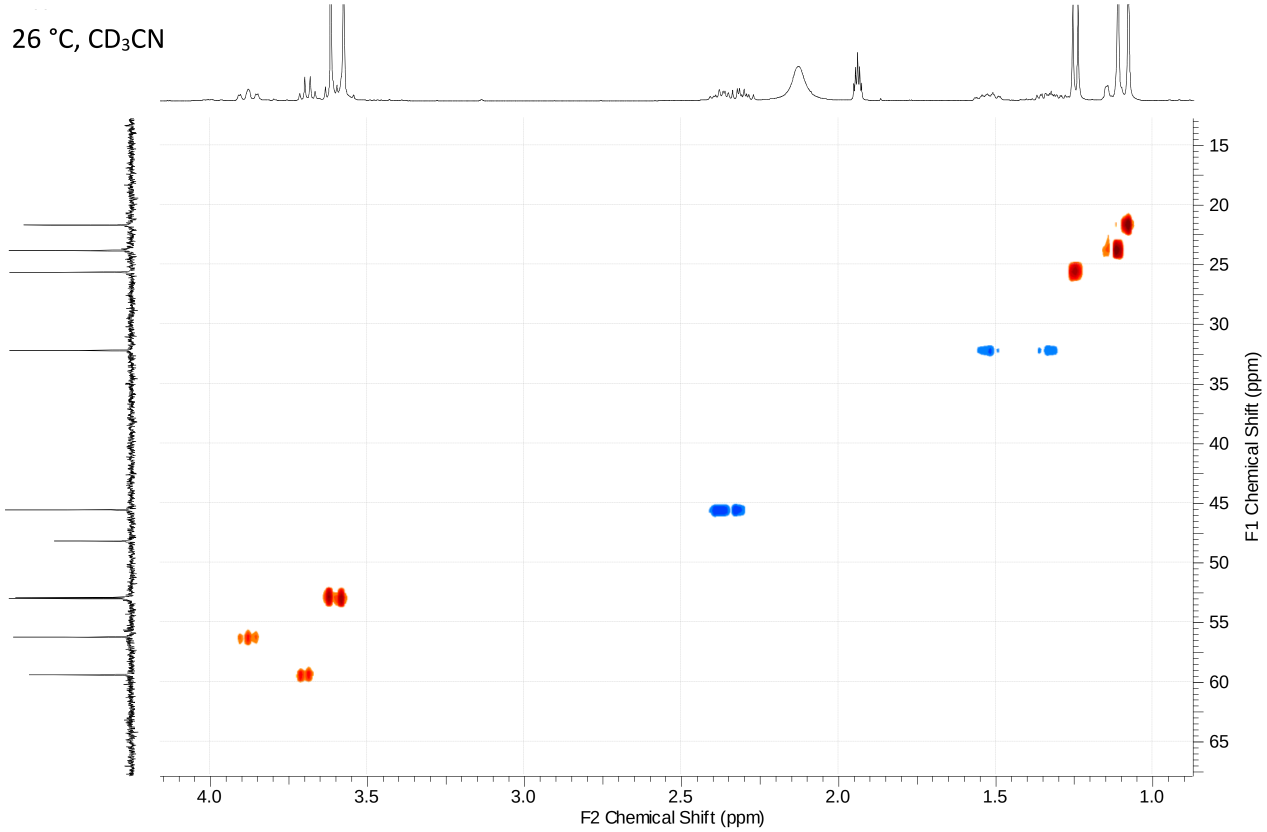

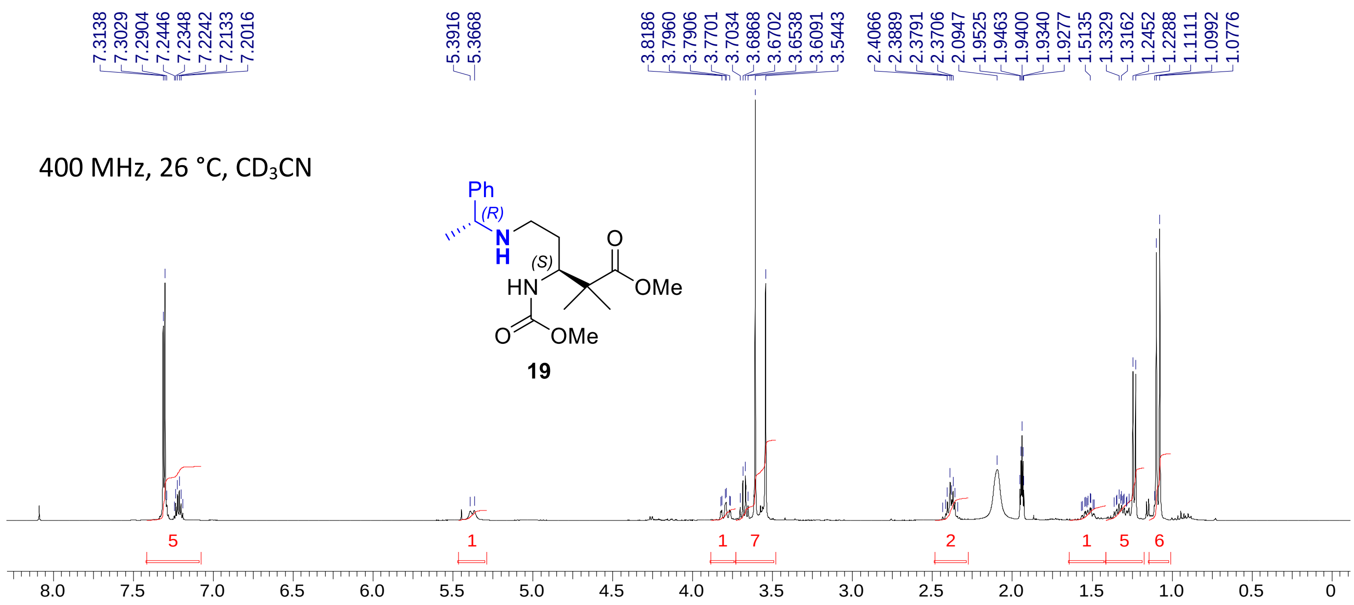
400 MHz, 26 °C, CD3CN
Ph
(R)
N
H
(S)
HN
O
OMe
O
OMe
19
5
1
1
7
2
1
5
6

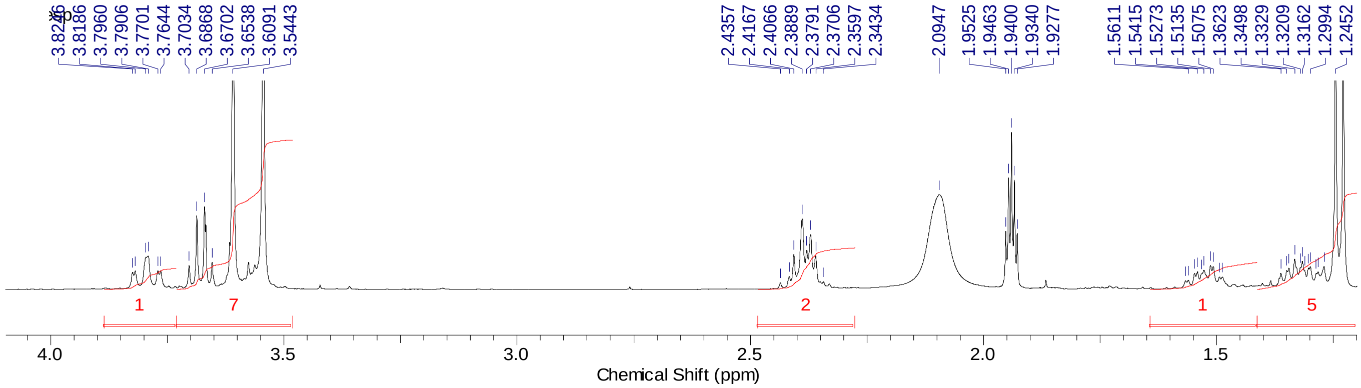
1
7
2
1
5
Chemical Shift (ppm)

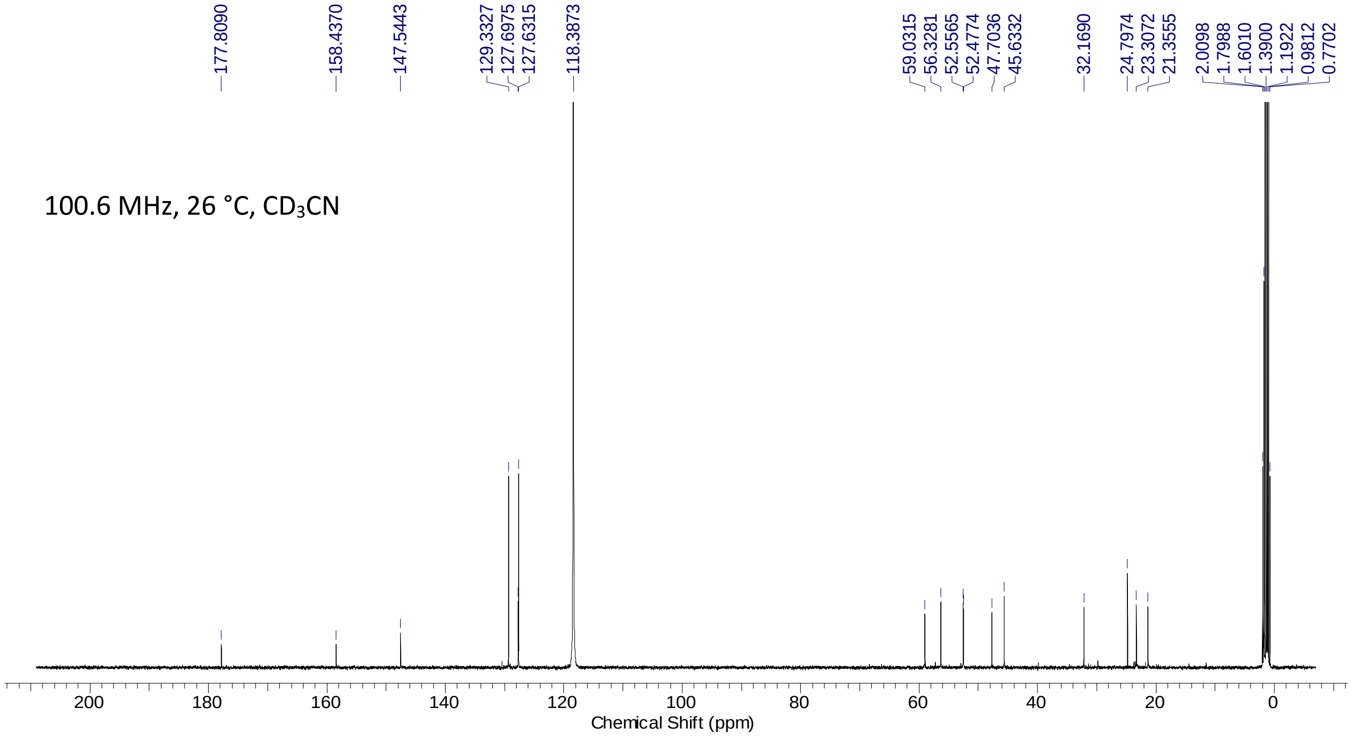
100.6 MHz, 26 °C, CD3CN
Chemical Shift (ppm)

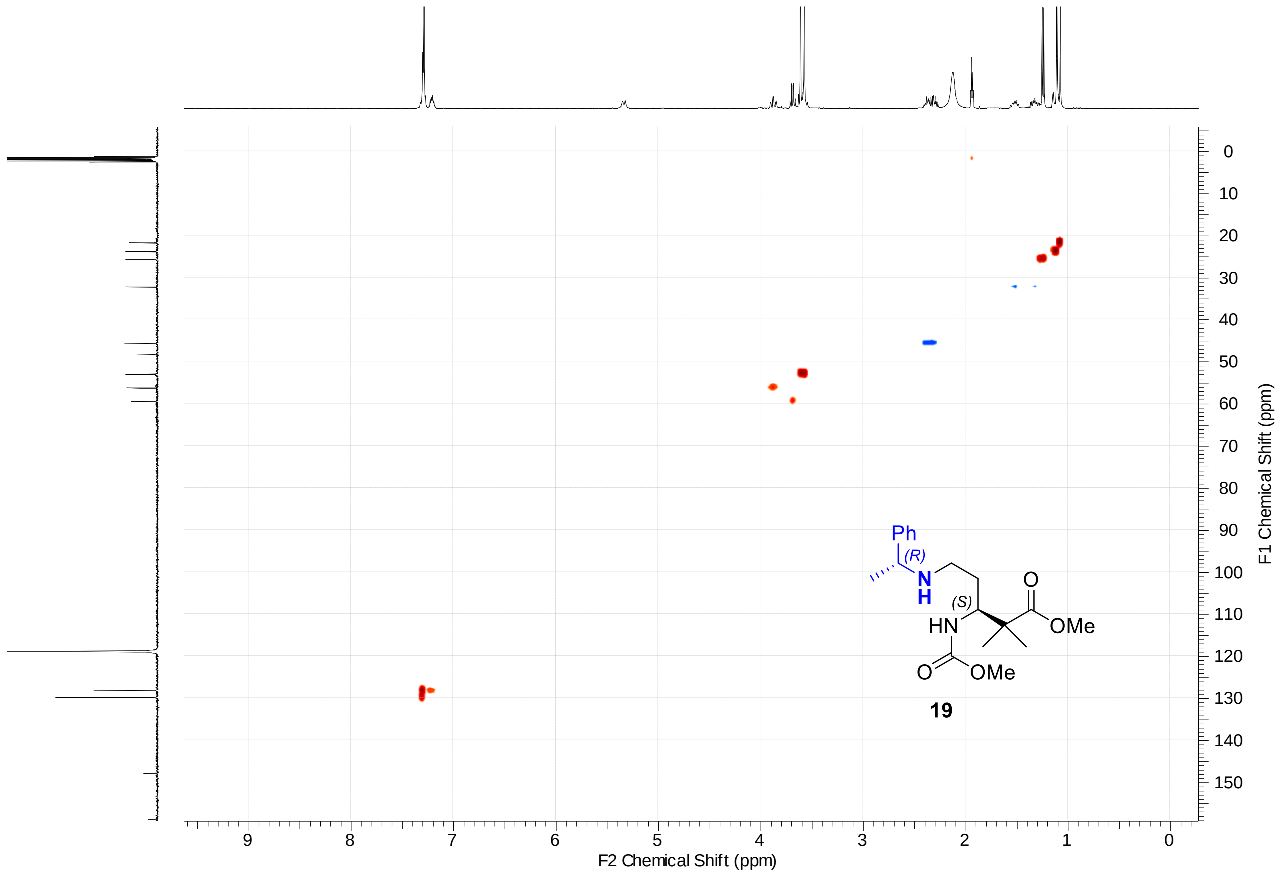

HSQC, 70 °C, CD_3CN

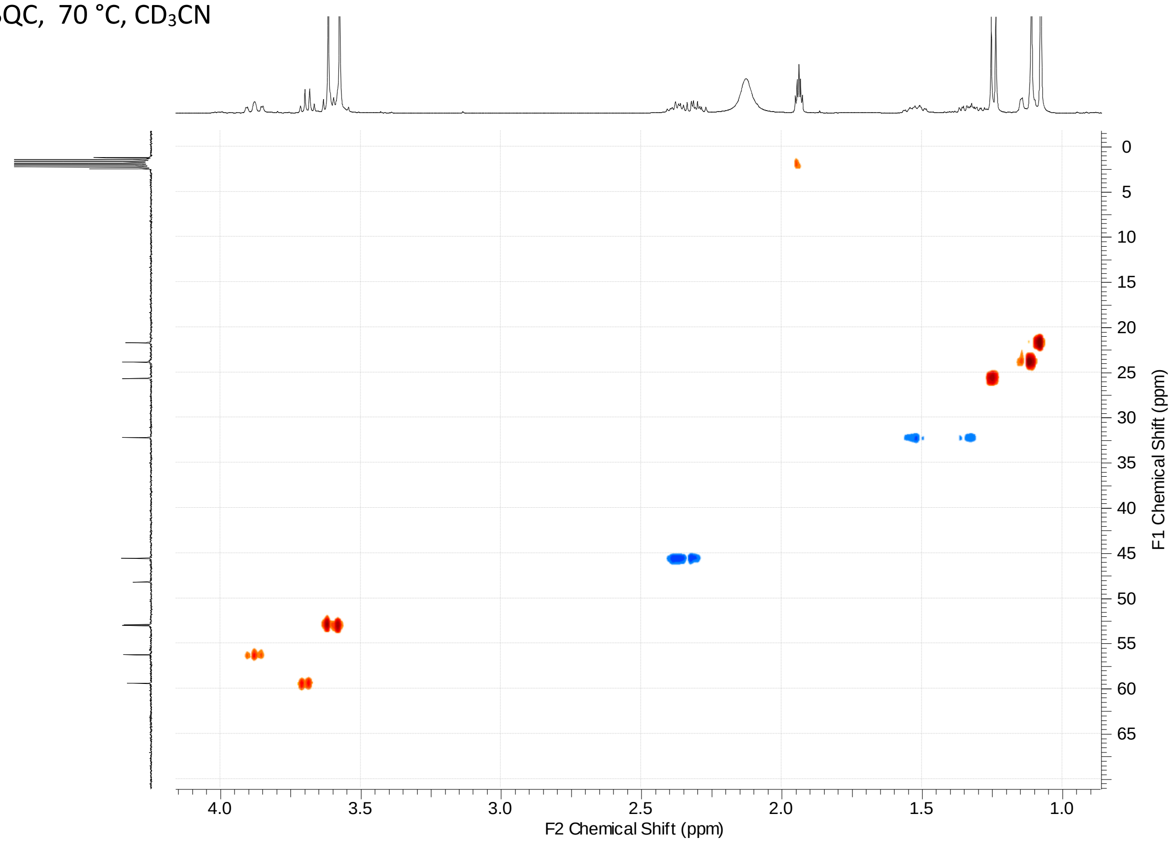

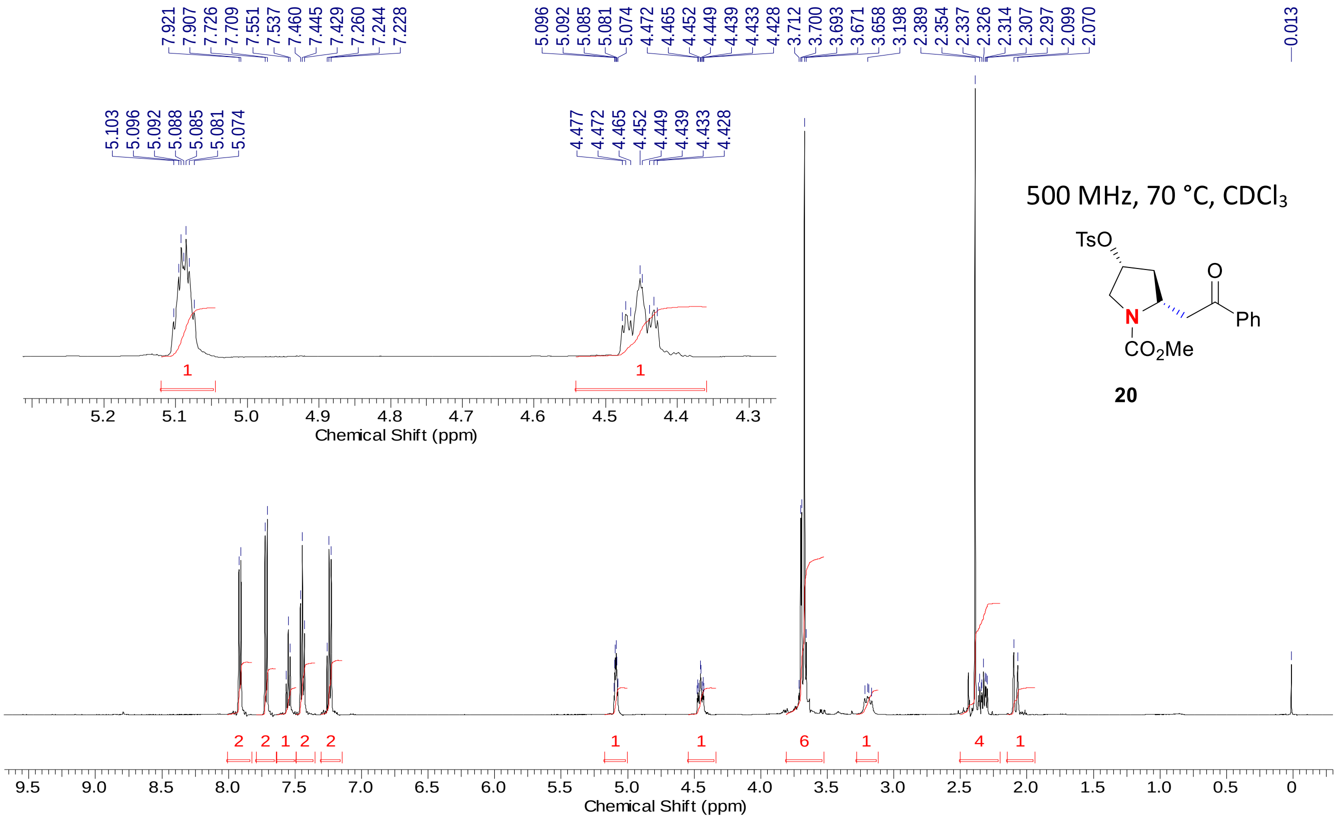
7.921 7.907 7.726 7.709 7.551 7.537 7.460 7.445 7.429 7.260 7.244 7.228
5.096 5.092 5.085 5.081 5.074 4.472 4.465 4.452 4.449 4.439 4.433 4.428 3.712 3.700 3.693 3.671 3.658 3.198 2.389 2.354 2.337 2.326 2.314 2.307 2.297 2.099 2.070
0.013
5.103 5.096 5.092 5.088 5.085 5.081 5.074
4.477 4.472 4.465 4.452 4.449 4.439 4.433 4.428
500 MHz, 70 °C, $CDCl_3$
Chemical Shift (ppm)
Chemical Shift (ppm)
TsO
O
N
Ph
CO_2Me

20

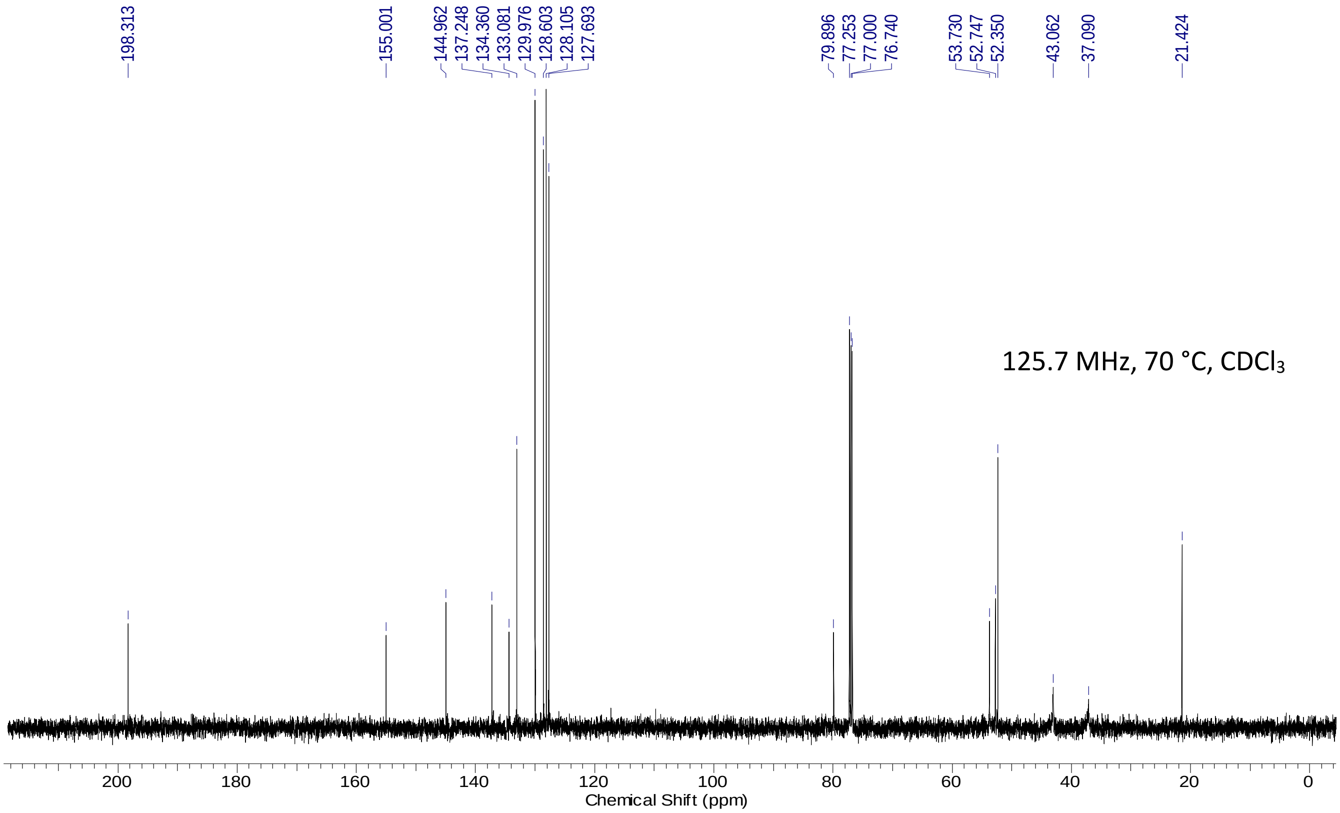
198.313
155.001
144.962 137.248 134.360 133.081 129.976 128.603 128.105 127.693
79.896 77.253 77.000 76.740
53.730 52.747 52.350
43.062
37.090
21.424
125.7 MHz, 70 °C, $CDCl_3$
Chemical Shift (ppm)

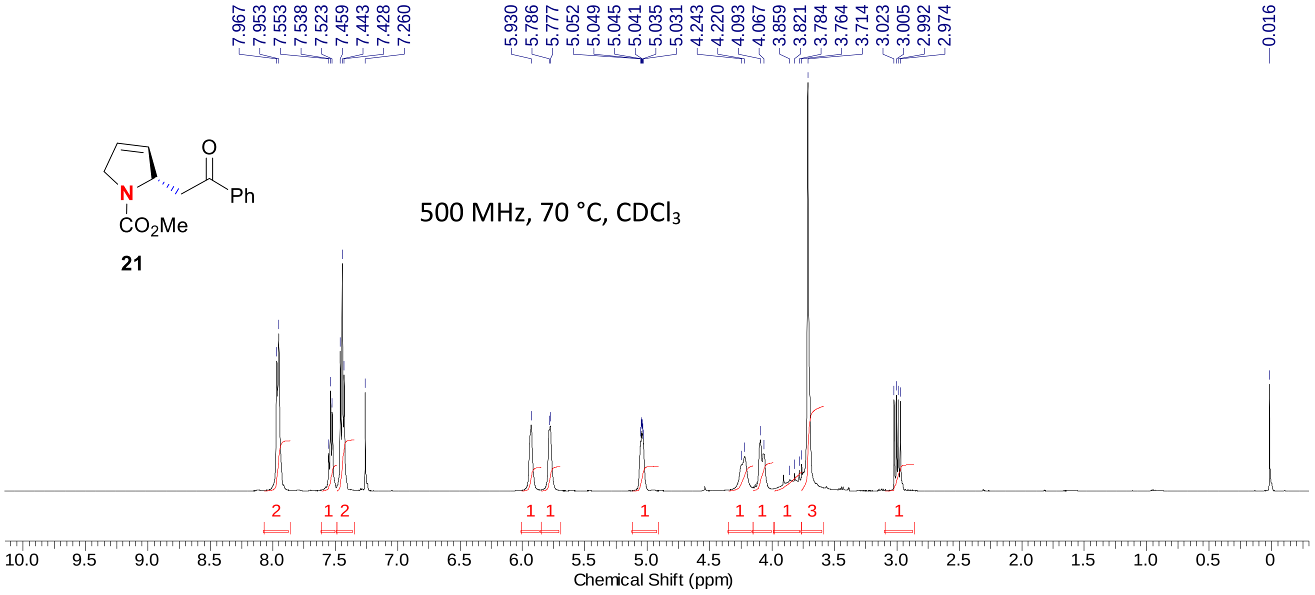
7.967 7.953 7.553 7.538 7.523 7.459 7.443 7.428 7.260
5.930 5.786 5.777 5.052 5.049 5.045 5.041 5.035 5.031 4.243 4.220 4.093 4.067 3.859 3.821 3.784 3.764 3.714 3.023 3.005 2.992 2.974
0.016
O
Ph
N
CO_2Me
21
500 MHz, 70 °C, $CDCl_3$
2 1 2 1 1 1 1 1 1 3 1
Chemical Shift (ppm)

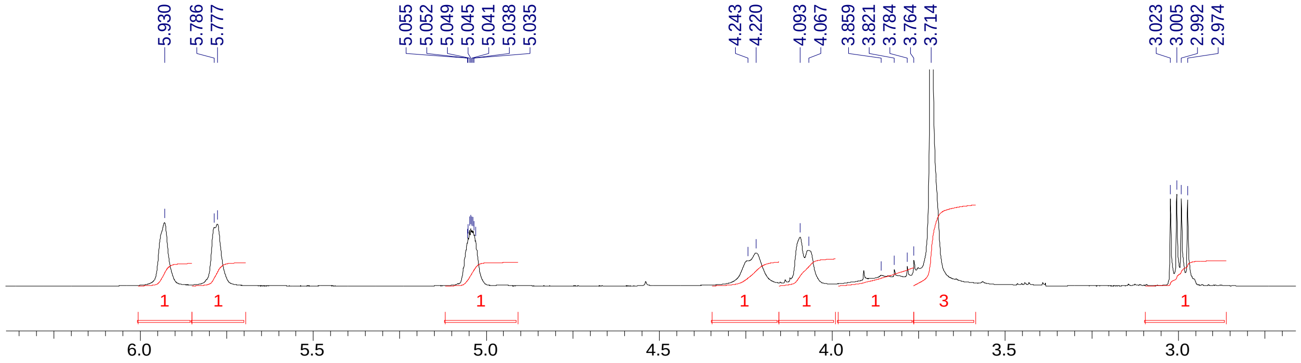
5.930 5.786 5.777
5.055 5.052 5.049 5.045 5.041 5.038 5.035
4.243 4.220 4.093 4.067 3.859 3.821 3.784 3.764 3.714
3.023 3.005 2.992 2.974
1 1 1 1 1 1 3 1

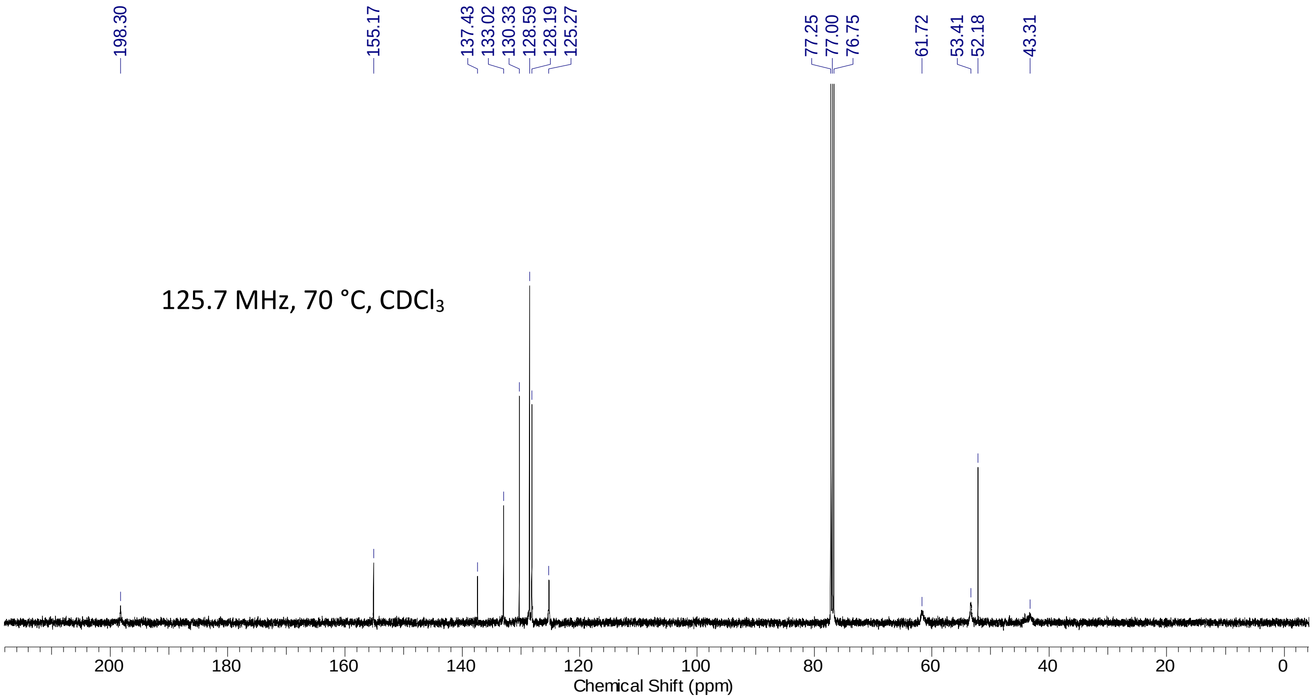
198.30
155.17
137.43 133.02 130.33 128.59 128.19 125.27
77.25 77.00 76.75
61.72
53.41 52.18
43.31
125.7 MHz, 70 °C, $CDCl_3$
Chemical Shift (ppm)

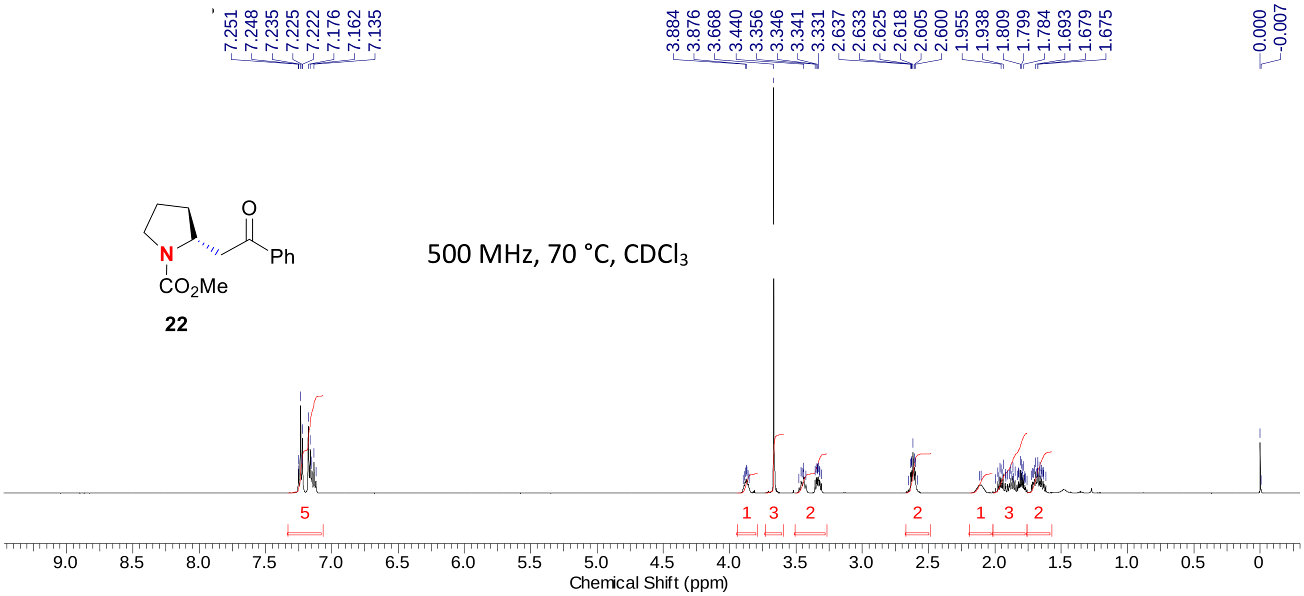
7.251 7.248 7.235 7.225 7.222 7.176 7.162 7.135
3.884 3.876 3.668 3.440 3.356 3.346 3.341 3.331 2.637 2.633 2.625 2.618 2.605 2.600 1.955 1.938 1.809 1.799 1.784 1.693 1.679 1.675
0.000 -0.007
O
Ph
N
CO_2Me
22
500 MHz, 70 °C, $CDCl_3$
5 1 3 2 2 1 3 2
9.0 8.5 8.0 7.5 7.0 6.5 6.0 5.5 5.0 4.5 4.0 3.5 3.0 2.5 2.0 1.5 1.0 0.5 0
Chemical Shift (ppm)

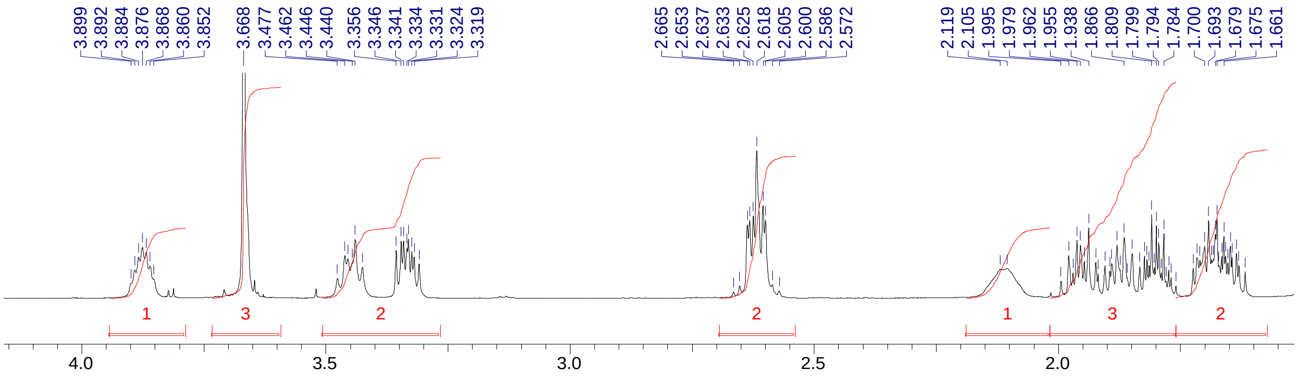
3.899 3.892 3.884 3.876 3.868 3.860 3.852 3.668 3.477 3.462 3.446 3.440 3.356 3.346 3.341 3.334 3.331 3.324 3.319
2.665 2.653 2.637 2.633 2.625 2.618 2.605 2.600 2.586 2.572
2.119 2.105 1.995 1.979 1.962 1.955 1.938 1.866 1.809 1.799 1.794 1.784 1.700 1.693 1.679 1.675 1.661
1 3 2 2 1 3 2
4.0 3.5 3.0 2.5 2.0

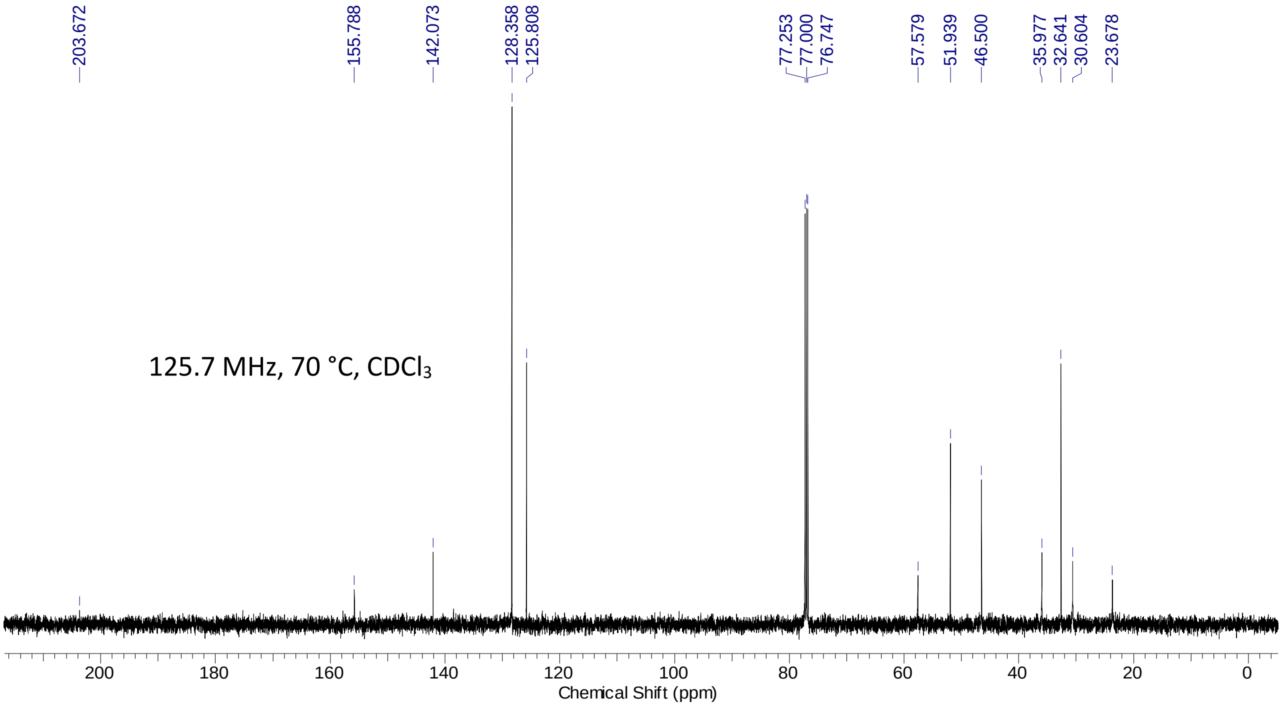
203.672 155.788 142.073 128.358 125.808 77.253 77.000 76.747 57.579 51.939 46.500 35.977 32.641 30.604 23.678
125.7 MHz, 70 °C, $CDCl_3$
200 180 160 140 120 100 80 60 40 20 0
Chemical Shift (ppm)

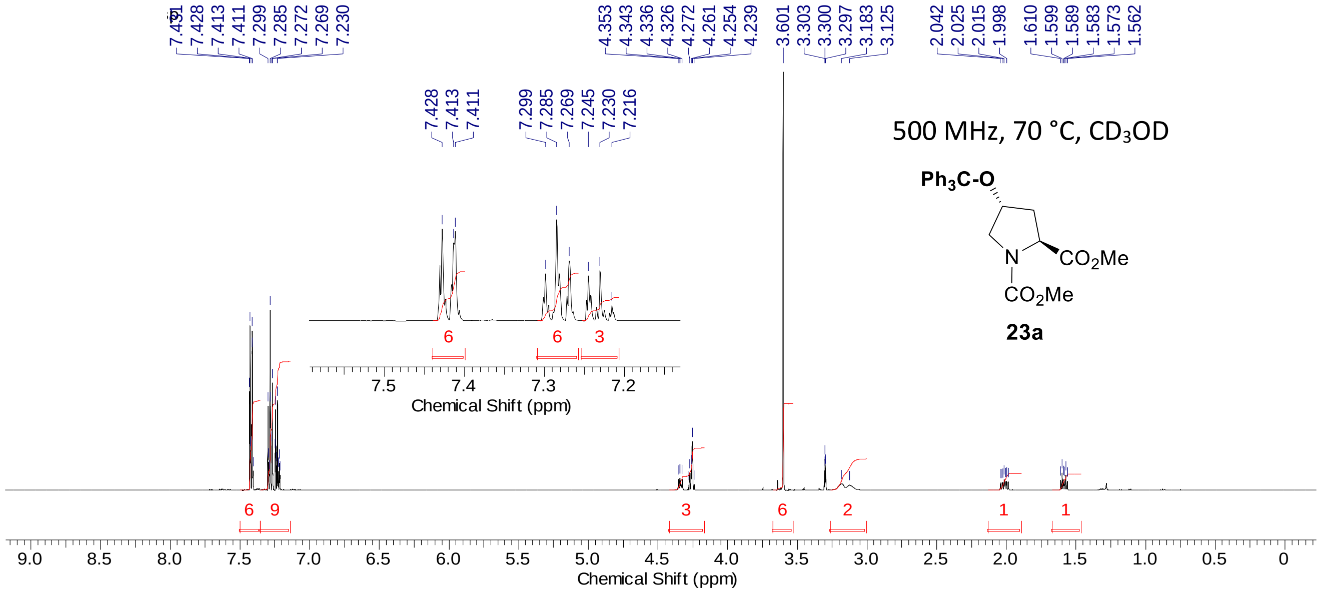
500 MHz, 70 °C, CD3OD
Ph3C-O
CO2Me
N
CO2Me
23a
Chemical Shift (ppm)

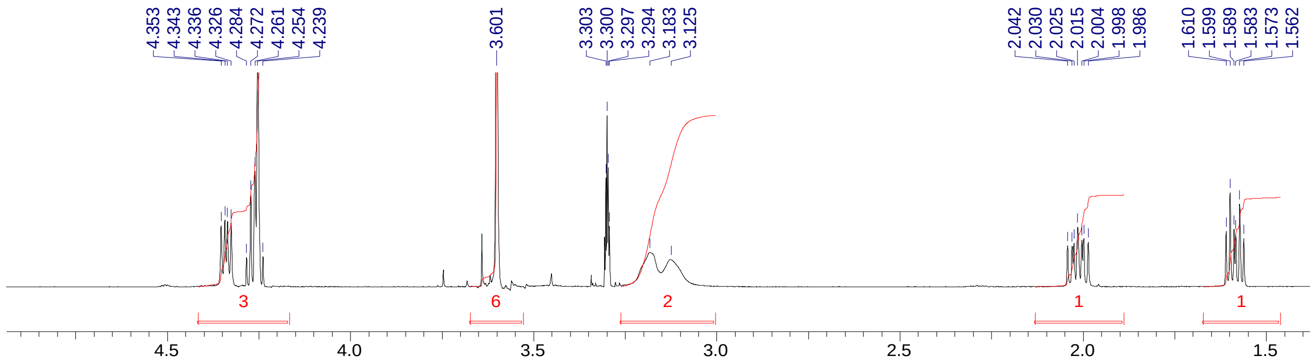
Chemical Shift (ppm)

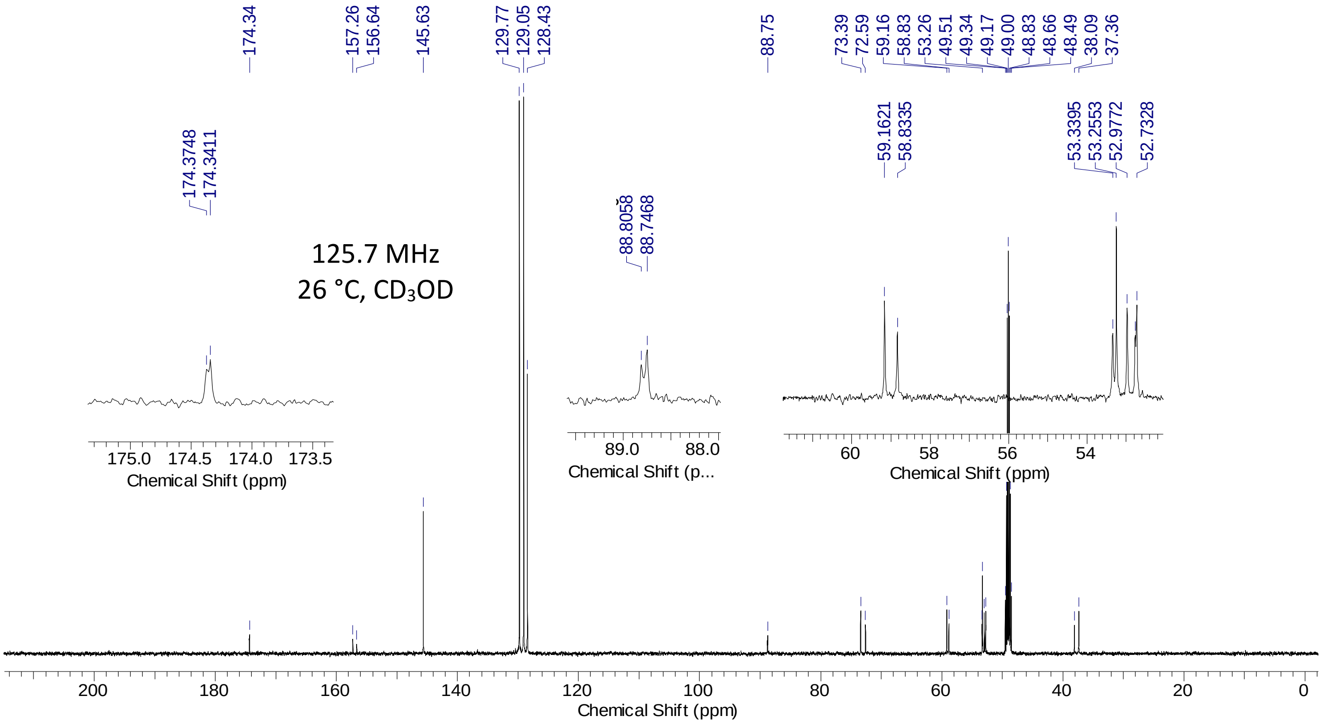
125.7 MHz
26 °C, CD3OD
Chemical Shift (ppm)

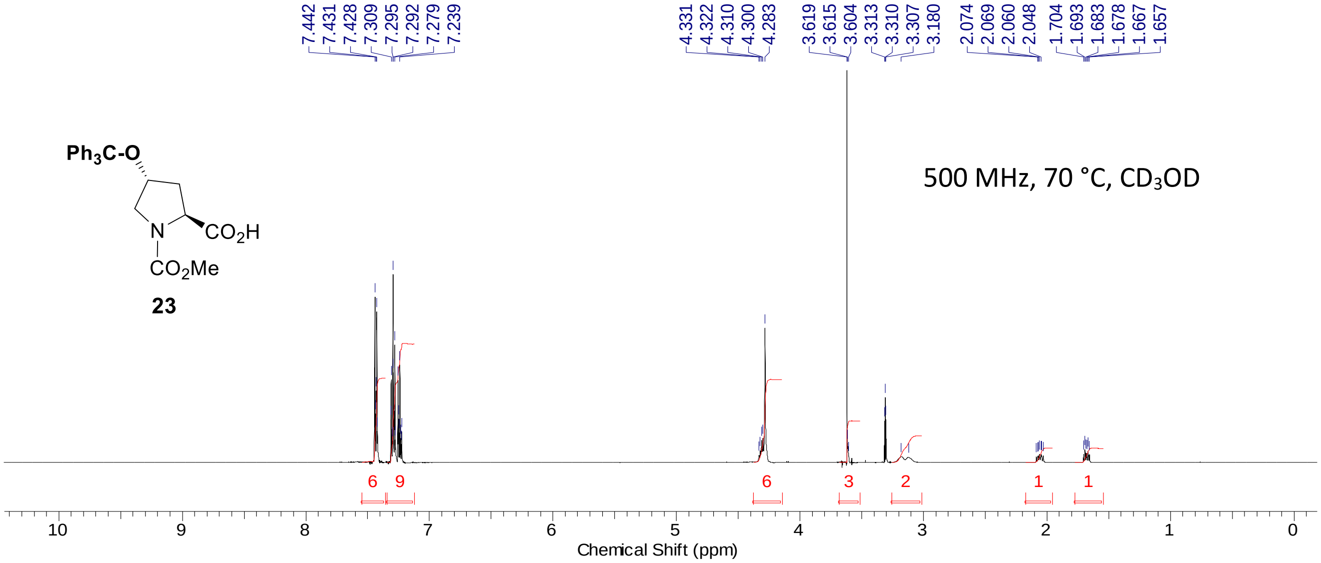
Ph3C-O
N
CO2H
CO2Me
23
7.442
7.431
7.428
7.309
7.295
7.292
7.279
7.239
4.331
4.322
4.310
4.300
4.283
3.619
3.615
3.604
3.313
3.310
3.307
3.180
2.074
2.069
2.060
2.048
1.704
1.693
1.683
1.678
1.667
1.657
500 MHz, 70 °C, CD3OD
6
9
6
3
2
1
1
10
9
8
7
6
5
4
3
2
1
0
Chemical Shift (ppm)

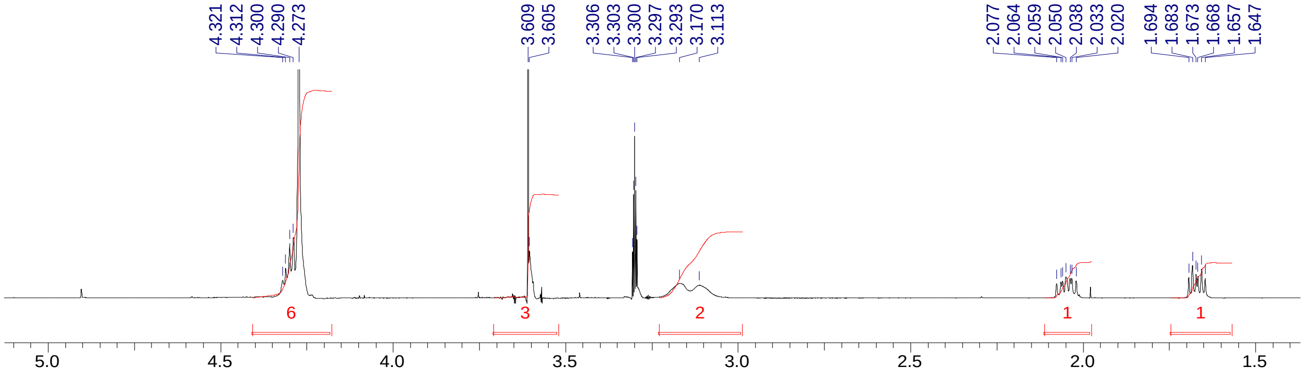
4.321
4.312
4.300
4.290
4.273
3.609
3.605
3.306
3.303
3.300
3.297
3.293
3.170
3.113
2.077
2.064
2.059
2.050
2.038
2.033
2.020
1.694
1.683
1.673
1.668
1.657
1.647
6
3
2
1
1
5.0
4.5
4.0
3.5
3.0
2.5
2.0
1.5

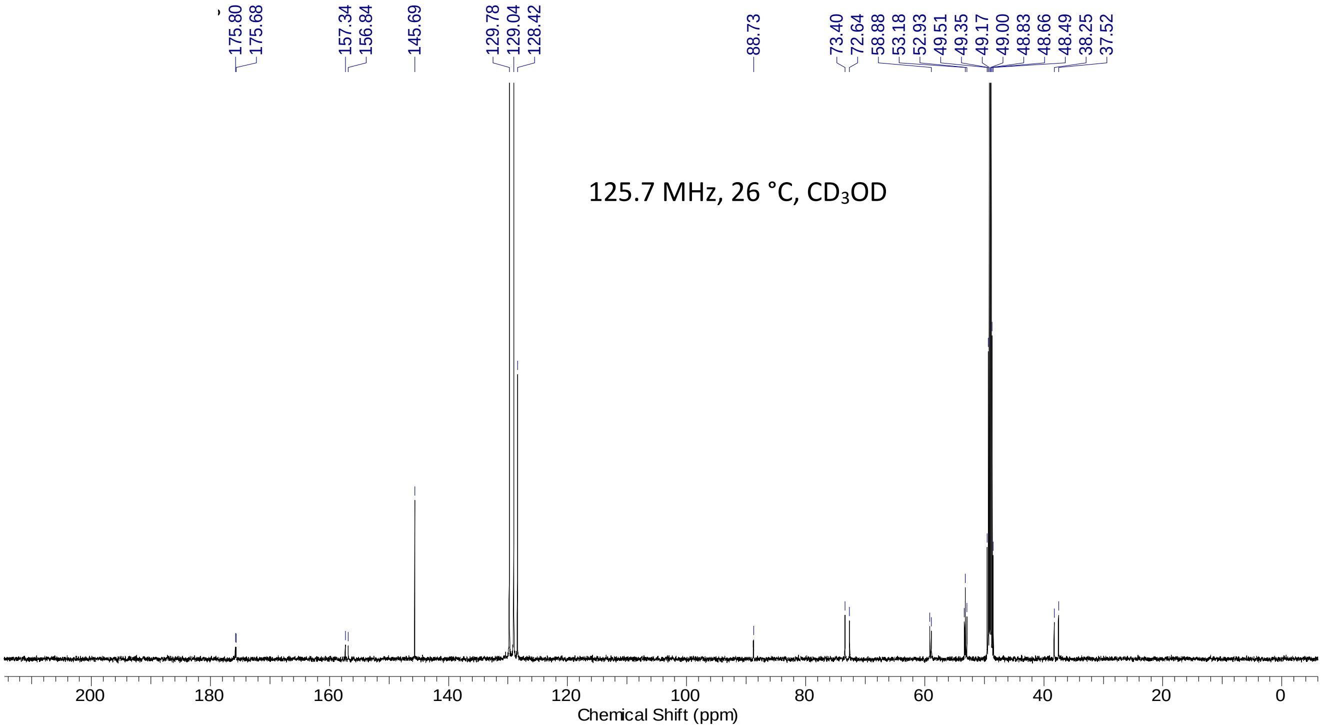
175.80
175.68
157.34
156.84
145.69
129.78
129.04
128.42
88.73
73.40
72.64
58.88
53.18
52.93
49.51
49.35
49.17
49.00
48.83
48.66
48.49
38.25
37.52
125.7 MHz, 26 °C, CD3OD
200
180
160
140
120
100
80
60
40
20
0
Chemical Shift (ppm)

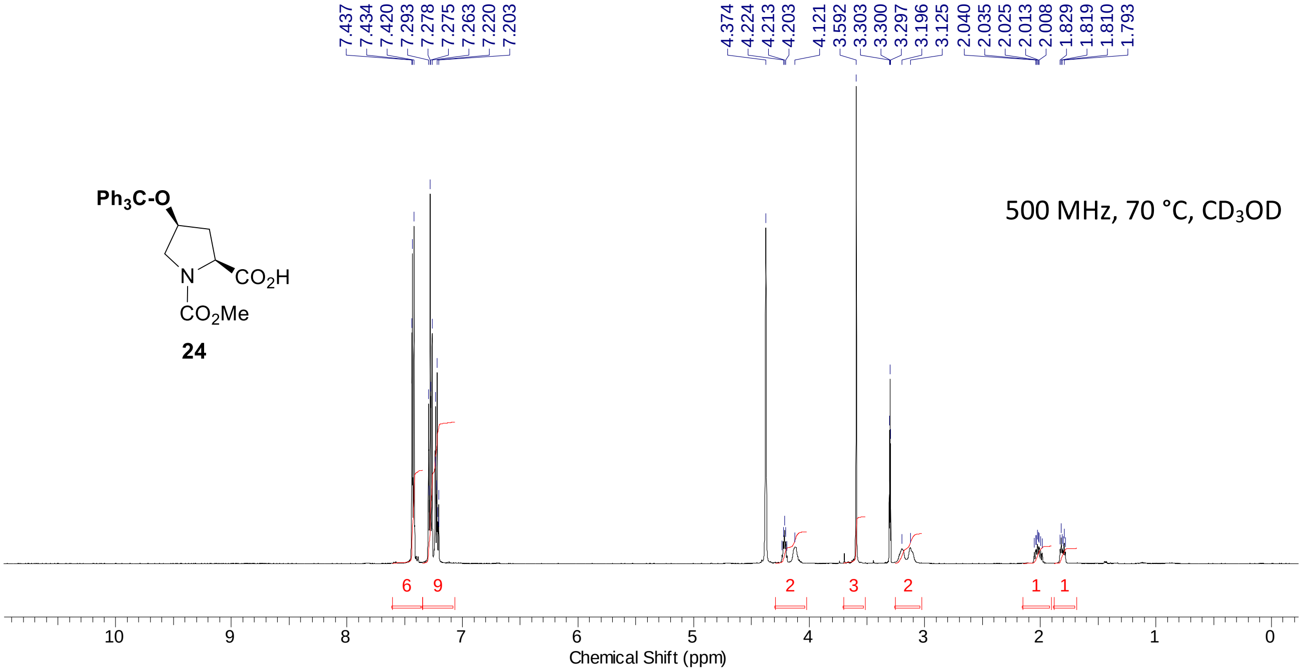
Ph3C-O
CO2H
N
CO2Me
24
500 MHz, 70 °C, CD3OD
Chemical Shift (ppm)

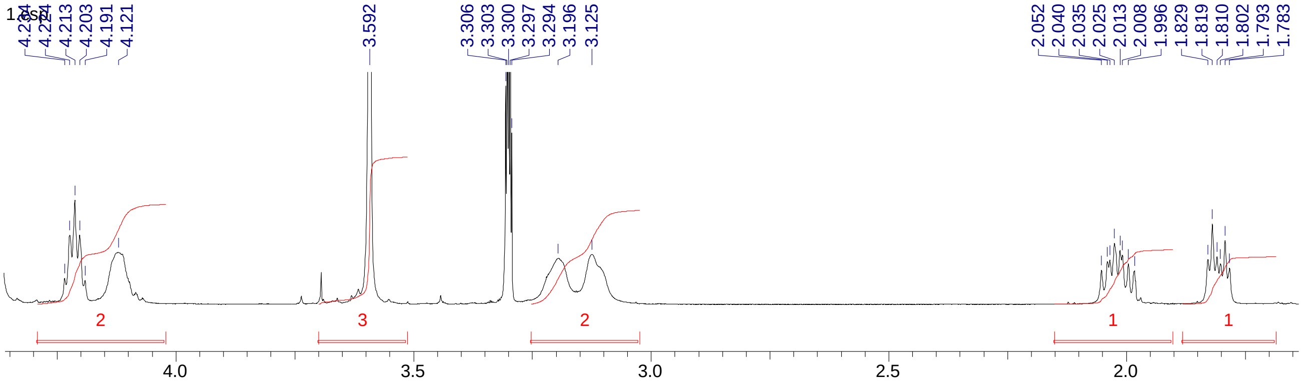

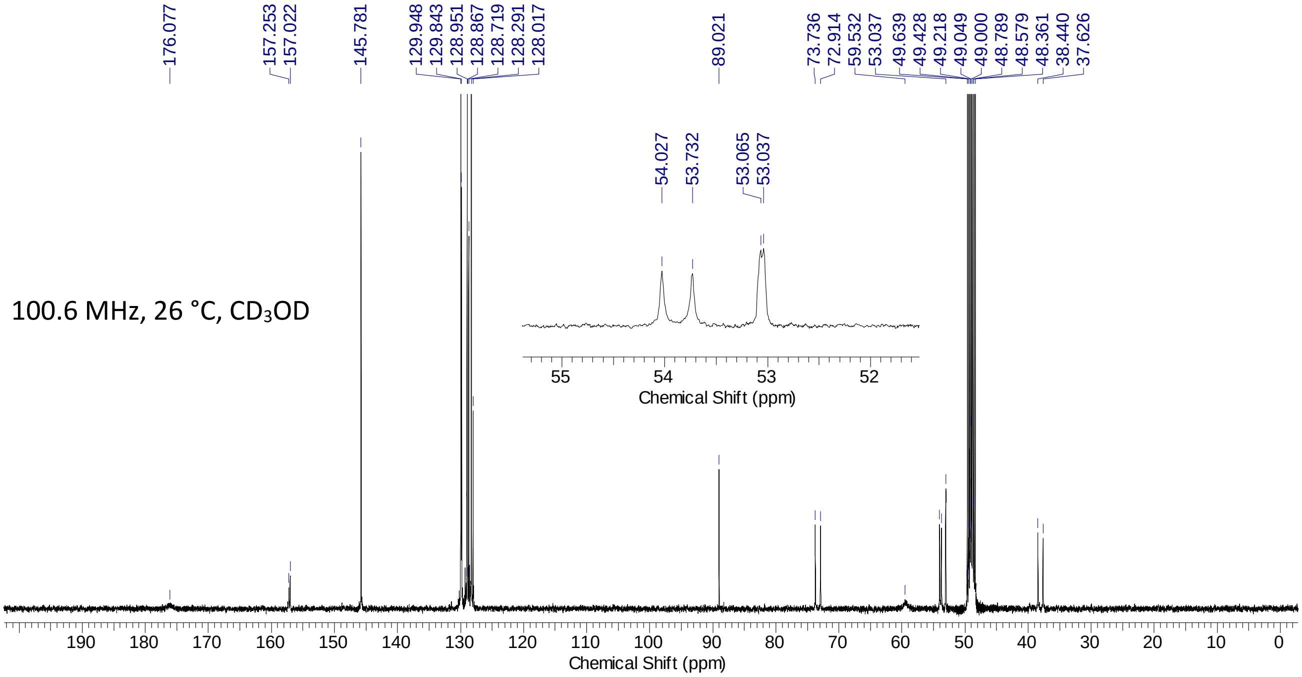
100.6 MHz, 26 °C, CD3OD
54.027
53.732
53.065
53.037
Chemical Shift (ppm)

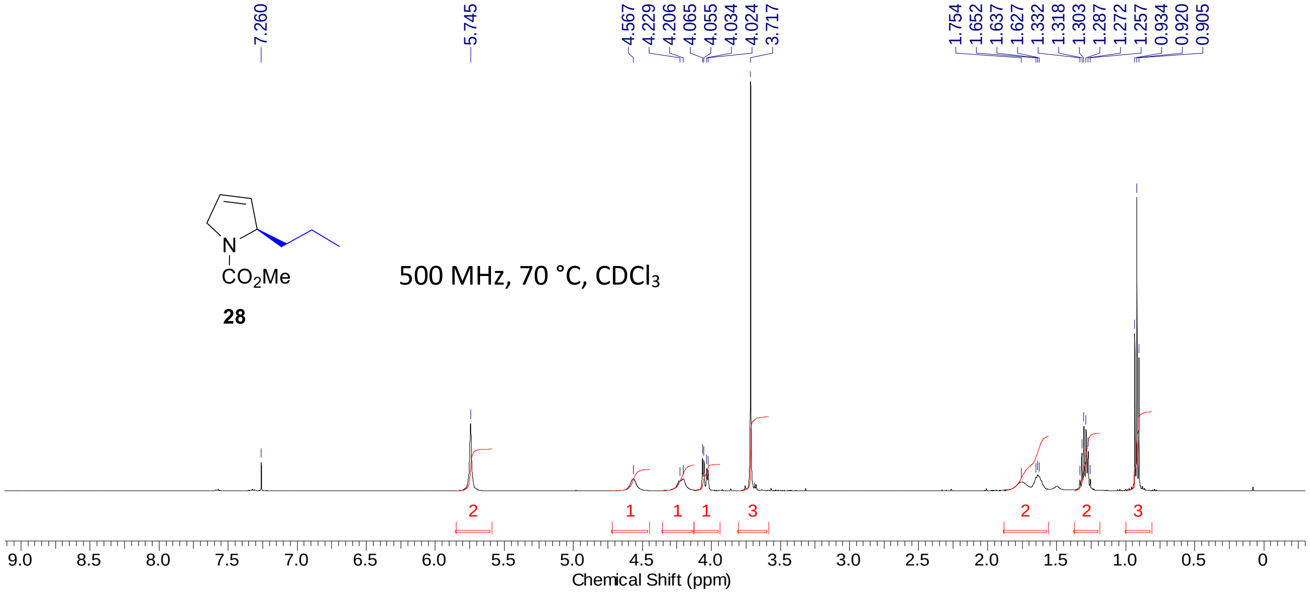
7.260
5.745
4.567
4.229
4.206
4.065
4.055
4.034
4.024
3.717
1.754
1.652
1.637
1.627
1.332
1.318
1.303
1.287
1.272
1.257
0.934
0.920
0.905
N
CO2Me
28
500 MHz, 70 °C, CDCl3
2
1
1
1
3
2
2
3
Chemical Shift (ppm)

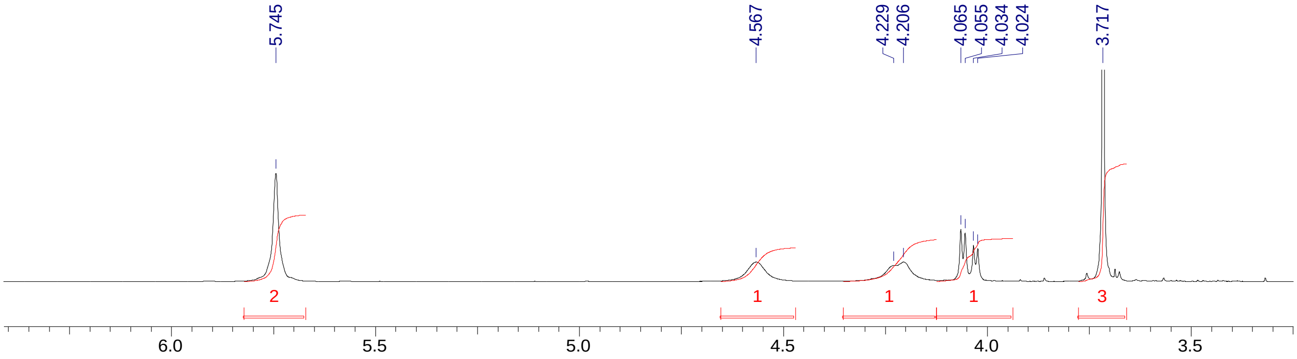
5.745
4.567
4.229
4.206
4.065
4.055
4.034
4.024
3.717
2
1
1
1
3

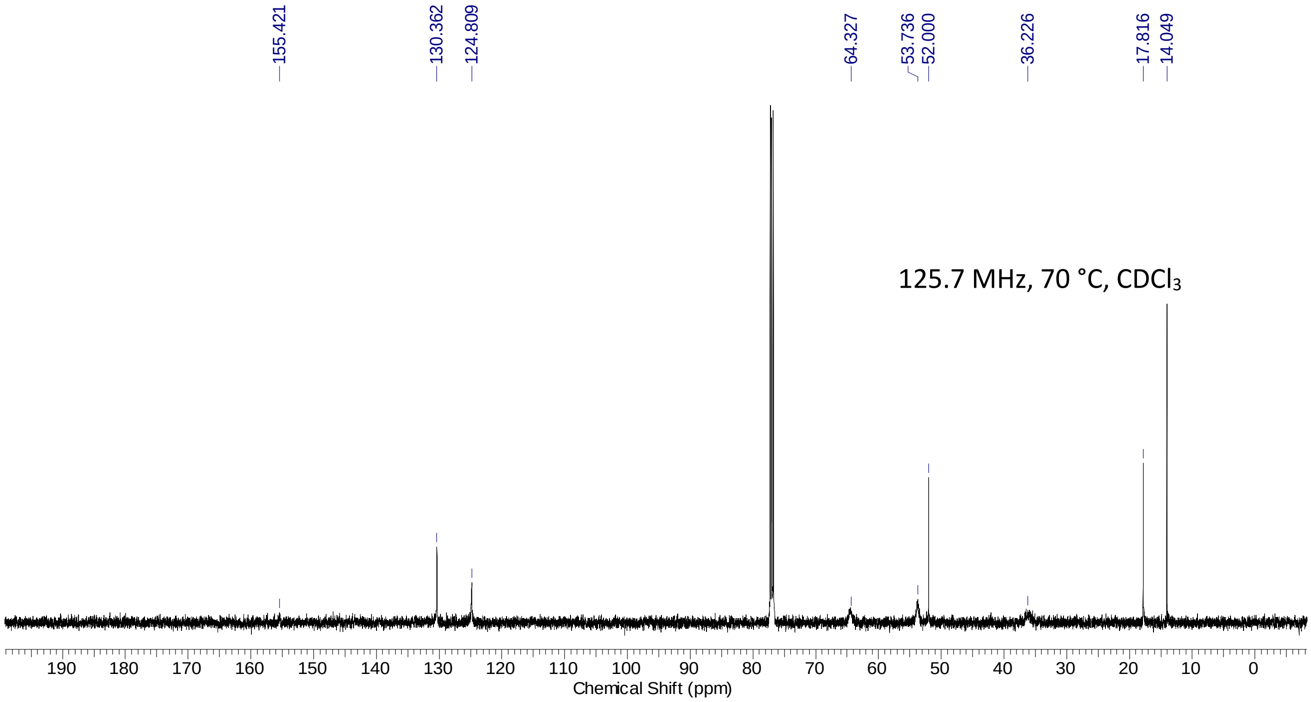
155.421
130.362
124.809
64.327
53.736
52.000
36.226
17.816
14.049
125.7 MHz, 70 °C, CDCl3
Chemical Shift (ppm)

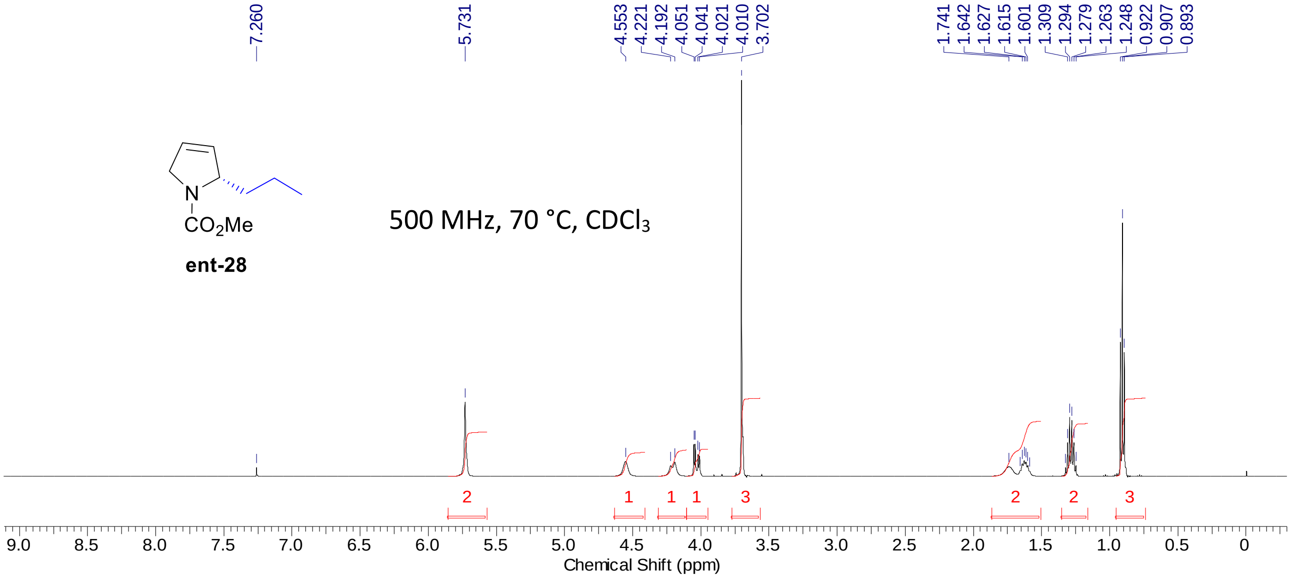

CO_2Me
ent-28
500 MHz, 70 °C, $CDCl_3$
7.260
5.731
4.553
4.221
4.192
4.051
4.041
4.021
4.010
3.702
1.741
1.642
1.627
1.615
1.601
1.309
1.294
1.279
1.263
1.248
0.922
0.907
0.893
2 1 1 1 3 2 2 3
9.0 8.5 8.0 7.5 7.0 6.5 6.0 5.5 5.0 4.5 4.0 3.5 3.0 2.5 2.0 1.5 1.0 0.5 0
Chemical Shift (ppm)

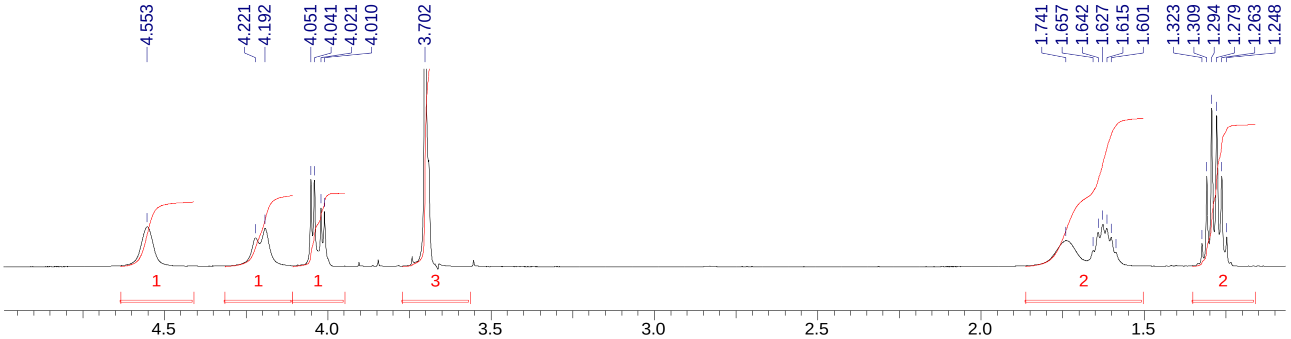

4.553
4.221
4.192
4.051
4.041
4.021
4.010
3.702
1.741
1.657
1.642
1.627
1.615
1.601
1.323
1.309
1.294
1.279
1.263
1.248
1 1 1 3 2 2
4.5 4.0 3.5 3.0 2.5 2.0 1.5

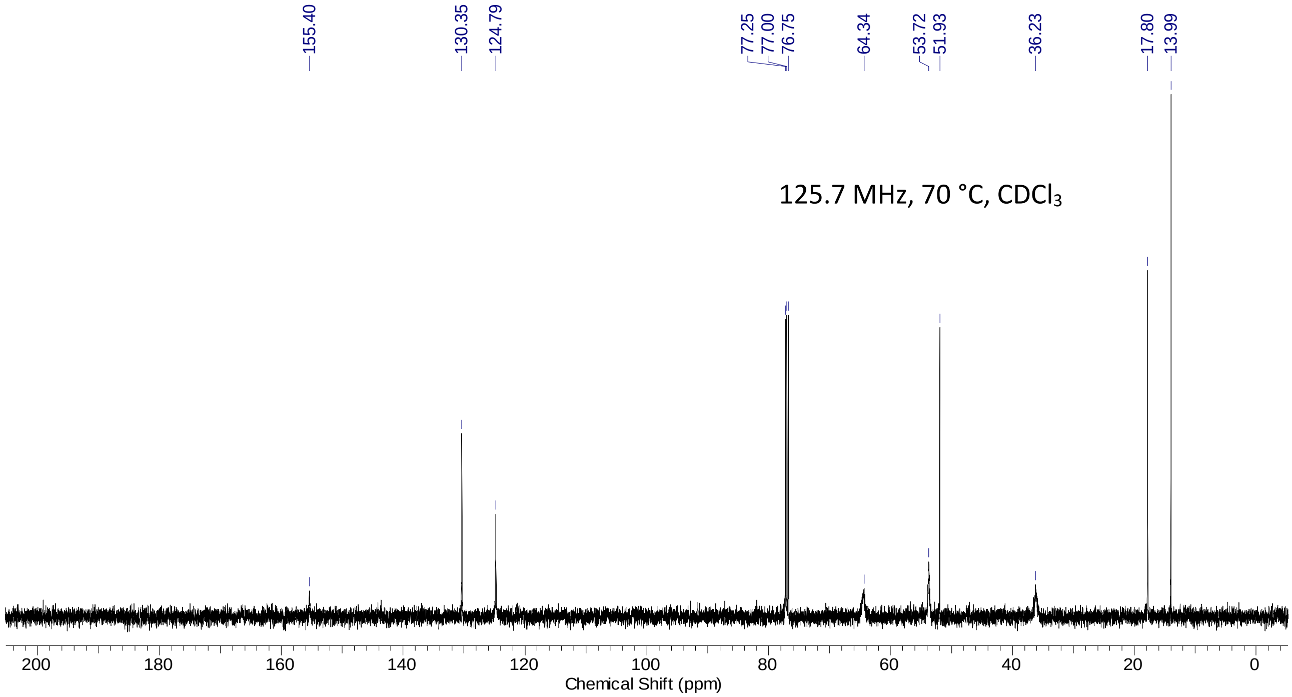

125.7 MHz, 70 °C, $CDCl_3$
155.40
130.35
124.79
77.25
77.00
76.75
64.34
53.72
51.93
36.23
17.80
13.99
200 180 160 140 120 100 80 60 40 20 0
Chemical Shift (ppm)

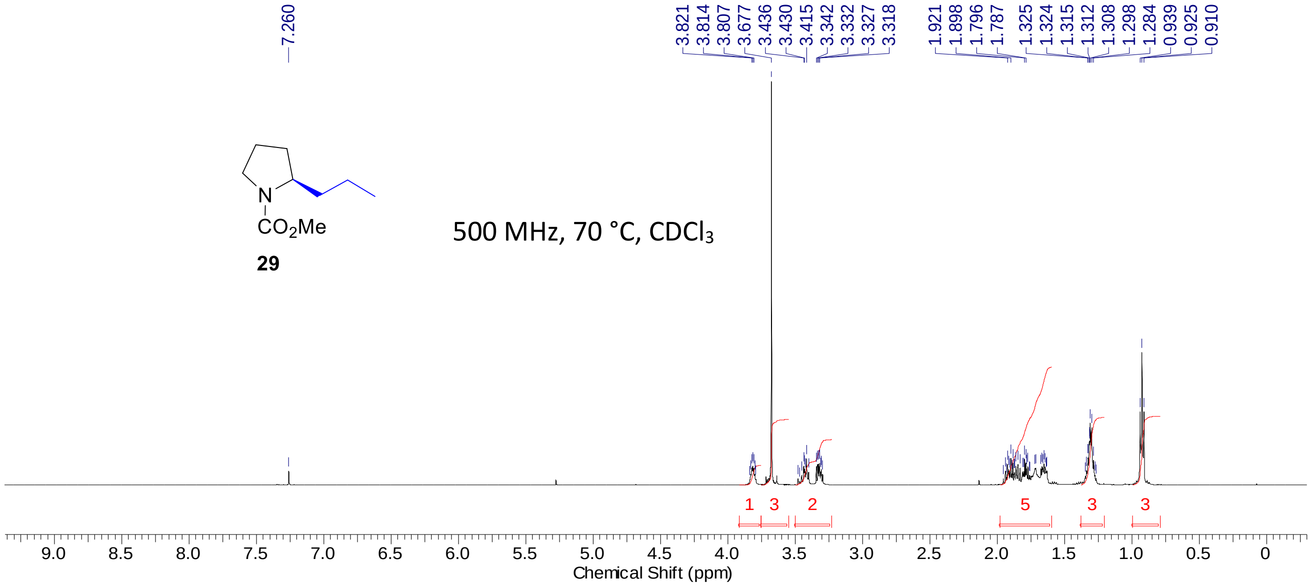
7.260
3.821 3.814 3.807 3.677 3.436 3.430 3.415 3.342 3.332 3.327 3.318
1.921 1.898 1.796 1.787 1.325 1.324 1.315 1.312 1.308 1.298 1.284 0.939 0.925 0.910
N
CO_2Me
29
500 MHz, 70 °C, $CDCl_3$
1 3 2 5 3 3
9.0 8.5 8.0 7.5 7.0 6.5 6.0 5.5 5.0 4.5 4.0 3.5 3.0 2.5 2.0 1.5 1.0 0.5 0
Chemical Shift (ppm)

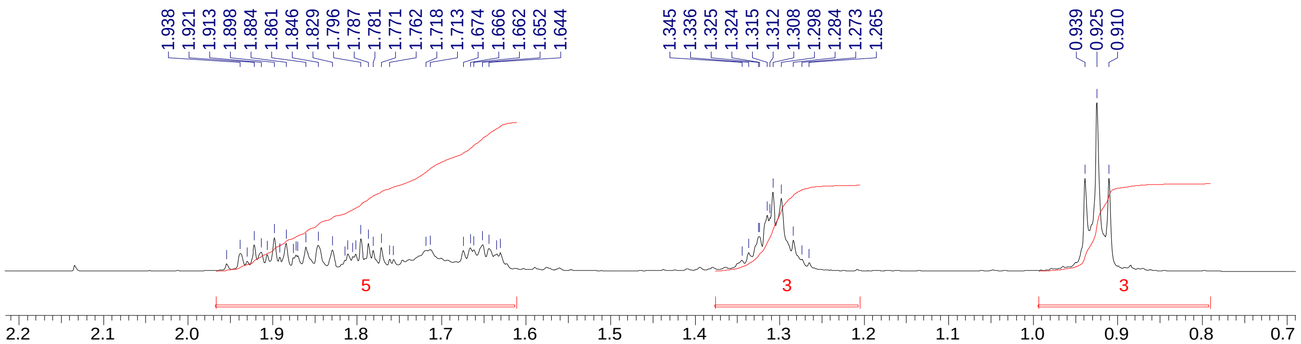
1.938 1.921 1.913 1.898 1.884 1.861 1.846 1.829 1.796 1.787 1.781 1.771 1.762 1.718 1.713 1.674 1.666 1.662 1.652 1.644
1.345 1.336 1.325 1.324 1.315 1.312 1.308 1.298 1.284 1.273 1.265
0.939 0.925 0.910
5 3 3
2.2 2.1 2.0 1.9 1.8 1.7 1.6 1.5 1.4 1.3 1.2 1.1 1.0 0.9 0.8 0.7

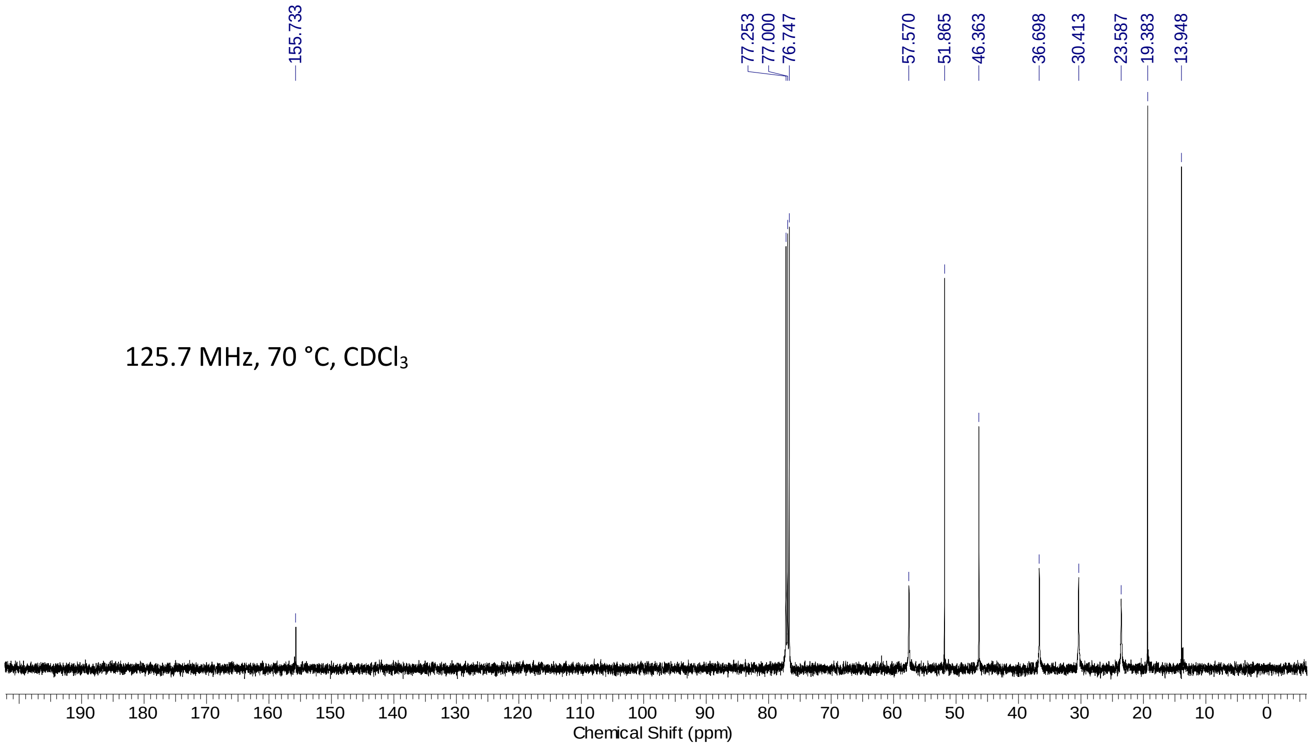
155.733
77.253 77.000 76.747
57.570 51.865 46.363 36.698 30.413 23.587 19.383 13.948
125.7 MHz, 70 °C, $CDCl_3$
190 180 170 160 150 140 130 120 110 100 90 80 70 60 50 40 30 20 10 0
Chemical Shift (ppm)

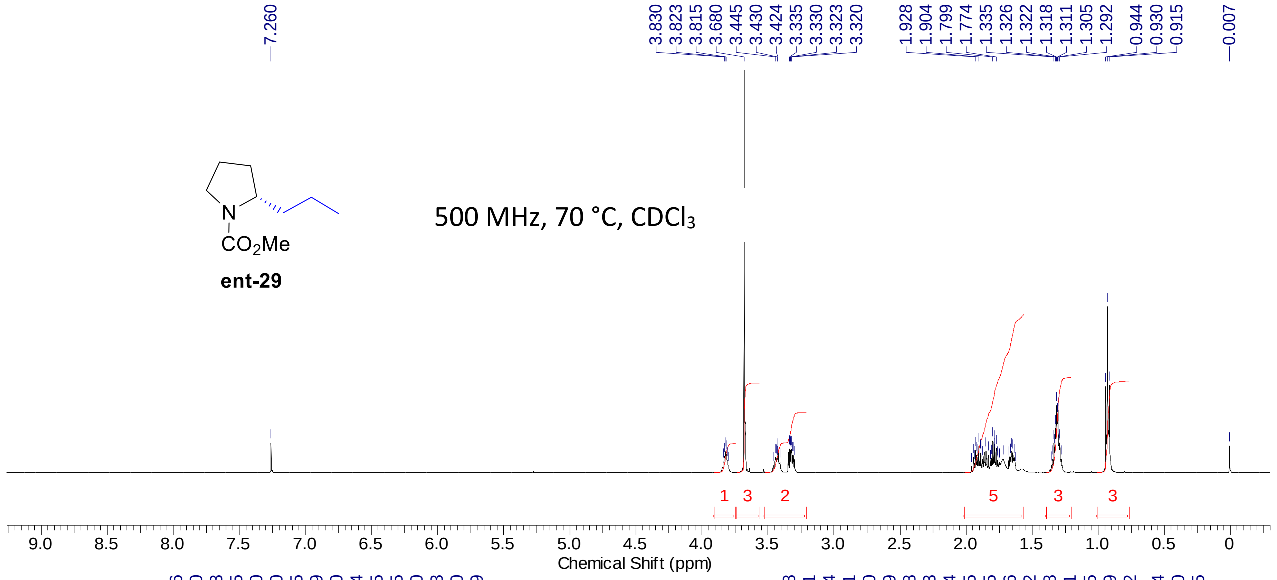
N
CO2Me
ent-29
7.260
3.830 3.823 3.815 3.680 3.445 3.430 3.424 3.335 3.330 3.323 3.320
1.928 1.904 1.799 1.774 1.335 1.326 1.322 1.318 1.311 1.305 1.292
0.944 0.930 0.915
0.007
500 MHz, 70 °C, CDCl3
1 3 2 5 3 3
9.0 8.5 8.0 7.5 7.0 6.5 6.0 5.5 5.0 4.5 4.0 3.5 3.0 2.5 2.0 1.5 1.0 0.5 0
Chemical Shift (ppm)

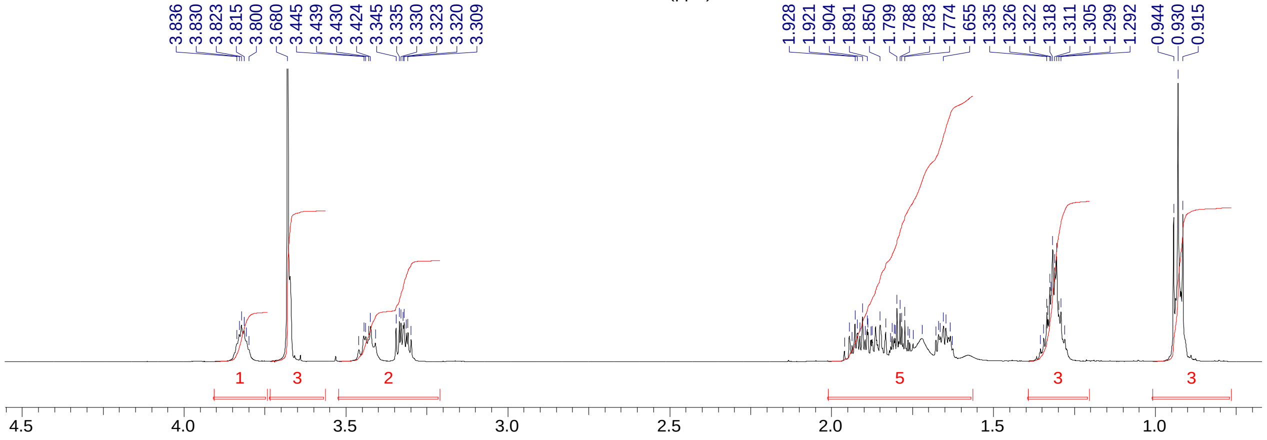
3.836 3.830 3.823 3.815 3.800 3.680 3.445 3.439 3.430 3.424 3.345 3.335 3.330 3.323 3.320 3.309
1.928 1.921 1.904 1.891 1.850 1.799 1.788 1.783 1.774 1.655 1.335 1.326 1.322 1.318 1.311 1.305 1.299 1.292
0.944 0.930 0.915
1 3 2 5 3 3
4.5 4.0 3.5 3.0 2.5 2.0 1.5 1.0

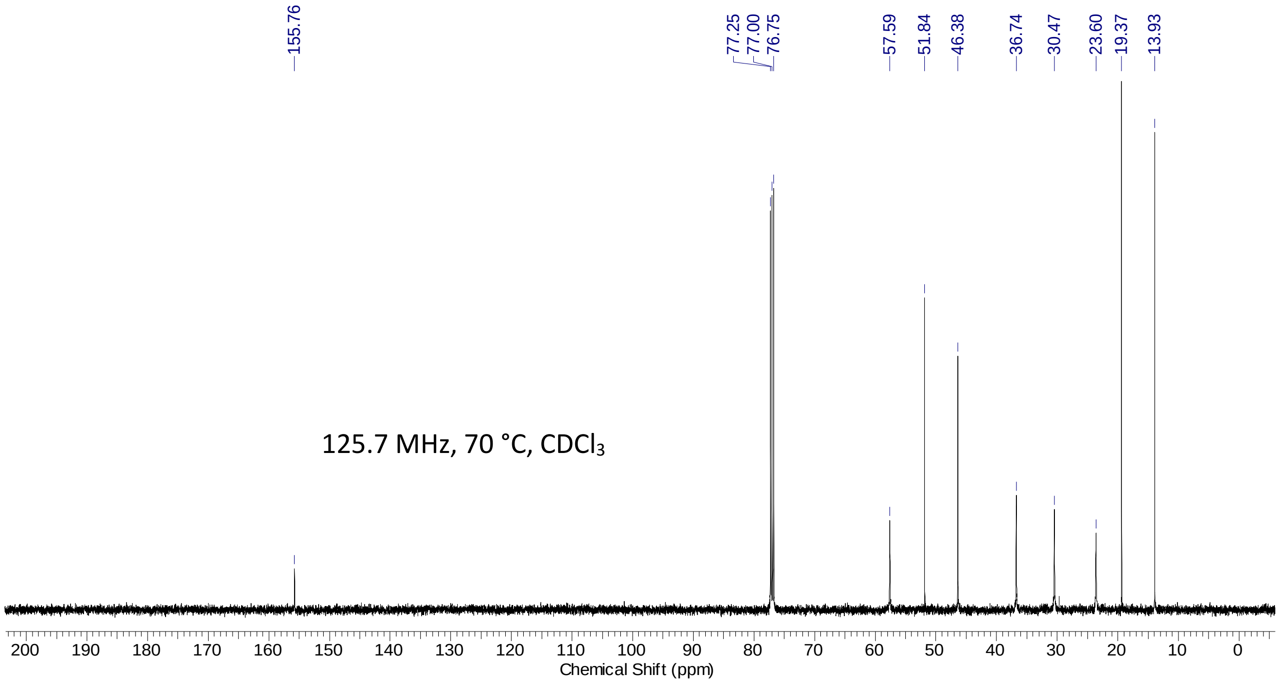
155.76
77.25 77.00 76.75
57.59 51.84 46.38 36.74 30.47 23.60 19.37 13.93
125.7 MHz, 70 °C, CDCl3
200 190 180 170 160 150 140 130 120 110 100 90 80 70 60 50 40 30 20 10 0
Chemical Shift (ppm)

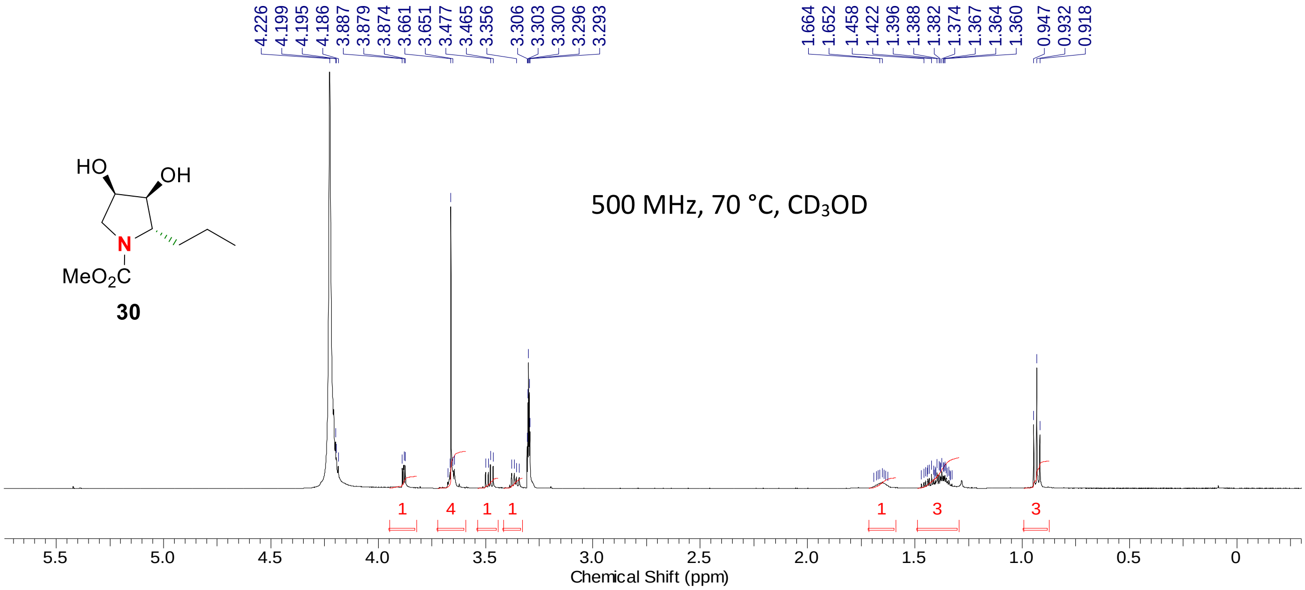
HO
OH
N
MeO2C
30
4.226 4.199 4.195 4.186 3.887 3.879 3.874 3.661 3.651 3.477 3.465 3.356 3.306 3.303 3.300 3.296 3.293
1.664 1.652 1.458 1.422 1.396 1.388 1.382 1.374 1.367 1.364 1.360 0.947 0.932 0.918
500 MHz, 70 °C, CD3OD
1 4 1 1 1 3 3
5.5 5.0 4.5 4.0 3.5 3.0 2.5 2.0 1.5 1.0 0.5 0
Chemical Shift (ppm)

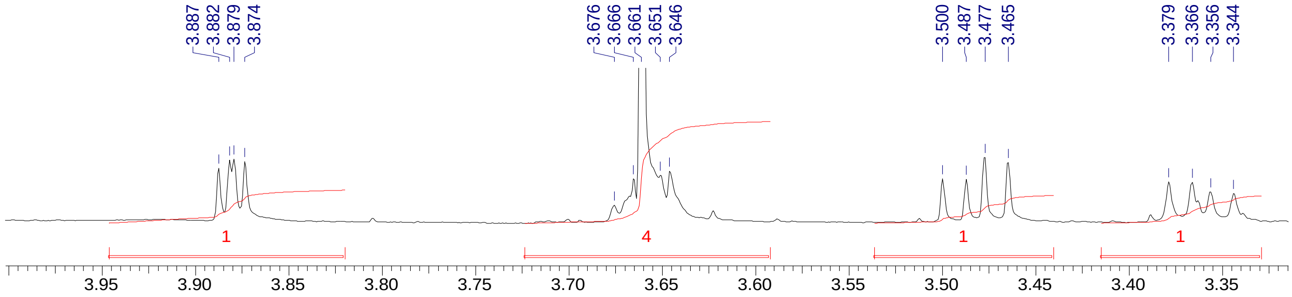
3.887 3.882 3.879 3.874
3.676 3.666 3.661 3.651 3.646
3.500 3.487 3.477 3.465
3.379 3.366 3.356 3.344
1 4 1 1
3.95 3.90 3.85 3.80 3.75 3.70 3.65 3.60 3.55 3.50 3.45 3.40 3.35

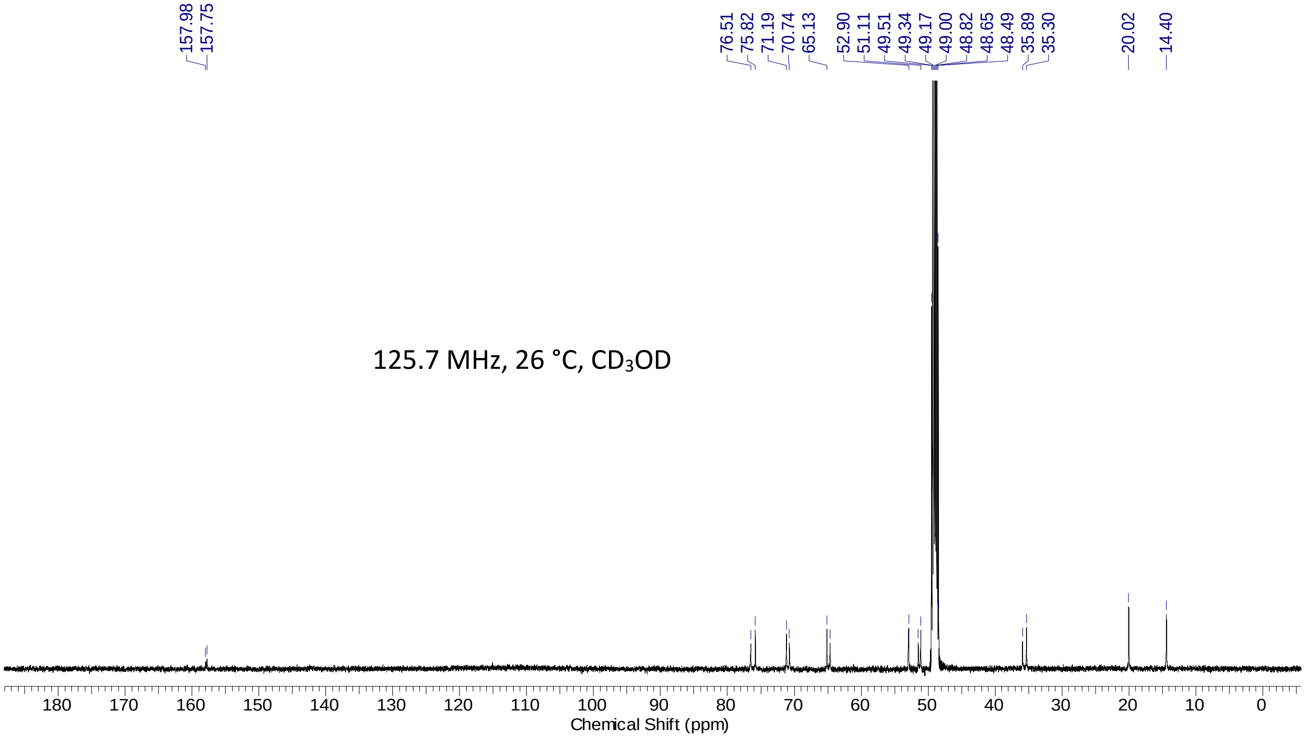
157.98 157.75
76.51 75.82 71.19 70.74 65.13 52.90 51.11 49.51 49.34 49.17 49.00 48.82 48.65 48.49 35.89 35.30
20.02 14.40
125.7 MHz, 26 °C, CD3OD
180 170 160 150 140 130 120 110 100 90 80 70 60 50 40 30 20 10 0
Chemical Shift (ppm)

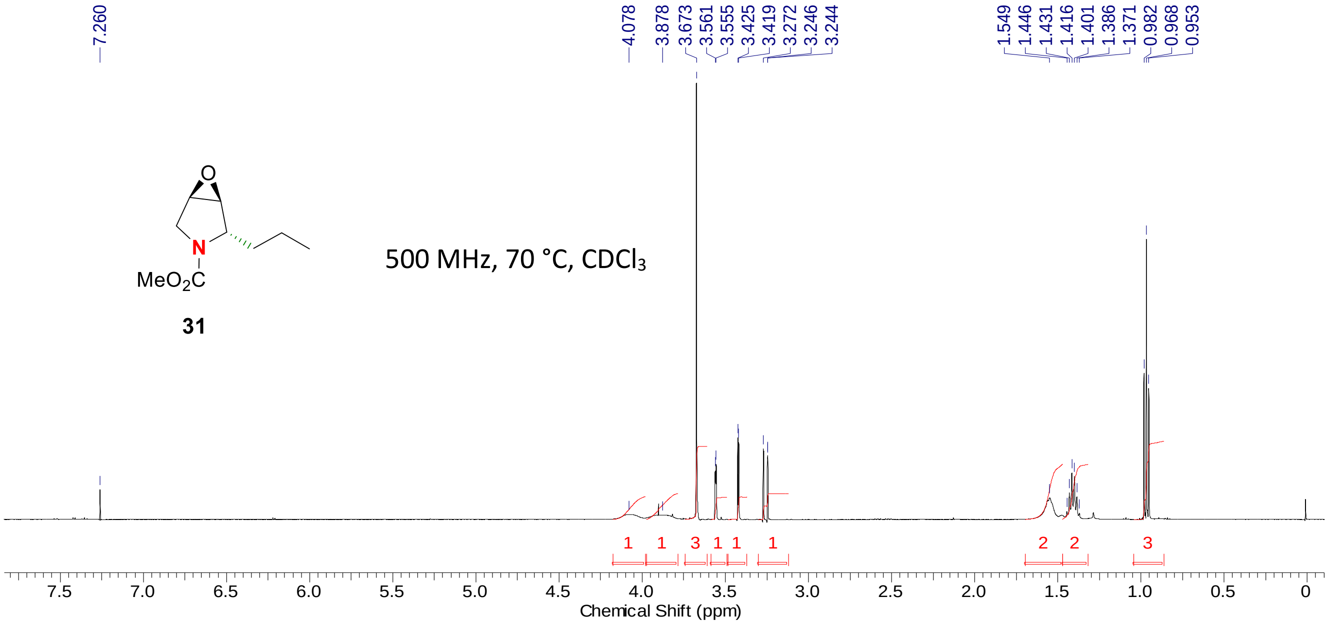
O
N
MeO$_2$C
500 MHz, 70 °C, CDCl$_3$
7.260
4.078
3.878
3.673
3.561
3.555
3.425
3.419
3.272
3.246
3.244
1.549
1.446
1.431
1.416
1.401
1.386
1.371
0.982
0.968
0.953
1 1 3 1 1 1 2 2 3
7.5 7.0 6.5 6.0 5.5 5.0 4.5 4.0 3.5 3.0 2.5 2.0 1.5 1.0 0.5 0
Chemical Shift (ppm)

31

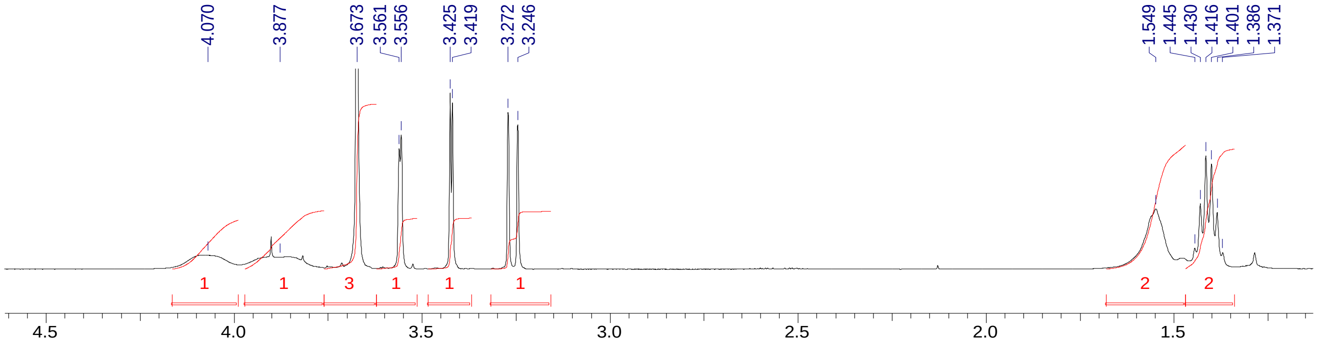
4.070
3.877
3.673
3.561
3.556
3.425
3.419
3.272
3.246
1.549
1.445
1.430
1.416
1.401
1.386
1.371
1 1 3 1 1 1 2 2
4.5 4.0 3.5 3.0 2.5 2.0 1.5

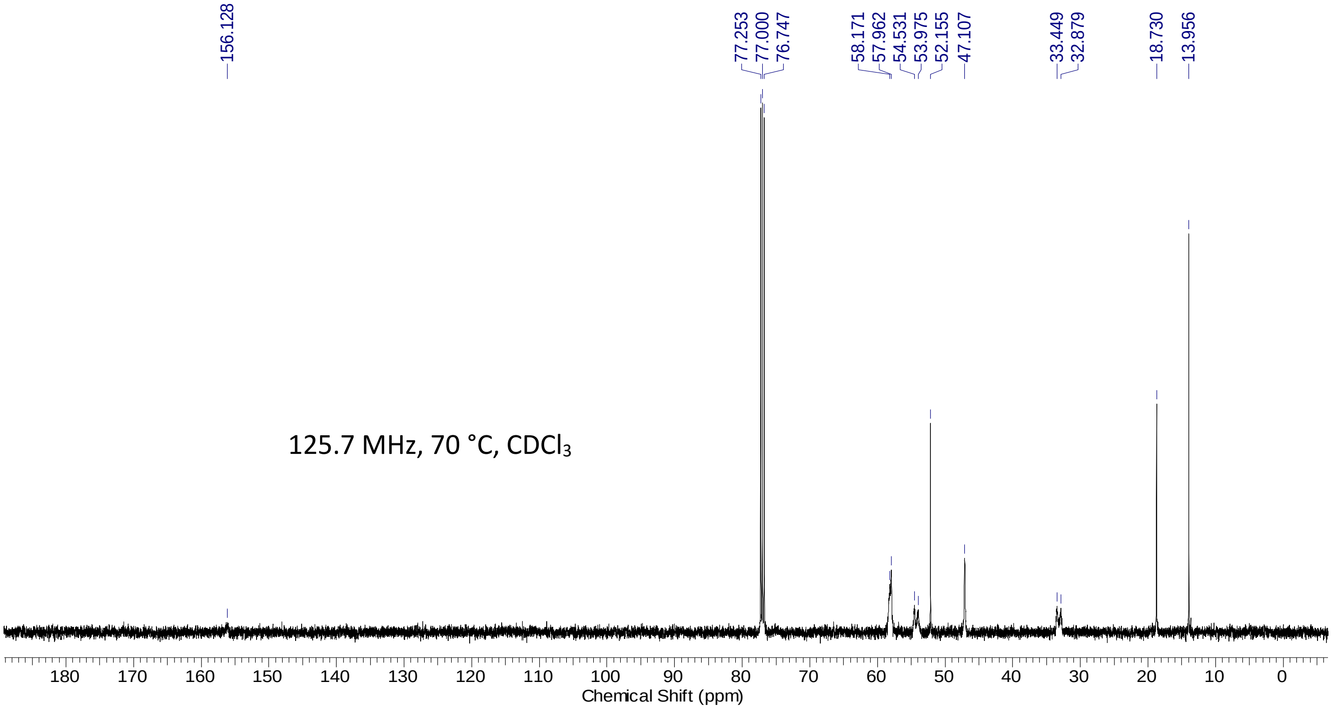
125.7 MHz, 70 °C, CDCl$_3$
156.128
77.253
77.000
76.747
58.171
57.962
54.531
53.975
52.155
47.107
33.449
32.879
18.730
13.956
180 170 160 150 140 130 120 110 100 90 80 70 60 50 40 30 20 10 0
Chemical Shift (ppm)

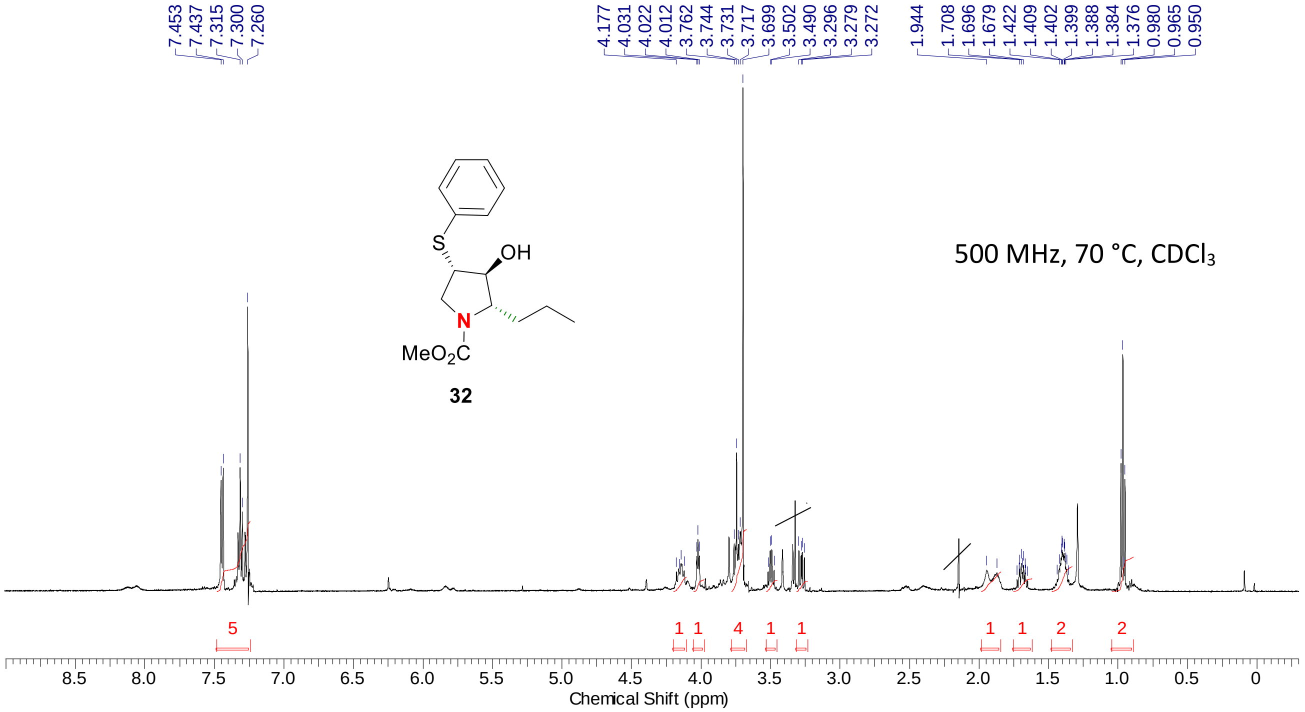
7.453 7.437 7.315 7.300 7.260
4.177 4.031 4.022 4.012 3.762 3.744 3.731 3.717 3.699 3.502 3.490 3.296 3.279 3.272
1.944 1.708 1.696 1.679 1.422 1.409 1.402 1.399 1.388 1.384 1.376 0.980 0.965 0.950
S
OH
N
MeO2C
32
500 MHz, 70 °C, CDCl3
5
1 1 4 1 1
1 1 2 2
8.5 8.0 7.5 7.0 6.5 6.0 5.5 5.0 4.5 4.0 3.5 3.0 2.5 2.0 1.5 1.0 0.5 0
Chemical Shift (ppm)

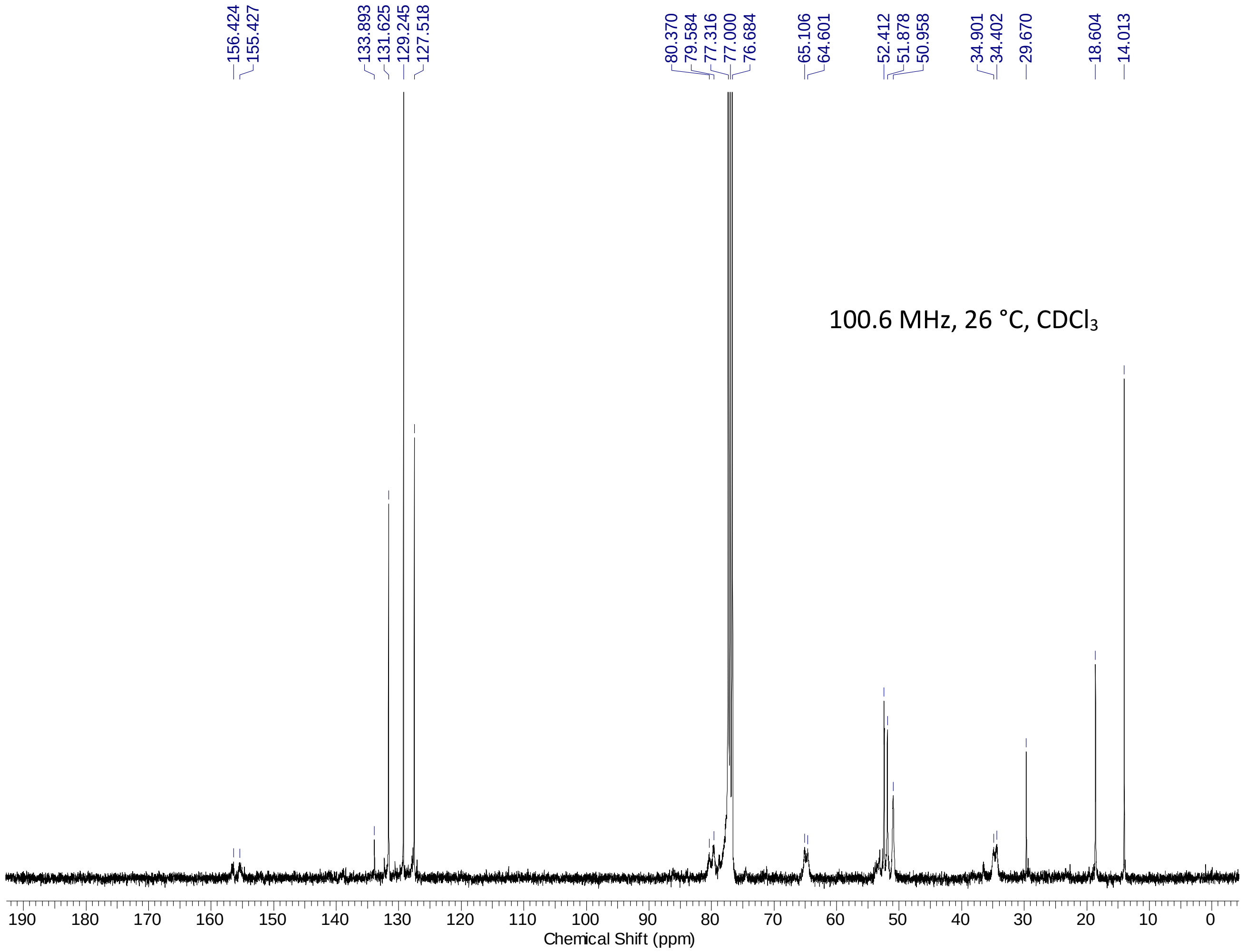
156.424 155.427
133.893 131.625 129.245 127.518
80.370 79.584 77.316 77.000 76.684
65.106 64.601
52.412 51.878 50.958
34.901 34.402 29.670
18.604 14.013
100.6 MHz, 26 °C, CDCl3
190 180 170 160 150 140 130 120 110 100 90 80 70 60 50 40 30 20 10 0
Chemical Shift (ppm)
